# Supplementary material for: Dated gene duplications elucidate the evolutionary assembly of eukaryotes
Source: Nature. 2025 Dec 3;650(8100):129–40. doi: 10.1038/s41586-025-09808-z (PMC12872463; doi:10.1038/s41586-025-09808-z)
Supplement: Supplementary file 1 — This file contains Supplementary Notes 1–4, Supplementary Discussion, Supplementary Figs. 1–12, Supplementary Tables 1–7 and Supplementary References. [file 41586_2025_9808_MOESM1_ESM.pdf]

nature portfolio

https://doi.org/10.1038/s41586-025-09808-z

Supplementary information

Dated gene duplications elucidate the evolutionary assembly of eukaryotes

In the format provided by the authors and unedited

Supplementary information

Supplementary Methods **3**
Supplementary Notes 1 - Expansion on manuscript methods 3
Software 3
Source data 3
Identifying candidate gene families (DO pipeline) 3
Composition of sequence group by colour 3
Spanning deep evolutionary time with HMMs 3
Deciphering complex gene family histories 4
Reference species 4
HMM sequence searching 4
Alignment 4
Sequence filtering and alignment optimisation 5
Sequence clustering 5
Guide tree traversal 6
Naming convention of data obtained from the DO pipeline 6
Generating single gene trees (DA pipeline) 7
Taxon subselection 7
Sequence renaming 7
Sequence inclusion or exclusion 7
Alignment and alignment optimisation 7
Trimming 7
Maximum likelihood tree construction 7
Tree reconciliation 8
Prior annotation 8
MCMCTree 8
Supplementary Notes 2 - Species tree construction 9
Building the concatenate 9
Species tree topology 9
Time resolution of the species tree with MCMCTree 9
Supplementary Notes 3 - Species tree sensitivity analyses 10
Sensitivity of inferred eukaryogenesis timescale to alternative tree topologies and calibrations 10
Key node sensitivity tests 11
Supplementary Notes 4 - Species tree calibration information 12
Fossil calibrations 12
Speciation node age calibrations from the species tree 12
Additional derived calibrations 12
Supplementary Discussion **13**
Supplementary Discussion 1 - Extended commentary on specific systems 13
Additional biological systems figures. 13
Nucleus 13

DNA Replication ... 13
Transcription ... 13
Splicing and RNA processing ... 14
Histones ... 15
Chromatin ... 15
SMC family proteins ... 16
Nuclear import and export ... 16
Kinetochore ... 16
Subnuclear structures ... 16
Exosome complex ... 17
DNA repair ... 17
Meiosis ... 17
Membrane biology ... 17
Prenylation ... 17
Protein turnover and biogenesis ... 18
Proteasome ... 18
Ubiquitin ... 18
Translation ... 18
Supplementary Figures ... 19
Supplementary Figure 1. Time-resolved species tree. ... 19
Supplementary Figure 2. Nuclear processes- DNA replication and transcription. ... 20
Supplementary Figure 3. Nuclear processes- splicing, RNA processing and genome organisation. ... 21
Supplementary Figure 4. DNA repair system. ... 23
Supplementary Figure 5. Ubiquitin system. ... 24
Supplementary Figure 6. Ribosome and translation system. ... 25
Supplementary Figure 7. Flow diagram of the Domain Origins (DO) pipeline. ... 26
Supplementary Figure 8. Colour system used in the DO pipeline. ... 27
Supplementary Figure 9. HMM search figure from the DO pipeline. ... 27
Supplementary Figure 10. Alignment optimization figure from the DO pipeline. ... 28
Supplementary Figure 11. Guide tree traversal in the DO pipeline. ... 29
Supplementary Figure 12. Flow diagram of the Domain Analysis (DA) pipeline. ... 30
Supplementary Figure 13. MCMCTree node convergence plot. ... 31
Supplementary Tables ... 32
Supplementary Table 1. Software used in this study. ... 32
Supplementary Table 2. Source data, naming, and taxonomy information ... 33
Supplementary Table 3. Genes of the species tree concatenate ... 43
Supplementary Table 4. Species tree node age sensitivity tests. ... 45
Supplementary Table 5. Applying test constraints to key species tree nodes. ... 46
Supplementary Table 6. Fossil calibrations. ... 47
Supplementary Table 7. Our investigated gene families and their duplication dates ... 49
Supplementary Bibliography ... 88

Supplementary Methods

Supplementary Notes 1 - Expansion on manuscript methods

In the following, we provide additional details beyond those in the main text Methods section, focussing particularly on the two pipelines created in this study.

Software

All software used independently or as part of pipelines are listed with their version numbers in **Supplementary Table 1**. We highlight calls to specific programs, scripts, or named files by formatting them with `monospaced text`.

Source data

With the exception of a few prokaryote species, we used publicly available predicted protein sets from either NCBI or Eukprot. When unavailable, we predicted open reading frames from publicly accessible genomes using `TransDecoder.LongOrfs`. In addition these proteomes were further processed by removing sequences of less than 50aa (unlikely to be useful in our analysis) and deduplication of 100% identical sequences, using `cd-hit` (-c 1.0). The data source, and whether proteomes were predicted are indicated for every taxon in **Supplementary Table 2.** A further description of the fasta headers used for proteomes in our set is given in `readme_fasta_headers.txt.`

Identifying candidate gene families (DO pipeline)

The Domain Origins (DO) pipeline was made to aid in determining which gene families have histories relevant to the scope of this project. This section will introduce some concepts built into the pipeline before expanding on the steps in the order of the Methods section. A flow diagram of the pipeline is shown in **Supplementary Figure 7**, additional requirements of the pipeline and its dependencies are found in the `readme.txt` file associated with the script.

Composition of sequence group by colour

A group sequence colour system was developed to guide gene family exploration. Eukaryotes, Bacteria, and Archaea are assigned GREEN, BLUE, and RED axes respectively. Single-domain groups have primary colours; mixed-domain groups have secondary colours (ORANGE, CYAN, PURPLE). Groups with all three domains receive WHITE. A 5% margin applies for primary colour assignments (e.g., >=95% eukaryotic for GREEN). Ternary plots in **Supplementary Figure 8** illustrates this principle and colour calls direct DO pipeline operations.

Spanning deep evolutionary time with HMMs

The existing HMMs from the publicly available databases are often built on a limited number of model organisms, and were sometimes insufficient to identify orthologs spanning between the supergroups. Therefore we rebuild the HMMs as part of an iterative process in the pipeline. Furthermore we found value in rebuilding the domains with respect to their supergroups, for example where a limited archaeal outgroup was detected, it proved better

to build separate archaeal and eukaryotic HMMs. Both HMMs could then be used in the iterated search.

Deciphering complex gene family histories

Protein domains are conserved, short sequences and often have complex evolutionary histories. Frequently it was difficult to produce alignments with any conserved positions, making the determination of protein domain origin difficult. Our solution was to coarsely cluster the protein domain sequences using an all-versus-all `BLAST` or `DIAMOND` search followed by clustering these results with `MCL`. The sequence subsets had much better alignments and produced more confident trees, than the trees produced from alignments of the whole sequence pool.

Reference species

We found it helpful to use a reference species (*Homo sapiens*) to focus on gene families with annotated localisation and functional characterisation. The pipeline considers sequences from the reference species as privileged and will attempt to determine origins for any that occur in HMM searches.

The description of the DO pipeline will now follow the order set out in the Methods, elaborating on settings used. The web reports generated by DO pipeline also follow a similar order and example figures are referenced in the text below.

HMM sequence searching

Proteome sequence files were searched using `hmmer`:

```
hmmsearch --noali --domtblout
```

1. The resulting output table was filtered with `awk`.
2. Sequences were required to cover at 75% of the HMM model
3. Sequences were deduplicated by species with `cd-hit` (-c 1.0)
4. HMM search results were binned into good (all sequences with an e-value < 1e-6) or weak categories (all sequences with an e-value < 1e-2), ‘good’ sequences were used to rebuild the HMM for the iterative search.

The results of the HMM search is one of the figures in the DO pipeline web reports, an example is shown in **Supplementary Figure 9**. When the HMM search is iterated with new HMMs, the search pool is the weak scoring sequences bin. This step was necessary as rebuilding HMMs for domains which belong to large superfamilies often recovered the superfamily on the second iteration.

Alignment

Sequence alignment in the pipeline were based on the program `MAFFT`. Whenever the number of sequences is less than 100,000 the `fftnsi` is used, otherwise `fftns`, to balance accuracy and computational tractability.

Sequence filtering and alignment optimisation

Filtering and alignment optimisation steps occur at multiple points in the pipeline after each `mafft` alignment. Filtering involves a coarse filter (written in `awk`), and a fine filter with alignment optimisation (perl script `Maxalign.pl`).

Coarse filter:

The coarse filter is applied first, it is based on transforming the protein sequence alignment into a column occupancy matrix, which has the format:

- If there is a column which only has one row with an amino acid position, its value is one.
- If there is a column where there is occupancy in every row, its value regardless of amino acid, is equal to the number of rows

The coarse filter then operates on this transformed matrix, to remove rows which:

- Have less than 80% the average row sum
- Have less than 80% the average column score
- Have more than 10% of columns having less than 10% occupancy
- Has more than 10% of columns that break those of more than 90% occupancy

After poorly aligned sequences were removed, the sequences were realigned with `MAFFT` as described above.

Maxalign

The `Maxalign` script seeks to optimise an input alignment and identify sequences to remove, it is run with the default parameters.

Outputs from filtering and alignment optimisation comprise one of the visual outputs of the pipeline on its web report pages, an example is shown in **Supplementary Figure 10**. As sequences are removed in the filtering process, the group colour (as described above) is checked before and after, if there is a colour change in the filtering process the pipeline defaults to the original alignment.

Sequence clustering

To perform the sequence clustering we used an all versus all sequence homology search (`BLAST` where the number of sequences < 5,000, otherwise `DIAMOND`) to produce a list of sequence pairs and their values. This information is then passed to `MCL` which clusters the sequences into groups. We found that a low inflation value (I=1.2) for `MCL` was suitable for all our analyses. The group colour of the resulting clusters then informs pipeline direction:

- If a cluster contains a reference sequence, eukaryote and prokaryote sequences (ORANGE, CYAN, WHITE), the cluster alone likely contains the origin information for that reference sequence.
- If a cluster contains a reference protein and only contains eukaryotes (GREEN), but the total of clusters is ORANGE, CYAN or WHITE, then more than this cluster needs to be considered when determining the origin for the reference protein in this cluster. If there are only two clusters, the total sequence pool is carried forward. If there are three or more clusters, a guide tree traversal approach is used.

Guide tree traversal

A representative of each cluster (the sequence with the median all versus all sequence score of the cluster) is chosen, these are pooled and an unrooted guide tree produced using IQ-tree. In summary, for each reference containing cluster, the guide tree is traversed from cluster of origin to next nearest cluster (by cumulative branch length) until the colour of the collected cluster sequences becomes ORANGE, CYAN or WHITE.

Worked example:

An example guide tree is shown in **Supplementary Figure 11**, in this example 3 reference sequences are found, A,B and C, each of which belong to groups which only contain eukaryotic sequences (GREEN). Representatives of each cluster are used to construct a guide tree. In this example reference proteins A and B traverse the tree to the same archaeal sequence cluster (RED), their 'paths' or the sequence of visited clusters share the same clusters albeit in a different order. Where reference eukaryotes from separate clusters are found on the same path, they are taken together to produce a sequence pool composed of the sequence clusters on this 'path'. In the example, the path containing clusters 1,4 and 6 produces a path containing a pre-LECA duplication of archaeal origin.

Naming convention of data obtained from the DO pipeline

Sequence sets identified by the DO pipeline fall into 3 different categories which are carried forward in their naming:

- Sequences from the whole sequence pool
- Sequences obtained from an MCL cluster (suffix C<number>)
- Sequences pooled from clusters along a 'path' (suffix P<cluster numbers>)

For each data type, complete ORFs or just domain sequences are possible outputs, and hyperlinks to these files are generated in the web reports. Typically we used the entire reading frame rather than just the query domain, which is shown in the naming convention of the data (ORFs).

Generating single gene trees (DA pipeline)

This section covers the operation of the Domain Analysis (DA) pipeline, which takes input fasta sequence files from the DO pipeline, generates a maximum likelihood tree (Phase 1), and then optionally time-resolves them (Phase 2). The initial tree construction shares many steps in common with the DO pipeline. The first phase of this pipeline was used to prepare the genes for the species tree concatenate, however the time resolution of the species tree was run in a separate script although the MCMCTree parameters are the same as in phase two. Additional requirements of the pipeline are specified in the `readme.txt` in the same folder as the script, a summary flow diagram of the pipeline is given in **Supplementary Figure 12**.

Taxon subselection

Our species tree does not contain all the species in the original DO pipeline search pool, before further analysis, the taxa in the input files are subsampled to just those appearing on our species tree (listed in `SMALL_LUT.txt`). This simplifies the process of node annotation for MCMCTree.

Sequence renaming

Sequence headers are renamed to simplify processing and manual sequence removal. All sequences begin with a short unique numerical string, followed by the species name. The associated script `fast_sequence_excluder.sh` allows users to add sequences to inclusion or exclusion lists, and can use these short leading numbers as input.

Sequence inclusion or exclusion

In each work folder the pipeline creates two files: `excluded_sequences.txt`, `included_sequences.txt` which contain fasta headers to be omitted from analysis, or added back into the alignment at the final alignment stage.

Alignment and alignment optimisation

Is performed in the same way as in the DO pipeline, alignments are first coarsely filtered, optimised with `Maxalign`.

Trimming

After alignment optimisation trimming is performed with `bmge`;

```
bmge -i <input> -t AA -m BLOSUM30 -of <output> -g 0.75 -b 1 -h 1
```

We found high entropy values (-h 1) were required, using lower values produced extremely few conserved columns, even in highly conserved and well aligned proteins.

Maximum likelihood tree construction

The final stage of phase 1 is to produce a maximum likelihood tree. This tree is carried forward for species tree reconciliation, rooting (optional) and node prior annotation. The tree

model is first selected through IQ-tree `modelfinder`:

```
iqtree -s <input> -mset LG -mrate I,F,G,I+G --cmax 5 -nt $threads -m
TESTONLY -redo --prefix "$fasta_name."
```

We choose to limit to the LG model set because of `MCMCTree's` limited support for other more complex models, and to minimise model inconsistency between this phase of the pipeline and the next. After model selection the final maximum likelihood tree is constructed using:

```
iqtree -s <input> -m $model -nt AUTO -bb 10000 --nmax 500 --nstep 20
--prefix "$fasta_name"
```

Tree reconciliation

Maximum likelihood trees need to be rooted before `MCMCTree` is run, this can either be performed manually (treerecs mode = 0), or with the tree reconciliation program `Treerecs`, to simply root the tree (treerecs mode = 1) or to both root and reconcile the tree (treerecs mode = 2), these arguments can be specified on running the DA pipeline. If `Treerecs` is used it will ask for a named tree from the species trees file. The user choice for tree reconciliation for each investigated is shown as a column in Supplementary Table 7.

Prior annotation

Internal nodes on the rooted ML tree are converted to species tip lists, these are compared to the calibrations and the included and excluded node lists to determine which calibrations fit the list of taxa. If a calibration can be applied, the internal node is modified to accommodate a `MCMCTree` formatted calibration.

MCMCTree

MCMCTree/CODEML requires control files printed by the pipeline, these are defined by the functions towards the top of the pipeline called `prepare_codeml_control_file ()`, `prepare_substitution_control_file ()`, `make_MCMCtree_control_file ()`. Analysis chains are performed in quadruplicate with the same burn in (10,000) and sampling (20,000, sampling frequency 1 in 2) as the species tree.

Supplementary Notes 2 - Species tree construction

This section covers the production of the time resolved species tree, and defining the FECA and LECA nodes in this study. This section is broken down into building the sequence concatenate, determining the species tree topology, and time resolution of the species tree.

Building the concatenate

Genes suitable for building the species tree were identified from the DO pipeline (**Supplementary Table 3)**, containing 21 genes with a LUCA ancestor, 35 genes with eukaryote descent from Archaea, 31 genes with eukaryote descent from Bacteria, and 27 genes with plastid descent from Oxyphotobacteria (**Supplementary Table 3**). Two scripts were then used to compile the concatenates, the first script (`sequence_gatherer.sh`) renames eukaryotic tips based on their prokaryotic descent and enumerates them, the second script (`concatenation_builder.sh`) individually aligns the sequences of each gene using `MAFFT (linsi --retree 2 --maxiterate 1000 --bl 30)` and then trims the alignment with BMGE (`bmge -i <alignment> -t AA -m BLOSUM30 -h 1 -g 0.6`), the script then concatenates the individual alignments and prepares the .phy alignment file for `MCMCTree`. Extended documentation for both of these scripts can be found in the `readme` in the same folder as the scripts.

Species tree topology

The species tree concatenate was used to produce several additional subset concatenates to investigate the position of eukaryotic clades within archaea and bacteria (containing nuclear or mitochondrial eukaryote clades and either Archaea or Bacteria, respectively), and the position of the prokaryotic roots (subset of the concatenate only containing prokaryotes). The maximum likelihood trees produced are consistent with current consensus views, with eukaryotes branching with Heimdallarchaeia and Alphaproteobacteria. However, support values for these positions were typically low in the trees, so we performed a series of constrained tree searches and AU tests to evaluate the support for various alternative hypotheses. Overall, we did not obtain any compelling support for alternative species relationships, and therefore used the full maximum likelihood tree for subsequent molecular clock analyses. Further description of these investigations, including models and tests is given in the **Supplementary Data**, summarised in `Topology_investigation_summary.docx` and the associated tests in `Topology_tests.xslx`, these files and the trees and sequence concatenates necessary to reproduce these results are found in the folder `species_tree_topology.`

Time resolution of the species tree with MCMCTree

MCMCTree has a limited number of supported substitution models. To avoid model discordance we used the LG substitution matrix with 5 gamma rate categories for our maximum likelihood tree construction and MCMCTree time resolution. Our sampling regime involved a burn in of 10,000 and a sampling of 20,000 with a sampling frequency of 1 in 2. An expanded image of the time resolved species (of **Fig. 1A**) is shown in **Supplementary figure 1**. In the **Supplementary Data** we provide some additional files (.pdfs and treefiles,

as well as a visualised report of the MCMCTree runs) illustrating these results in the folder `time_resolved_species_tree`.

Supplementary Notes 3 - Species tree sensitivity analyses

Since the dated species tree is an important source of information for the inference of duplication ages, we performed a series of sensitivity tests to determine the impact of tree topology and node calibrations on our time-resolved species tree. This section summarises those findings.

Sensitivity of inferred eukaryogenesis timescale to alternative tree topologies and calibrations

In the first set of tests we explored a number of tree topological and calibration alternatives which might affect our eukaryogenesis timeline, specifically the effect of using an unconstrained bacterial root, over the asserted root in our species tree, the effect of the total-group Oxyphotobacteria calibration on the age of LBCA, and the effect of current competing eukaryotic root topologies (Metamonad[1], Opimoda / Diphoda[1,2]) on the age of LECA. These tests were performed by producing modified input files for `MCMCTree` and time-resolving them under the same conditions as the species tree. These results are tabulated in **Supplementary Table 4**, in summary our findings indicated:

- In all test conditions the age of LUCA tended towards the maximum constraint (~4.50 Ga).
- The unconstrained bacterial tree results in an LBCA of the same age to a similarly calibrated but Gracilicute/Terrabacteria rooted tree (3.24 versus ~3.22 Ga). However, it results in moderately younger ages for several key nodes; nFECA (2.63 versus 2.89 Ga), mFECA (1.96 versus 2.31 Ga), and LECA (1.57 versus 1.75 Ga), as well as a greatly younger age for the Gracilicute / Terrabacteria divergence (2.49 versus 3.22 Ga).
- The total Oxyphotobacteria calibration has an effect of slightly raising LBCA age (for the Opimoda / Diphoda eukaryote root, 3.22 to 3.36 Ga), although the effects on FECA and LECA ages are minimal (for the same topology, nFECA 2.89 to 2.92 Ga, mFECA 2.31 to 2.30 Ga, LECA 1.75 to 1.77 Ga).
- Of the tested eukaryote root topologies, the Metamonad root produces somewhat older LECA ages (for the Oxyphotobacteria calibrated trees, 1.90 versus 1.75 Ga), although comparable FECA ages (nFECA 2.87 versus 2.92 Ga, mFECA 2.27 versus 2.30 Ga).

As a result of these tests our species tree was constructed with the asserted Gracilicute / Terrabacteria bacterial root, total Oxyphotobacteria calibration, and Opimoda / Diphoda eukaryotic root.

Key node sensitivity tests

We further explored setting the ages of key nodes to specific values or equivalences, in order to evaluate the impact on gene duplication dates and the relative timing of duplications on the archaeal and mitochondrial branches. These results are tabulated in **Supplementary Table 5** and were set up similarly to the above.

- Setting LACA=LBCA results in LACA/LBCAs of intermediate age (3.23 Ga) with the effect of producing moderately younger ages for FECA (nFECA 2.92 to 2.70 Ga, mFECA 2.24 to 2.16 Ga) and LECA ages (1.74 to 1.67 Ga), however nFECA is still proportionately older than mFECA (0.54 versus 0.68 Ga in the species tree).
- Setting LBCA older (=> 4.00Ga), consistent with other analyses, does not greatly change FECA (nFECA 2.80 Ga, mFECA 2.16 Ga) or LECA (1.69 Ga) node values.

As a result of these tests we infer that changing the values of nodes deep within the tree has only modest effects on the eukaryogenesis timeline, and nFECA is found robustly older than mFECA. To explore whether our model of eukaryogenesis is robust when FECA branches are of comparable length, we set FECAs to equivalent age, this test;

- produces an intermediate age for these nodes (2.53 Ga)
- LECA remains at a similar value (1.72 Ga vs 1.74 Ga of the final tree)

To investigate the effect of the placement of eukaryotes within the Asgard archaea, we set nFECA sister to Hodarchaeales[3]. This test produces a modest decrease in both nFECA and mFECA ages (2.92 to 2.65 Ga; 2.30 to 2.23 Ga), but the two FECA branches remain different lengths, with non overlapping CIs on their node age, in this analysis LECA remains unchanged.

Supplementary Notes 4 - Species tree calibration information

This section concerns the fossil calibrations applied to the species tree, and the derivation of priors from the time-resolved tree applied to the single gene trees.

Fossil calibrations

Calibrations from fossil evidence are summarised in **Supplementary Table 6**, the placement of these calibrations and a literature justification of these calibrations is provided in the **Supplementary Data,** `Fossil_calibrations.docx`.

Speciation node age calibrations from the species tree

We created a script to produce new priors for application to single gene trees from the MCMCTree sampled node ages (`MCMC_to_calibrations.sh`). For each speciation node two types of calibration are produced, the first is a `MCMCTree` compatible skew-t distribution, which takes the naming convention t_n<species tree node number>. The second is a 95% confidence interval for the maximum age for the descendent clade, taking the naming convention cl_n<species tree node number>. These calibrations are seen on the annotated trees in the DA pipeline reports. Calibrations were derived from the sampled ages using the R library sn. Further explanation of the script is given in the `readme` in the same folder.

Additional derived calibrations

We asserted some additional calibrations for key nodes derived from the species tree.

- Calibrations specifying LACA, LBCA, LECA and LUCA nodes.
- Calibrations which give a maximal age constraint for clades of Archaea, Asgard archaea, Bacteria and Alphaproteobacteria.
- Calibrations which do not have corresponding to nodes on our species tree, but which have constraints from the LECA and FECA nodes, namely duplication nodes of Asgard archaeal and alphaproteobacterial descent.

These are listed and further defined in the **Supplementary Data** in `asserted_calibrations.txt`.

Supplementary Discussion

Supplementary Discussion 1 - Extended commentary on specific systems

Additional biological systems figures.

We include here additional figures (**Supplementary Figures 2-6**) relating to specific biological systems. **Supplementary Figures 2**,**3** expand on **Fig. 2** to show gene families involved in nuclear processes. **Supplementary Figures 4-6** cover processes not highlighted in our eukaryogenesis-defining features, and cover DNA repair, the ubiquitin system, and the ribosome and translation respectively. The figure formats are the same as the manuscript main figures.

Nucleus

DNA Replication

We identified duplications in the origin of replication complex (ORC), the helicase complexes recruited to them, and the machinery involved in the production of new genetic strands. Some of these are ancestrally homomeric complexes which have fully heteromerised (MCM [4], GINS [5]), whilst the DNA polymerases have become functionally specialised. With one exception their duplications begin before mitochondrial endosymbiosis. Several of these complexes are closely interacting physically, (GINS, MCM and RFC [6]) and their duplications overlap (that is, the 95% CIs of their ages overlap substantially, GINS 2.91-2.38 Ga; MCM 2.88-2.21 Ga; RFC 2.88-2.28 Ga, **Supplementary Figure 2A**). We hypothesize that this could be an example of runaway compensatory cascade occurring between interacting protein complexes.

The replicative DNA polymerases belong to discrete but subfunctionalised complexes, and they and their cofactors diverge and complete their complexification before 2.10 Ga (POLE/D diverge 2.73-2.36,Ga, divergence of POLA 2.50-2.08 Ga **Supplementary Figure 2A**). The replicative DNA polymerases function in the same compartment although with different specialisations and processivity in the replication fork.

These results indicate that a modern-type eukaryotic DNA replication machinery was likely present before mitochondrial endosymbiosis, with differentiated polymerases and fully heteromeric MCM helicases. In eukaryotes, these heteromerised complexes are able to integrate multiple inputs such as cell cycle checkpoint control [7], as a result of unique protein-protein interactions of the diverged paralogs.

Transcription

In contrast to prokaryotes, eukaryotes possess multiple RNA polymerases, specialised in producing different kinds of transcripts. The ancestral archaeal RNA polymerase is a multimeric protein complex and functionally closest to eukaryotic RNA polymerase II, and is somewhat more elaborated than the bacterial polymerase belonging to a transcription

system with more basal transcription factors, promoter elements as well as histone-bound DNA [8].

Duplications of genes in the ancestral RNA polymerase complex occur between 2.90-1.90 Ga, **Supplementary Figure 2B**. However unlike some of the previously described complexes they still share common subunits suggesting that full heteromerisation proceeds to the extent of supporting new functionalization [9]. In our analysis, RNA polymerase III diverges first (2.73-2.32 Ga, mirrored in the divergence of its transcription factor; TFIIIB 2.90-2.16 Ga; green circles **Supplementary Figure 2B**), the divergence of RNA polymerase I occurs later (2.57-1.12 Ga, green diamonds **Supplementary Figure 2B**). Complexification of the transcription machinery starts early, but completes around mitochondrial endosymbiosis with differentiated polymerases, more complex regulatory elements (more general transcription factors), and other levels of co-transcriptional control.

The differentiated polymerases control the production of largely discrete pools of transcripts, RNA Polymerase I; rRNAs - ribosomes, RNA Polymerase II; mRNAs, snRNAs, and miRNAs - proteins and their regulation of expression, RNA Polymerase III; tRNAs, 5S rRNA, U6 snRNA - translation and other essential functions. Specialised polymerases as a consequence could exert an effect on genomic architecture and organisation, as genes driven by the same polymerase may be clustered for efficiency. This would be especially true for RNA polymerase I, and could drive the development of subnuclear regions for processing specific types of transcript such as the nucleolus.

Splicing and RNA processing

The eukaryotic cell contains specialised compartments and complexes for the processing of introns. Eukaryotic introns and portions of the spliceosomal machinery are hypothesized to descend from prokaryotic type II introns [10]. However it is possible that these introns were present in both FECAs [11,12]. At some point these self splicing elements were functionally replaced by snRNAs and the spliceosomal ribonucleoprotein complexes[10]. In eukaryotes the major spliceosome comprises several snRNAs, proteins, and a heteroheptameric protein complex called Sm (major Spliceosome). Sm is related to another RNA processing complex, LSm (Like-Sm), and it seems likely these complexes arose by duplication from a single homomeric complex in an archaeal ancestor [13]. Previous studies have suggested the LSm as the ancestral complex [10] due to the greater divergence of Sm paralogs, the specific specialisation of the Sm spliceosome, and the wider functional role of the LSm complexes.

Our analysis suggests that the initial duplication of this archaeal protein occurred before nFECA, and heteromerised early along the eukaryotic stem (>2.10 Ga **Supplementary Figure 3A**). This heteromeric complex then underwent a duplication [14] leading to the production of paralog pairs. We resolve this in the period 2.91-1.85 Ga (green diamonds **Supplementary Figure 3A**).

The duplication of the Sm/LSm has led to complete heteromerisation, fitting with the role of these complexes as platforms to bind different populations of RNA processing factors. The Sm/LSm complex duplication history also provides insights into the state of the eukaryotic cell beyond splicing, as the biogenesis of these complexes and their association with their snRNAs differ.

The SM ring stably binds its snRNAs and is imported into the nucleus as a large cytosolically assembled ribonucleoprotein complex (requiring nuclear pores in extant eukaryotes). Variants of the LSm complexes are differently localised and perform different roles in the cytosol (LSm1-8) and nucleus (LSm2-7). The nuclear LSm2-7 complex only

transiently binds the U6 snRNA and relies on a different assembly mechanism where the U6 snRNA is transcribed by RNA pol III (in contrast to the other snRNAs), this alternate processing prevents nuclear export. This is then an adaptation (to the emergence of the nuclear compartment) to retain the nuclear localisation of U6 (as it would not be imported with the LSm complex from the cytosol). For this to evolve it would necessitate the functional divergence of RNA pol III (green circles **Supplementary Figure 2B**) before the emergence of the nuclear compartment, and the neofunctionalisation of LSm.

For Sm and its stably bound snRNAs, no RNA pol III transcription was required to maintain function, and cytosolic assembly provides a mechanism for quality control on spliceosomal complexes (and their snRNAs) before they are imported to produce mRNAs. For both complexes, nuclear localisation signals (NLS) are required for import through nuclear pores. LSM2 and LSM8 and their Sm paralog pairs SMB, SMD1 contain the nuclear localisation signals for their respective complexes, and suggest the nuclear localisation signal was included before complex duplication, and possibly before full heteromerisation of the ancestral complex.

Later duplications in the LSm NLS containing components producing LSMs1/10 resulted in the cytosolically localised LSM1-8 complex. Duplication here has not resulted in further heteromerisation between cytosolic and nuclear LSm complexes before LECA, and has served to differently localise a complex to different compartments.

Histones

Histones which make up the principal component of eukaryotic chromatin have analogous families in prokaryotes. In eukaryotes, chromosomal DNA is wound around an heteromeric octamer core. In our analysis we were able to time resolve 3 of the 4 histone families and resolve separation of H2A/H2B/H3 before 2.10 Ga (**Supplementary Figure 3B**). We find the centromere specific histone protein CENP-A appears to have diverged after LECA within the H3 histone group **Supplementary Data** (- this is visualised in the domain analysis report for the histones).

Chromatin

Histones likely had regulatory roles in the archaeal ancestor [15], but acquired new modes of regulatory control during eukaryogenesis, including the emergence of N-terminal tails capable of being post-translationally modified, laying the foundation for eukaryotic chromatin-based epigenetic regulation. In our analysis, we resolve three families of histone modifying enzymes that underwent pre-LECA gene duplications, two of alphaproteobacterial origin (the METTL family, dated to 2.24–1.85 Ga, and SBNO1/2, 2.07–1.81 Ga), and one of inferred Asgard archaeal origin (the HDAC family, 2.26–1.98 Ga). The timing of these duplications are contemporary with or follow mitochondrial endosymbiosis suggesting that the elaboration of eukaryotic chromatin regulation was a relatively late event in the eukaryogenesis timeline and involved gene families from the mitochondrial endosymbiont.

SMC family proteins

At an organisational level above histones, cohesins and condensins play a role in organising chromosome structure during cell division, and belong to the larger Structural Maintenance of Chromosomes (SMC) family, for which we resolve duplications early in our eukaryogenesis timeline (**Supplementary Figure 3C**). In modern eukaryotes, these proteins form heterodimers of discrete function [16]. In our results, an initial divergence of the ancestor

of SMCs1/4 from the ancestor of SMCs2/3 occurs between 2.84-2.55 Ga. A second round of duplications produce the two modern functionally discrete heterodimers; SMC2/4, condensin; and SMC1/3, cohesin (green triangles, 2.64-2.12 Ga, **Supplementary Figure 3C**). The SMC5/6 heterodimer seems to have a separate archaeal origin [17], but undergoes duplication in the same period (2.87-2.23 Ga). In modern eukaryotes, factors associating with the condensins give rise to functionalised condensin complexes but these appear to be post LECA complexifications as there is no consistent essentiality or functionalisation across all eukaryotes [17]. Based on the function of characterised SMC complexes in prokaryotes [18], the ancestral complex likely had condensin-like function (that is, condensing chromosomes). Sister chromatid cohesion in cohesins appears to be a eukaryotic innovation, and from the role of cohesin in spindle assembly, meiosis and homologous recombination repair, suggest an adaptation to eukaryotic genome architecture.

Nuclear import and export

We determine the importins and the Ran GTP exchange/activation factors to be eukaryote-specific proteins. We find Ran to be of archaeal origin but not dateable by our method. The GPN-loop GTPases GPN1/2/3 are necessary for the assembly and import of cytosolically produced RNA Polymerase II into the nucleus [19], duplications in this family occur between 2.81-1.99 Ga (**Supplementary Figure 3C**). mRNA export from the nucleus is mediated by the transcription and export (TREX) complexes; one component, the PCI domain containing PCID2 of the TREX-2 complex, diverges at 2.78-2.23 Ga.

Subnuclear structures

The eukaryotic nucleus is further subdivided into functionally distinct subnuclear structures. The nucleolus is a specialised nuclear region responsible for the maturation of small RNAs and associated with the telomeres. Evidence for subnuclear structures are suggested by the divergence of RNA binding proteins which are exchanged on maturation in the nucleolus for nucleolar specific paralogs (GAR1/NAF1, 2.69-2.01 Ga), and proteins of ribosomal origin, which have a nucleolar paralog in extant eukaryotes (IMP3 2.90-2.40 Ga, RSL1D1 2.83-2.09 Ga, RSL24D1 2.75-2.08 Ga, (**Supplementary Figure 3**). Protein components essential to the structure of the nucleolus have been identified in archaea opening the possibility of a primordial functionalised compartment which later fully developed to its complete state by LECA.

Exosome complex

The RNA degrading exosome complex is composed of a limited number of gene families, which are partially heteromerised complexes in extant prokaryotes and undergo complete heteromerisation in the eukaryotic stem before mitochondrial endosymbiosis (**Supplementary Figure 3D**). Interestingly the RNAse activity of the original archaeal components was later replaced by genes of bacterial origin which were added after the complex had become completely heteromerised.

DNA repair

Modern eukaryotes have nuclear DNA repair machinery of both bacterial and archaeal origin (**Supplementary Figure 4**). The stem eukaryote would have had a complete archaeal

system for DNA repair at nFECA, with the implication that bacterial components would have been integrated during eukaryogenesis. We suppose that DNA occupies a limited number of intermediate repair states which would have also been found in both archaeal and bacterial systems. Considered in this sense, integrating additional DNA repair enzymes would be no different to substituting or complementing enzymes on a metabolic pathway. Some specific repair mechanisms are of mixed origin with dateable duplications including the mismatch, non-homologous end joining and homologous recombination pathways. We observe complex heteromerisation in the bacterial MutS/L (2.18-1.79 Ga), and Ku complexes (2.10-1.90 Ga), following the theme of heteromerisation observed for archaeal complexes. In contrast to other nuclear processes, the archaeal duplications involved in DNA repair occur across the entirety of the eukaryogenesis timeline.

Meiosis

Several enzymes of archaeal (DMC1, 2.55-1.98 Ga; EME1/2, MUS81, 2.45-1.92 Ga) and bacterial (MSH4/5, 2.03-1.87 Ga) origin have specialised paralogs participating in meiosis and arise from duplications after mitochondrial endosymbiosis, suggesting that the modern meiotic machinery is a later invention (**Supplementary Figure 4**).

Membrane biology

Prenylation

Prenylation is another important post-translational modification, and particularly important in the activation of cellular GTPases. The complexes that perform these modifications are heterodimeric and have duplicated to functionally discrete complexes which prenylate different GTPase families (**Fig. 2**). From our time resolved expansion of the Rab family we would expect early duplication, as well as synchronous duplication of the heterodimeric complex components, which we observe.

Protein turnover and biogenesis

Proteasome

Proteasomes are large protein complexes and although not homomeric in their pre-eukaryotic state, they are composed of multimers from a small number of gene families. The proteasomal 20S core particle is entirely heteromerised in eukaryotes. Its complexification began early and was likely completed before mitochondrial endosymbiosis, with the gene families of the alpha and beta rings, 20S core particle and base subunits of the 19S regulatory particle duplicating over the same time frame (**Supplementary Figure 5A**). Because of close physical interaction between the proteasome rings, the heteromerisation of this complex could be part of a compensatory cascade.

The proteasome is further elaborated in eukaryotes by the addition of a regulatory 19S particle. Some of these subunits appear to be eukaryotic innovations, others contain the PCI domain, named for its occurrence in the Proteasome, CSN and EIF3 (PCI). PCI protein complexes serve as platforms for modifying proteins. For the proteasome and CSN this means the removal of ubiquitin family proteins, specifically the removal of ubiquitin and the ubiquitin like protein NEDD respectively. The existence of paralog pairs points to an ancestral complex that has become duplicated: one which heteromerised early and

duplicated around the time of the mitochondrial endosymbiosis (green circles **Supplementary Figure 5A**). From our limited data it is hard to say which PCI complex related function emerged first (**Supplementary Figure 5B)**.

Ubiquitin

Labelling of protein substrates with ubiquitin or ubiquitin-like proteins is a three stage process, and we have time-resolved the enzyme families that perform the first two stages; activation and conjugation. We observe progressive elaboration of this system throughout eukaryogenesis (**Supplementary Figure 5B**). Strikingly, we observe a relatively late and synchronous emergence of the activating (SUMO1/NAE1 2.25-1.93 Ga) and conjugating enzymes (UBE2E1/2/3 2.44-1.78 Ga) for their target ubiquitin-like proteins NEDD8 and SUMO (green pentagons, **Supplementary Figure 5B**).

Most SUMO targets in extant eukaryotes are nuclear and involved in transcription regulation, DNA repair, and chromatin organisation [20]. This functionalisation of SUMO might require the pre-existence of the nucleus and eukaryotic transcription control. The late emergence of NEDD8 fits with our estimated origin for the deneddylating CSN complex at around 2.2 Ga.

Translation

Compared to archaea, eukaryotic translation has many of the same components, but is more elaborately regulated. In our results we find duplications in the protein components of the ribosome as well as its initiation and elongation factors and complexes involved in post-translational modification (**Supplementary Figure 6**). The 43S preinitiation complex is a large ribonucleoprotein complex which itself is composed of multimeric subunits in which we have detected multiple paralog pairs with a range of duplications ages spanning 2.90-1.87 Ga . The largest subunit EIF3 contains PCI domain proteins also found in the proteasome and CSN where its paralogs perform a similar role as a protein binding scaffold.

Supplementary Figures

Supplementary Figure 1. Time-resolved species tree.

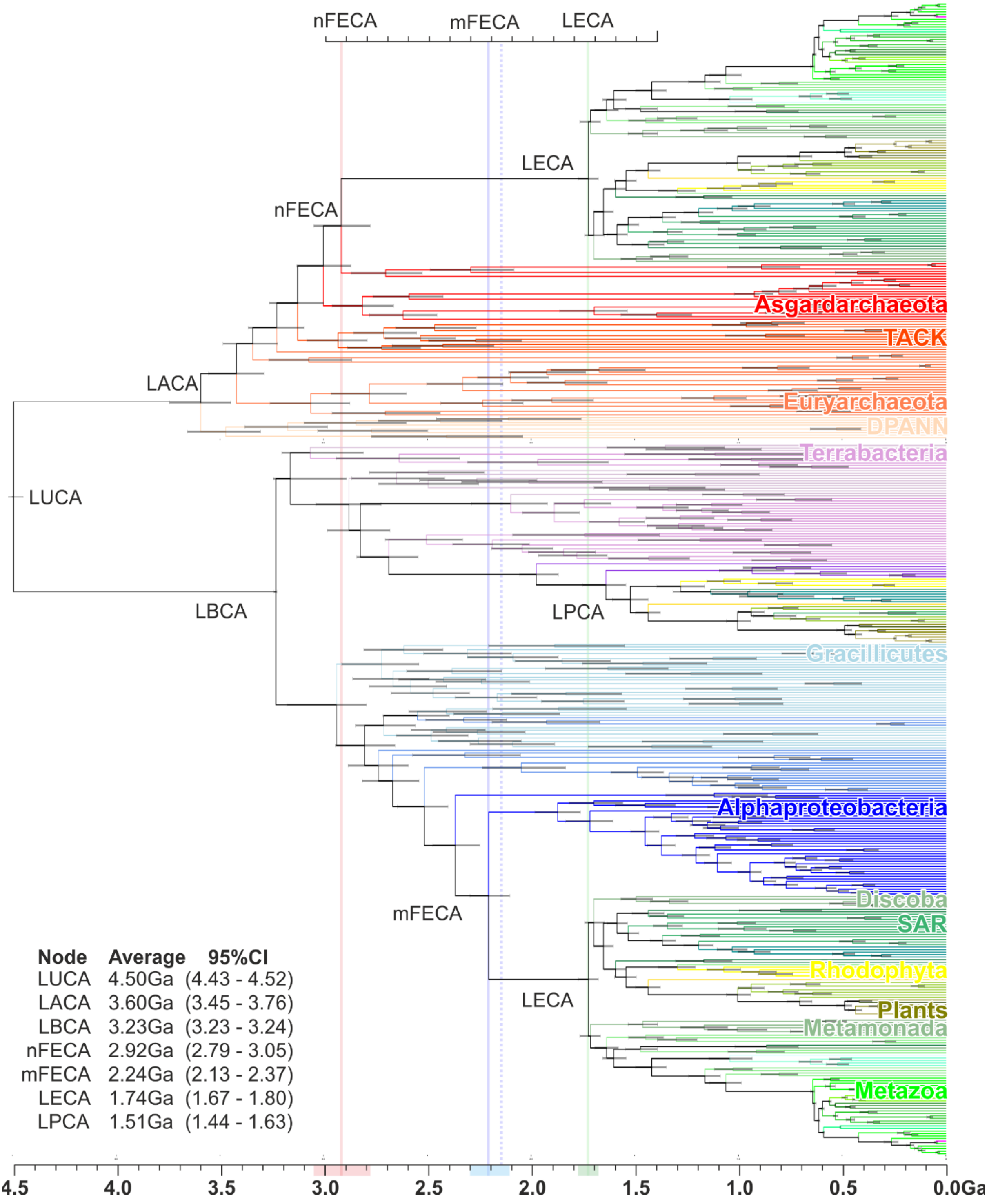

This expansion of the time-resolved species tree in **Fig. 1A** shows our determined ages for species groups (shown to the right) the x-axis is given in units of billions of years before the present. To give a sense of the scale of the eukaryogenesis timeline, the positions of the FECAs and LECA are indicated in vertical coloured lines. In this study, the cross-bracing approach allows us to leverage the information of speciation nodes and transmit rate information over LUCA (i,e. gene families in eukaryotes which paralogs of separate asgard archaeal and alphaproteobacterial descent) and LBCA nodes (gene families with paralogs from plastid and mitochondrial descent), as a result duplicate LECA nodes as seen with identical ages. Node bars indicate the 95% C.I. from pooled MCMCTree analyses.

Supplementary Figure 2. Nuclear processes- DNA replication and transcription.

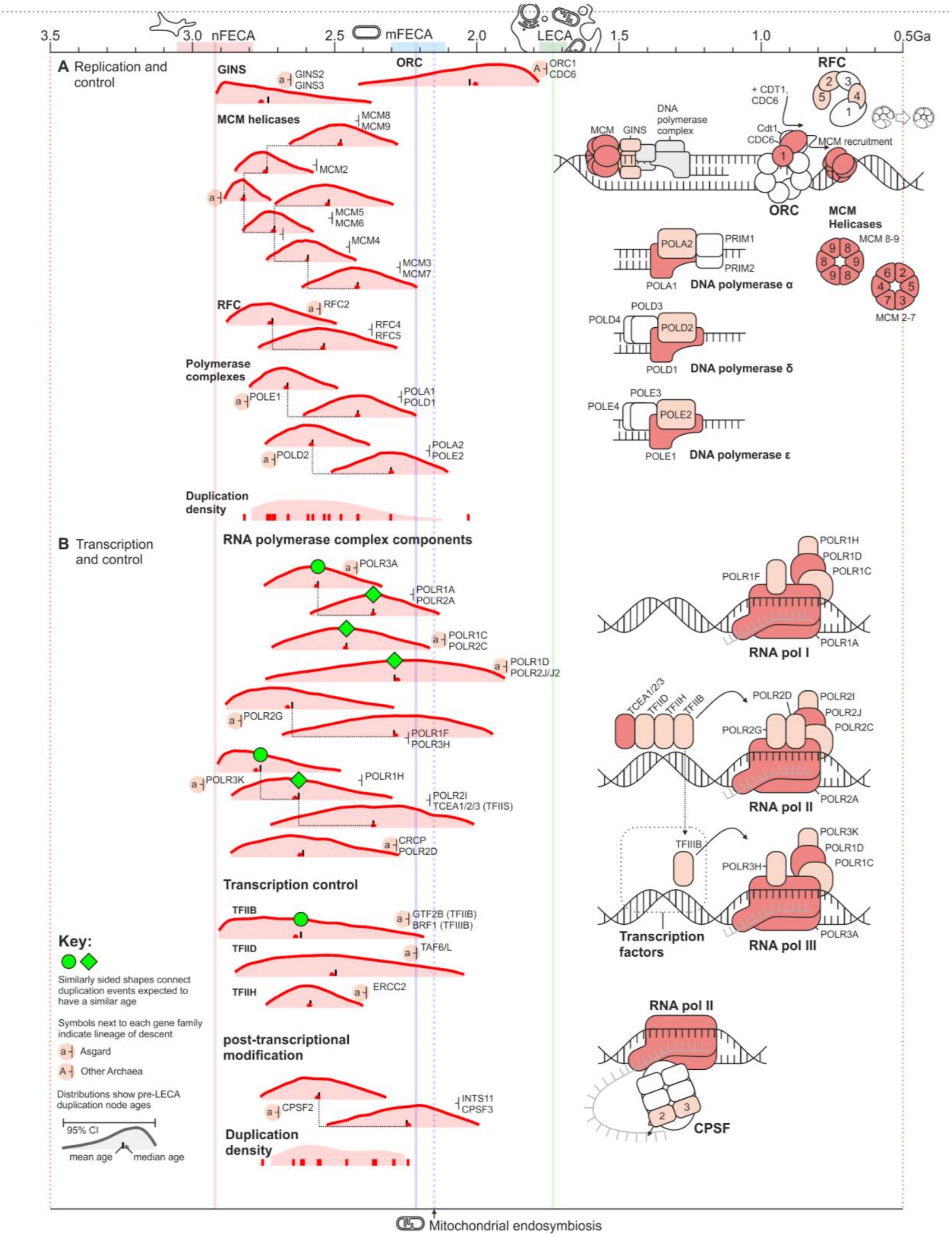

This figure expands on some gene families shown in Fig. 2 to show all the duplications we have determined for the DNA replication and transcription machinery. All of our pre-LECA duplicating gene families in this figure are of archaeal descent, and we have further suggested than the duplication of the RNA polymerase complexes may create a pattern of synchronous duplications. Each pre-LECA duplication node is shown as probability distribution of the node age, a description of which is expanded upon in the figure Key.

Supplementary Figure 3. Nuclear processes- splicing, RNA

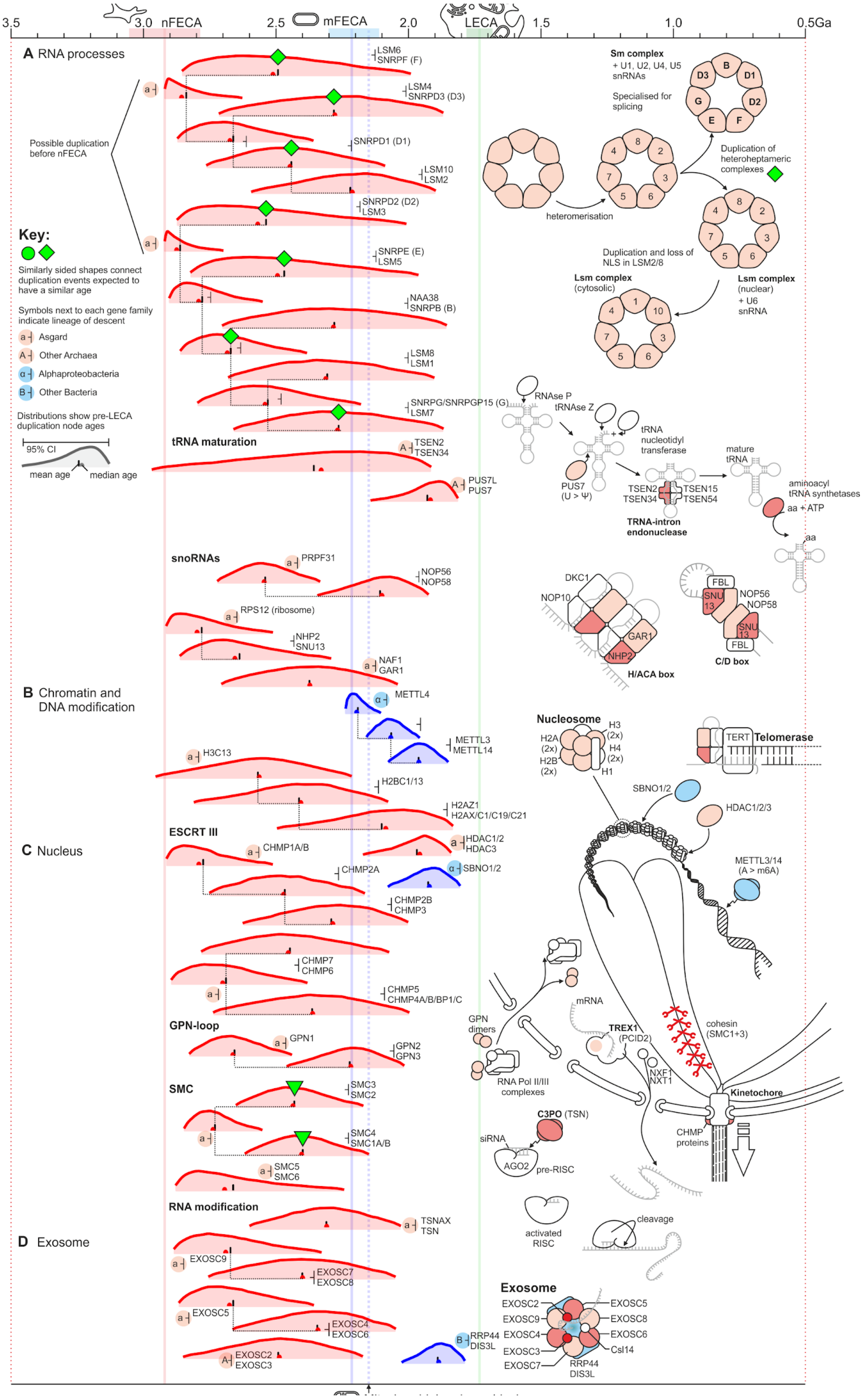

In this figure we expand on Fig. 2 by showing the pre-LECA duplications of the spliceosomal complex with respect to other nuclear localised RNA processing pathways as well show gene families involved in genomic organisation. In this figure pre-LECA duplication nodes are shown as age distributions, coloured by gene family origin. Each age distribution shows the 95% CI of the node age, and shows the mean and median node age (black tick, red dot respectively).

Supplementary Figure 4. DNA repair system.

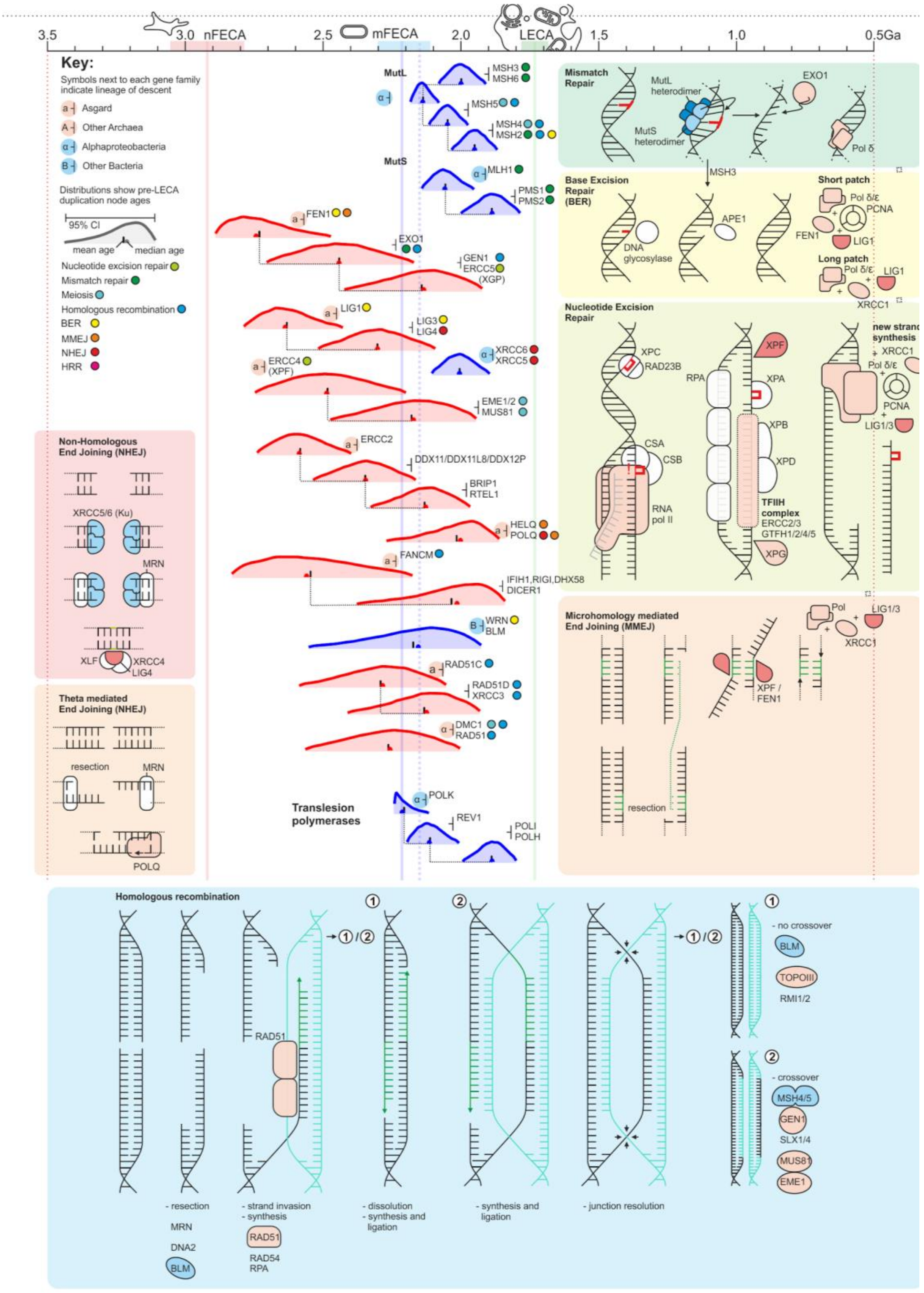

Prokaryotes possess a variety of DNA repair pathways, and eukaryotes have inherited a mosaic of these pathways. Interestingly, eukaryotic pathways can contain components from different prokaryotic families, perhaps because the intermediate pathway states are process-interchangeable states of DNA. The figure Key describes the format of node age distributions, as well the origin of each gene family, further the function of each gene is colour coded to the schematic repair pathways around the periphery of the diagram.

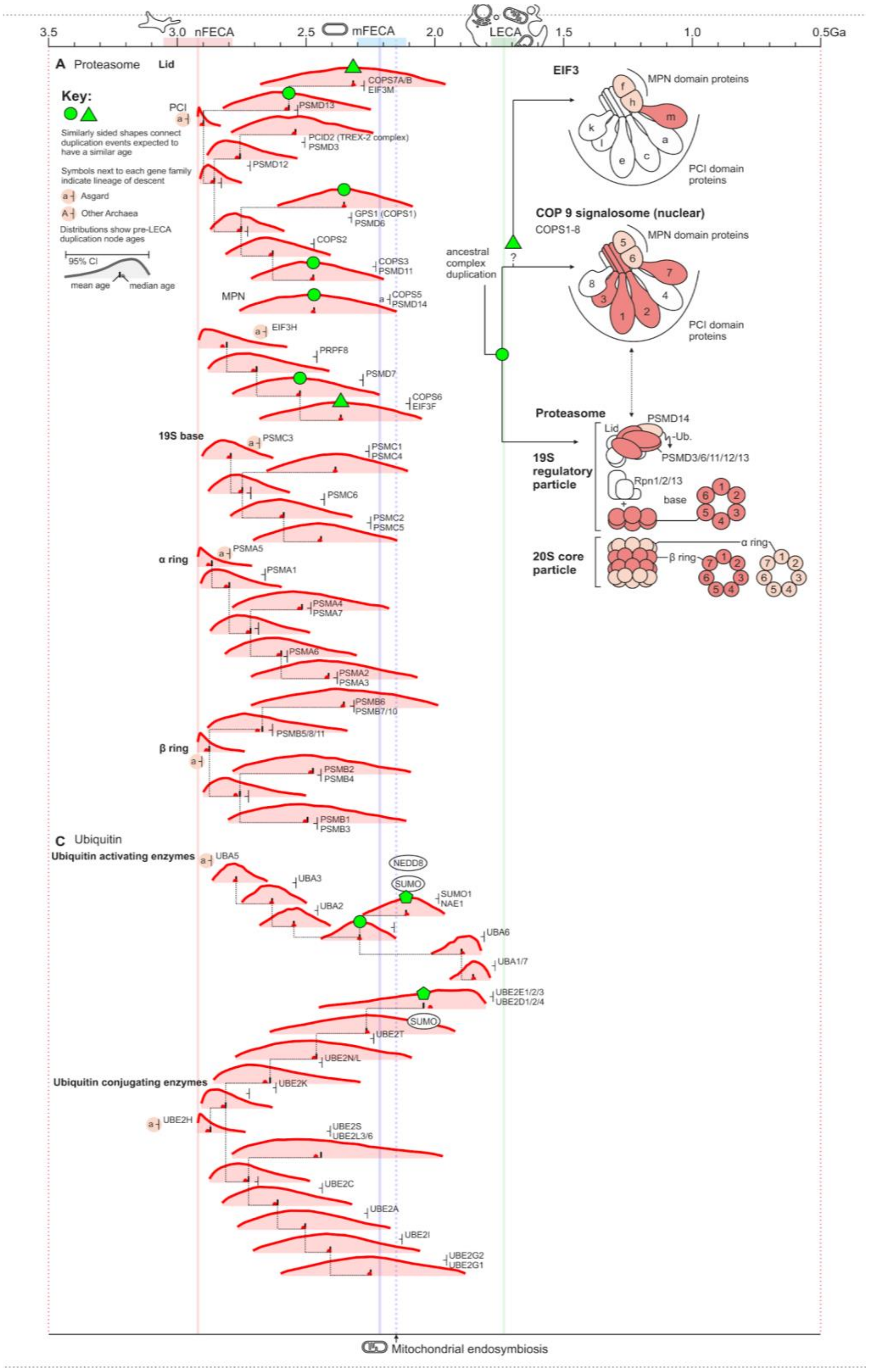

Eukaryotes possess supramolecular complexes for protein turnover and degradation, and this figure shows the pre-LECA duplications associated with the archaeal-descended protein complexes of the proteasome (**A**) and ubiquitin system (**B**). Subunits within these protein complexes are in close arrangement, which we suggest might drive the early and coincident heteromerisation of the proteasome, despite being composed of multiple separate protein families. Duplication of this COP9 / proteasome regulatory complex produced a number of duplication events, which we observe have overlapping duplication dates (green circles). This process might also be mirrored by the development of the principally nuclear localised ubiquitin-like protein SUMO and its activating proteins, **B**.

Supplementary Figure 6. Ribosome and translation system.

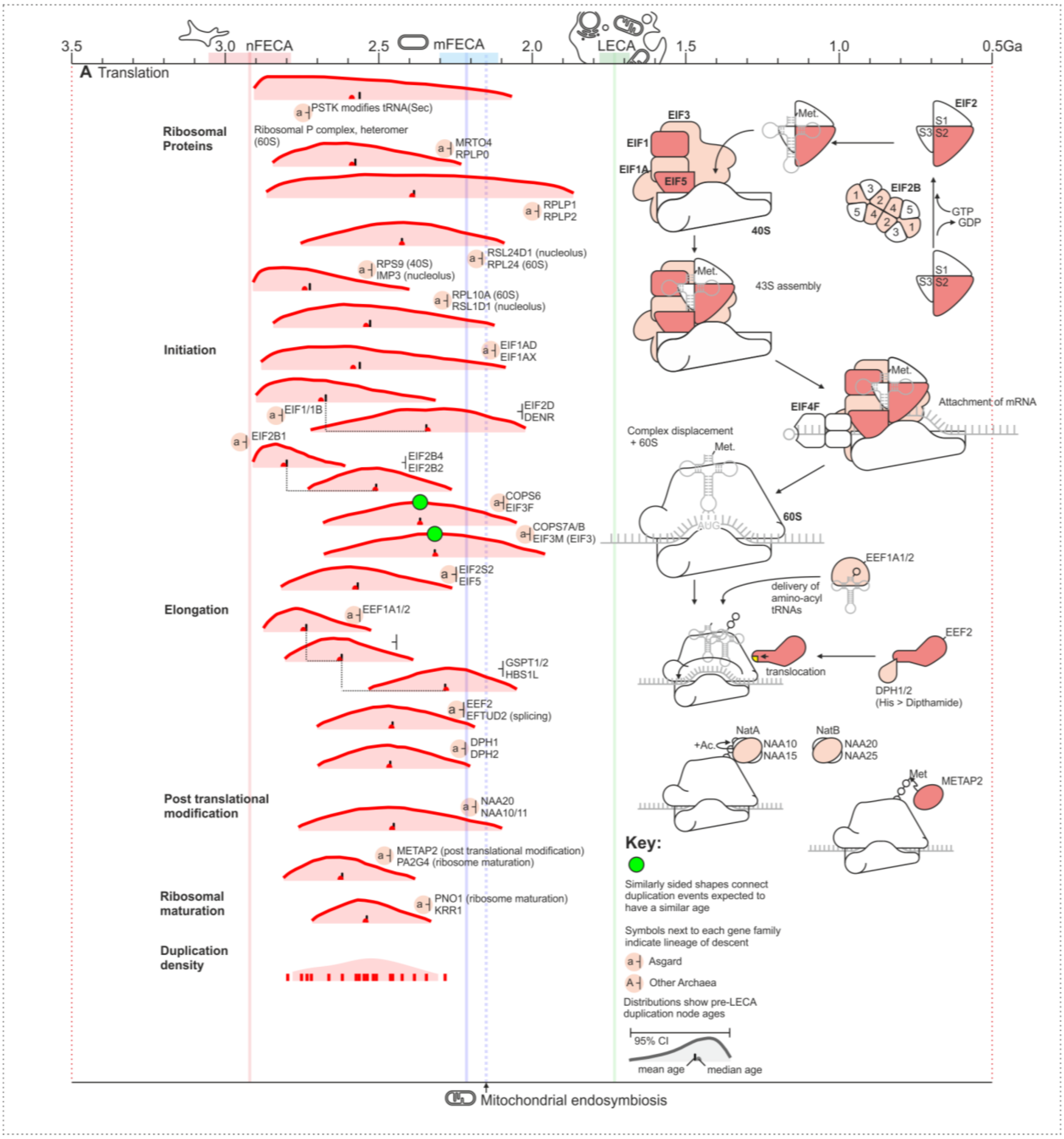

In this figure the ages of pre-LECA duplications are shown for gene families involved in the ribosome and translation system, the figure key explains the format of the duplication node age distributions, and origin of the depicted gene families. Whilst many of the distributions have broad confidence intervals, the majority are before our proposed age for the mitochondrial endosymbiosis.

Supplementary Figure 7. Flow diagram of the Domain Origins (DO) pipeline.

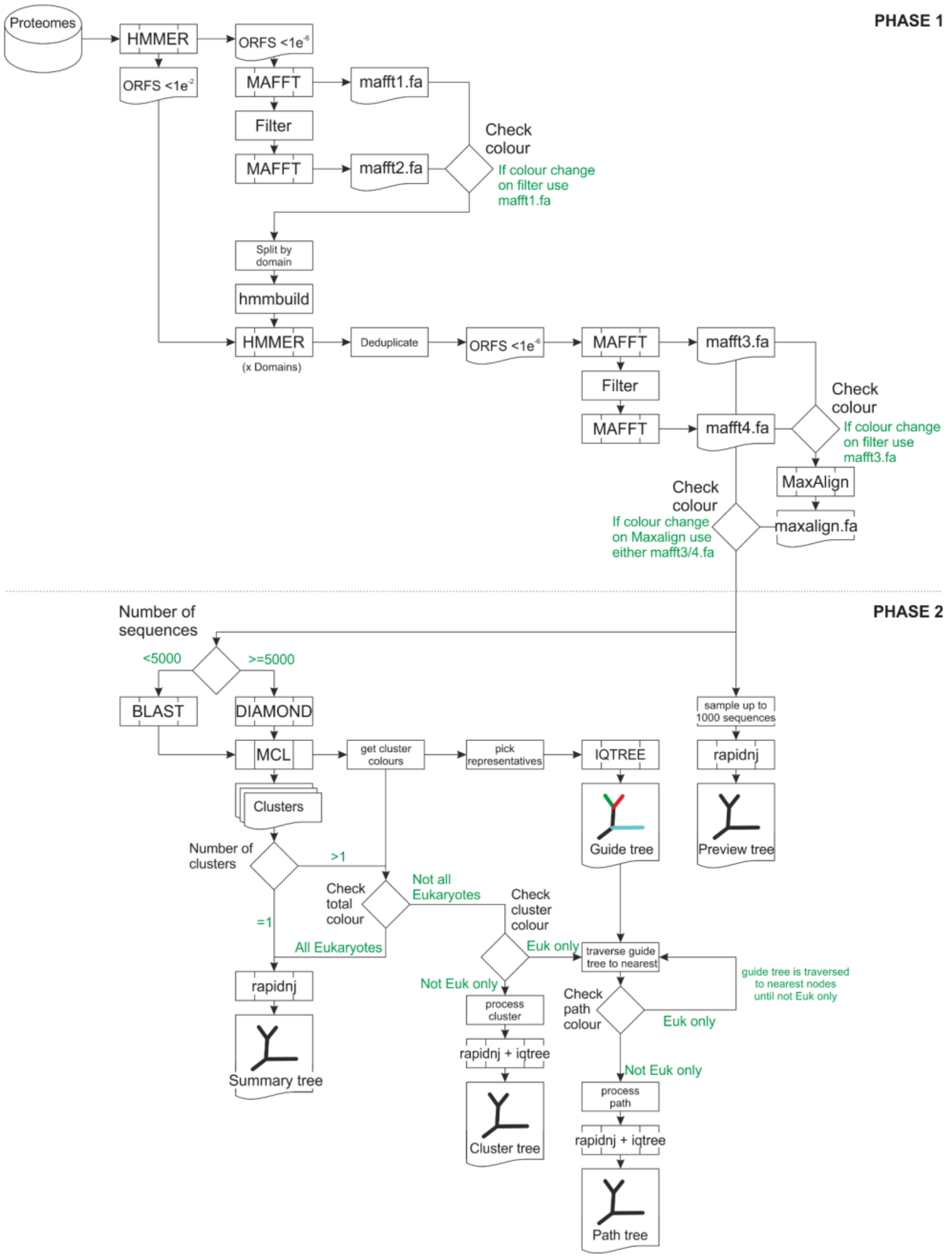

Flow diagram of the Domain Origins (DO) pipeline. The pipeline consists of two phases: a search phase and a clustering and tree traversal phase. In phase one, a HMM search is performed, high-scoring results are aligned, filtered, and re-aligned to produce new HMMs for each supergroup (Eukaryotes, Bacteria, Archaea). The search is then iterated over the original search results. Taxonomy diversity checks are conducted throughout filtering and alignment optimization steps, and alignment statistics are calculated to select between alignments for further analysis. The second phase involves sequence clustering and tree construction. Depending on the number of clusters, trees are created for whole or cluster-informed portions of the total sequence pool.

Supplementary Figure 8. Colour system used in the DO pipeline.

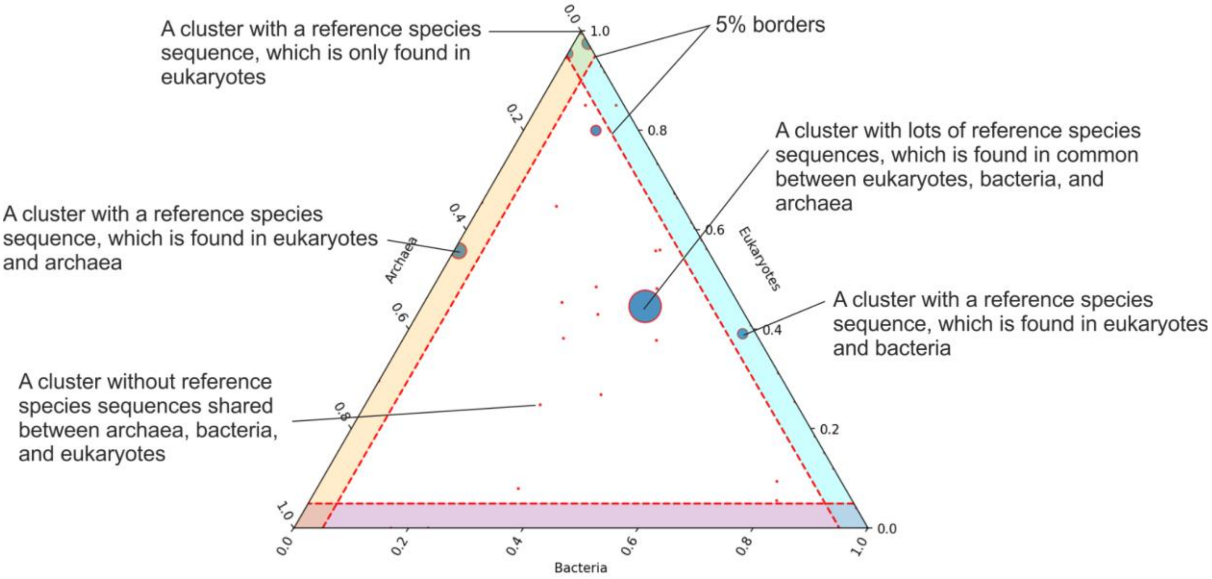

Markov Cluster Algorithm (MCL) clustering is used to break down intricate gene families into sequence clusters. These clusters are then mapped within a ternary color space. The axes of this space correspond to Archaea (RED), Bacteria (BLUE), and Eukaryotes (GREEN). Clusters dominated by sequences from a single supergroup are depicted in primary colors. Secondary colors are assigned to clusters containing sequences from multiple supergroups. Red dots represent clusters that lack reference species sequences, circles indicate clusters with reference species sequences, and the size of these circles reflects the sequence count.

Supplementary Figure 9. HMM search figure from the DO pipeline.

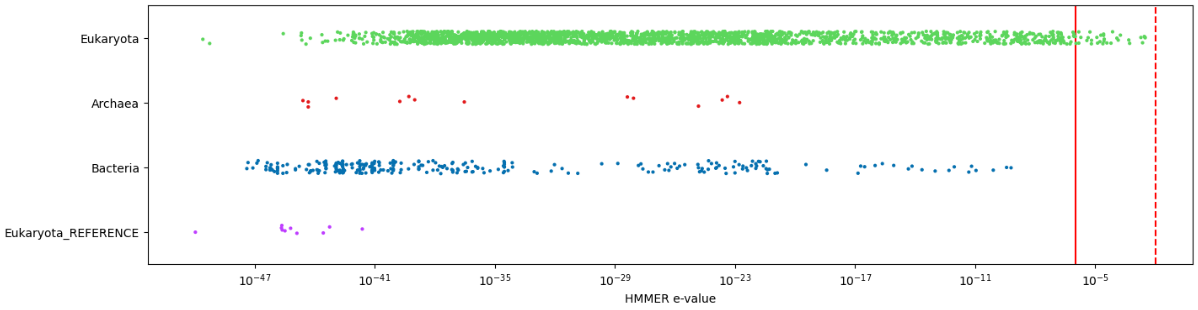

HMM search sequence hits are shown sorted by their e-value on the x axis, and sorted by track by their clade of origin (including a track for hits against a reference species), the filter boundaries for strong and weak results are indicated by solid and dashed red lines respectively.

Supplementary Figure 10. Alignment optimization figure from the DO pipeline.

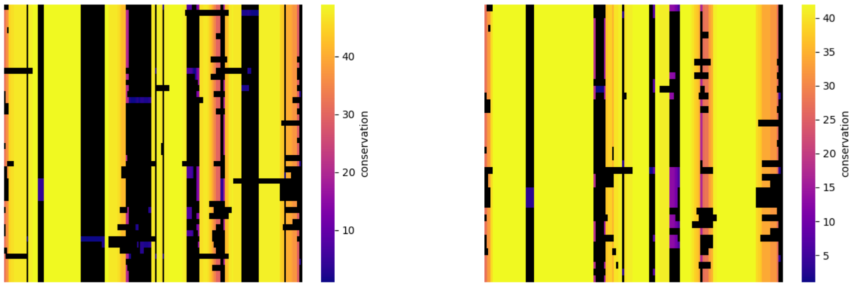

Visual comparison of sequence alignments before and after coarse filtering. Left panel: Original MAFFT alignment. Right panel: Alignment after coarse filtering. Each row represents a protein sequence, and each column represents an aligned position. Colour intensity within a column indicates the frequency of amino acid occupancy at that position, with black regions denoting gaps. In this example filtering has improved the protein sequence alignment by reducing gaps and increasing occupancy.

Supplementary Figure 11. Guide tree traversal in the DO pipeline.

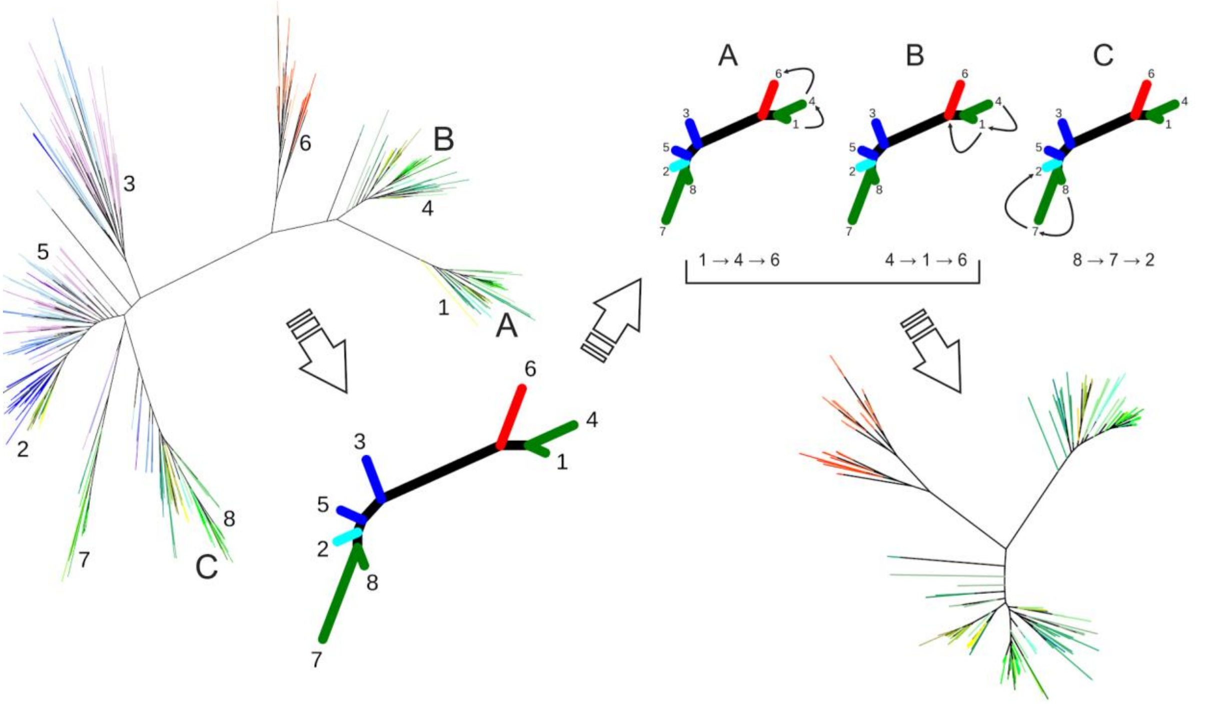

In the guide tree traversal process, first an all-versus-all DIAMOND or BLAST of sequences if performed, next the pairwise alignment scores are used for MCL clustering. A representative sequence is selected from each cluster to generate a guide tree. For each reference protein in this example (A, B, C), the tree is traversed until a non-exclusively eukaryotic group is found. These cluster traversal ‘paths’ allow for the subsampling of the total protein sequence pool, resulting in sub-trees with a narrowed evolutionary focus.

Supplementary Figure 12. Flow diagram of the Domain Analysis (DA) pipeline.

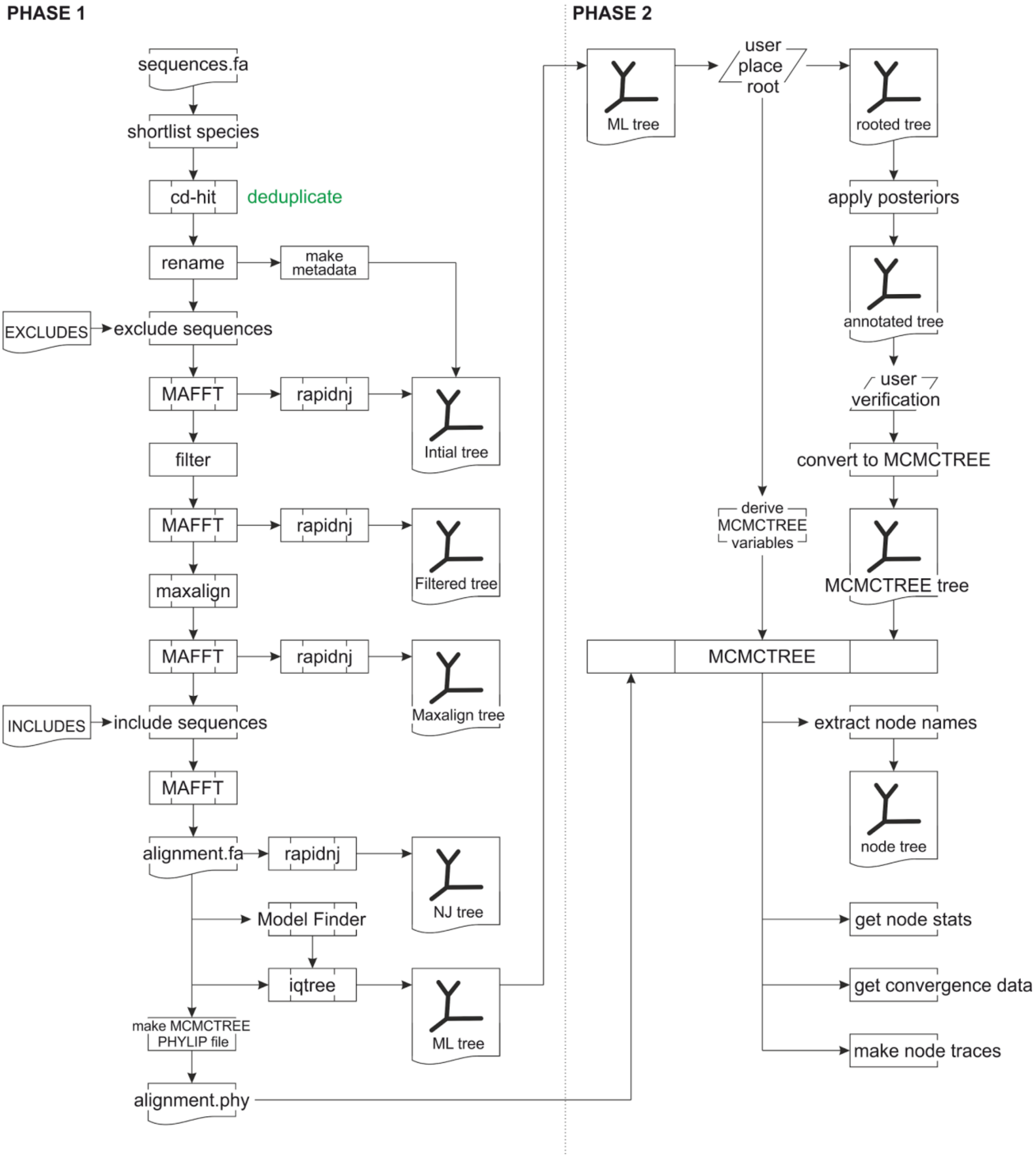

The domain analysis pipeline is designed to take protein sequences identified in the DO pipeline. In the first stage the pipeline attempts to build an optimised alignment and maximum likelihood tree, this can be iterated with user input highlighting sequences to keep or reject. In phase two, the maximum likelihood tree is then reconciled to the species tree and node priors applied before running MCMCTree. After time resolution the pipeline produces a report on the node ages and convergence of the MCMCTree run.

Supplementary Tables

Supplementary Table 1. Software used in this study.

Program	Version	Command	Reference
BMGE	v1.12	`bmge`	[21]
BLAST	v2.15.0+	`blastp`<br>`makeblastdb`	[22]
CD-HIT	v4.8.1	`cd-hit`	[23]
DIAMOND	v2.1.9.163	`diamond`	[24]
HMMER	v3.3.2	`hmmbuild`<br>`hmmsearch`	[25]
IQ-tree	v2.2.0.3	`iqtree`	[26]
MAFFT	v7.508	`fftnsi`<br>`fftns`<br>`linsi`	[27]
MaxAlign	v1.0	`Maxalign.pl`	[28]
MCL	v22-282	`mcl`	[29]
MCMCTree (PAML)	v4.10.0	`codeml`<br>`mcmctree`	[30,31]
RapidNJ	v2.3.2	`rapidnj`	[32]
TransDecoder	v5.7.1	`TransDecoder.LongOrfs`	https://github.com/TransDecoder/TransDecoder/blob/master/TransDecoder.LongOrfs
Treerecs	v1.2	`treerecs`	[33]

Supplementary Table 2. Source data, naming, and taxonomy information

This table provides the source information for the species used in this study. For each species the data source (NCBI or Eukprot) and whether a proteome was available (where proteomes were not available ORFs were predicted using `Transdecoder.LongOrfs`). Species names are according the NCBI taxonomy identifier, where multiple representatives of a species have been distinguished by a suffixed number. These naming conventions are found on the time resolved species tree and individual gene trees. Highlighted in the table (by *) are two Hodarchaeales as defined by GTDB, but which are labelled by NCBI as Heimdallarchaeota.

Accession	Data source	Available proteome	Species name	NCBI taxid	Supergroup	Subgroup	In time-resolved species tree
GCA 001940705	NCBI	Y	Thorarchaeota archaeon-1	1837170	Archaea	Asgard group	N
GCA 001940755	NCBI	Y	Heimdallarchaeota archaeon-1	1841596	Archaea	Asgard group	N
GCA 002825515	NCBI	N	Thorarchaeota archaeon-2	1969372	Archaea	Asgard group	N
GCA 002825535	NCBI	N	Thorarchaeota archaeon-3	1969371	Archaea	Asgard group	N
GCA 003662885	NCBI	Y	Heimdallarchaeota archaeon-2	2026747	Archaea	Asgard group	N
GCA 004376705	NCBI	Y	Lokiarchaeota archaeon-1	2053489	Archaea	Asgard group	N
GCA 004524425	NCBI	Y	Lokiarchaeota archaeon-2	2053489	Archaea	Asgard group	N
GCA 004524435	NCBI	Y	Thorarchaeota archaeon-4	2053491	Archaea	Asgard group	N
GCA 004524545	NCBI	Y	Lokiarchaeota archaeon-3	2053489	Archaea	Asgard group	N
GCA 004524595	NCBI	Y	Thorarchaeota archaeon-5	2053491	Archaea	Asgard group	N
GCA 004524725	NCBI	Y	Lokiarchaeota archaeon-4	2053489	Archaea	Asgard group	N
GCA 008000775	NCBI	Y	Prometheoarchaeum syntrophicum-1	2594042	Archaea	Asgard group	N
GCA 016839265	NCBI	N	Odinarchaeota archaeon	2053490	Archaea	Asgard group	Y
GCA 019056805	NCBI	N	Baldrarchaeota archaeon	2799005	Archaea	Asgard group	N
GCA 019058315	NCBI	N	Helarchaeota archaeon	2719382	Archaea	Asgard group	Y
GCA 020344955	NCBI	Y	Lokiarchaeota archaeon-5	2053489	Archaea	Asgard group	N
GCA 020348965	NCBI	Y	Heimdallarchaeota archaeon-3*	2026747	Archaea	Asgard group	N
GCA 020348985	NCBI	Y	Thorarchaeota archaeon-6	2053491	Archaea	Asgard group	N
GCA 020353515	NCBI	Y	Heimdallarchaeota archaeon-4*	2026747	Archaea	Asgard group	N
GCA 020355105	NCBI	Y	Thorarchaeota archaeon-7	2053491	Archaea	Asgard group	N
GCA 021498095	NCBI	Y	Lokiarchaeota archaeon-6	2053489	Archaea	Asgard group	N
GCA 021498125	NCBI	Y	Thorarchaeota archaeon-8	2053491	Archaea	Asgard group	N
GCA 021513695	NCBI	Y	Heimdallarchaeota archaeon-5	2876573	Archaea	Asgard group	N
GCA 021513715	NCBI	Y	Heimdallarchaeota archaeon-6	2876572	Archaea	Asgard group	N
GCF 008000775	NCBI	Y	Prometheoarchaeum syntrophicum	2594042	Archaea	Asgard group	N
GCA 001552015	NCBI	Y	Nanopusillus acidilobi	1577684	Archaea	DPANN group	N
GCA 001742785	NCBI	Y	Altiarchaeales archaeon	1878999	Archaea	DPANN group	N
GCA 002686855	NCBI	Y	Woesearchaeota archaeon	2026803	Archaea	DPANN group	N
GCA 002687825	NCBI	Y	Nanoarchaeota archaeon	2026764	Archaea	DPANN group	Y
GCA 002688875	NCBI	Y	Pacearchaeota archaeon	2026773	Archaea	DPANN group	N
GCA 002762975	NCBI	Y	Diapherotrites archaeon	1974407	Archaea	DPANN group	N
GCA 002763085	NCBI	Y	Pacearchaeota archaeon	1974441	Archaea	DPANN group	N
GCA 002778455	NCBI	Y	Micrarchaeota archaeon	1974417	Archaea	DPANN group	N
GCA 002790905	NCBI	Y	Pacearchaeota archaeon	1974434	Archaea	DPANN group	Y

Accession	Data source	Available proteome	Species name	NCBI taxid	Supergroup	Subgroup	In time-resolved species tree
GCA 002792115	NCBI	Y	Woesearchaeota archaeon	1974449	Archaea	DPANN group	N
GCA 002841105	NCBI	Y	Altiarchaeales archaeon	2013673	Archaea	DPANN group	N
GCA 002867475	NCBI	Y	Woesearchaeota archaeon	2026803	Archaea	DPANN group	Y
GCA 003660865	NCBI	Y	Nanohaloarchaeota archaeon	2099673	Archaea	DPANN group	N
GCA 003660905	NCBI	Y	Nanohaloarchaeota archaeon	2099673	Archaea	DPANN group	Y
GCA 009617975	NCBI	Y	Nanohalobium constans	2565781	Archaea	DPANN group	Y
GCA 013426015	NCBI	Y	Fermentimicrarchaeum limneticum	2795018	Archaea	DPANN group	Y
GCA 016187335	NCBI	Y	Aenigmarchaeota archaeon	2093792	Archaea	DPANN group	N
GCA 016188965	NCBI	Y	Micrarchaeota archaeon	2250274	Archaea	DPANN group	Y
GCA 016205145	NCBI	Y	Diapherotrites archaeon	2026736	Archaea	DPANN group	N
GCA 016432325	NCBI	Y	Aenigmarchaeota archaeon	2093792	Archaea	DPANN group	N
GCA 000007185	NCBI	Y	Methanopyrus kandleri	190192	Archaea	Euryarchaeota	Y
GCA 000007305	NCBI	Y	Pyrococcus furiosus	2261	Archaea	Euryarchaeota	Y
GCA 000007345	NCBI	Y	Methanosarcina acetivorans	2214	Archaea	Euryarchaeota	Y
GCA 000008665	NCBI	Y	Archaeoglobus fulgidus	2234	Archaea	Euryarchaeota	Y
GCA 000025505	NCBI	Y	Ferroglobus placidus	589924	Archaea	Euryarchaeota	Y
GCA 000151105	NCBI	N	Thermococcus barophilus	55802	Archaea	Euryarchaeota	Y
GCA 000172995	NCBI	N	Halogeometricum borinquense	60847	Archaea	Euryarchaeota	Y
GCA 000187225	NCBI	N	Haladaptatus paucihalophilus	367189	Archaea	Euryarchaeota	Y
GCA 000328665	NCBI	Y	Methanomethylovorans hollandica	867904	Archaea	Euryarchaeota	Y
GCA 000402775	NCBI	Y	Euryarchaeota archaeon	913322	Archaea	Euryarchaeota	N
GCA 000725425	NCBI	Y	Palaeococcus pacificus	1343739	Archaea	Euryarchaeota	Y
GCA 000789255	NCBI	Y	Geoglobus acetivorans	565033	Archaea	Euryarchaeota	Y
GCA 000970205	NCBI	Y	Methanosarcina mazei	2209	Archaea	Euryarchaeota	Y
GCA 001515185	NCBI	Y	Hadesarchaea archaeon	1775754	Archaea	Euryarchaeota	N
GCA 001563875	NCBI	N	Nanohaloarchaea archaeon	1729453	Archaea	Euryarchaeota	N
GCA 001595815	NCBI	Y	Theionarchaea archaeon	1803814	Archaea	Euryarchaeota	Y
GCA 001761425	NCBI	Y	Nanohaloarchaea archaeon	1737403	Archaea	Euryarchaeota	Y
GCA 001914405	NCBI	Y	Methanohalarchaeum thermophilum	1903181	Archaea	Euryarchaeota	Y
GCA 002813695	NCBI	Y	Methanobacterium subterraneum	59277	Archaea	Euryarchaeota	Y
GCA 002841675	NCBI	Y	Euryarchaeota archaeon	2013767	Archaea	Euryarchaeota	N
GCA 002906575	NCBI	Y	Salinigranum rubrum	755307	Archaea	Euryarchaeota	Y
GCA 003141075	NCBI	N	Euryarchaeota archaeon	2026739	Archaea	Euryarchaeota	N
GCA 003170935	NCBI	N	Euryarchaeota archaeon	2026739	Archaea	Euryarchaeota	Y
GCA 004212085	NCBI	Y	Methanolliviera hydrocarbonicum	2491085	Archaea	Euryarchaeota	Y
GCA 004347835	NCBI	Y	Hadesarchaea archaeon	2250276	Archaea	Euryarchaeota	N
GCA 018141185	NCBI	Y	Methanobacterium alkalithermotolerans	2731220	Archaea	Euryarchaeota	Y
GCA 000012285	NCBI	Y	Sulfolobus acidocaldarius	2285	Archaea	TACK group	Y
GCA 000019605	NCBI	Y	Korarchaeum cryptofilum	498846	Archaea	TACK group	N
GCA 000402095	NCBI	N	Crenarchaeota archaeon	1052804	Archaea	TACK group	Y
GCA 000802205	NCBI	N	Nitrosocosmicus oleophilus	1353260	Archaea	TACK group	Y
GCA 001870125	NCBI	Y	Nitrosocosmicus hydrocola	1826872	Archaea	TACK group	N
GCA 002011035	NCBI	N	Bathyarchaeota archaeon	1935119	Archaea	TACK group	Y

Accession	Data source	Available proteome	Species name	NCBI taxid	Supergroup	Subgroup	In time-resolved species tree
GCA 002011075	NCBI	N	Geothermarchaeota archaeon	1935120	Archaea	TACK group	N
GCA 003019535	NCBI	Y	Marsarchaeota G2	1978159	Archaea	TACK group	Y
GCA 003201765	NCBI	Y	Acidianus sulfidivorans	312539	Archaea	TACK group	Y
GCA 003344655	NCBI	Y	Korarchaeota archaeon	1868214	Archaea	TACK group	N
GCA 004028775	NCBI	Y	Methanosuratincola subterraneus	2593994	Archaea	TACK group	Y
GCA 005888735	NCBI	Y	Bathyarchaeota archaeon	2026714	Archaea	TACK group	N
GCA 011051215	NCBI	N	Bathyarchaeota archaeon	2026714	Archaea	TACK group	N
GCA 015523545	NCBI	N	Geothermarchaeota archaeon	2250273	Archaea	TACK group	Y
GCA 015660995	NCBI	N	Methanomethyliaceae archaeon	2282146	Archaea	TACK group	Y
GCA 900079115	NCBI	Y	Saccharolobus solfataricus	2287	Archaea	TACK group	Y
GCA 000008265	NCBI	Y	Picrophilus torridus	82076	Archaea	Thermoplasmatota	Y
GCA 000025665	NCBI	Y	Aciduliprofundum boonei	379547	Archaea	Thermoplasmatota	Y
GCA 000246735	NCBI	Y	Poseidoniales archaeon	274854	Archaea	Thermoplasmatota	N
GCA 002078355	NCBI	Y	Ferroplasma acidiphilum	74969	Archaea	Thermoplasmatota	Y
GCA 002496345	NCBI	N	Methanomassiliicoccus sp.	1915537	Archaea	Thermoplasmatota	N
GCA 002497405	NCBI	N	Euryarchaeota archaeon	1915970	Archaea	Thermoplasmatota	N
GCA 002502415	NCBI	N	Euryarchaeota archaeon	1915807	Archaea	Thermoplasmatota	N
GCA 002506485	NCBI	N	Euryarchaeota archaeon	1915770	Archaea	Thermoplasmatota	N
GCA 002509165	NCBI	N	Euryarchaeota archaeon	1915775	Archaea	Thermoplasmatota	N
GCA 002878135	NCBI	Y	Aciduliprofundum sp.	2060325	Archaea	Thermoplasmatota	N
GCA 004525545	NCBI	Y	Methanomassiliicoccus sp.	2528041	Archaea	Thermoplasmatota	Y
GCA 013415865	NCBI	Y	Methanomassiliicoccales archaeon	1906667	Archaea	Thermoplasmatota	Y
GCA 900090055	NCBI	Y	Cuniculiplasma divulgatum	1673428	Archaea	Thermoplasmatota	Y
GCA 000428885	NCBI	Y	Geothrix fermentans	44676	Bacteria	Acidobacteria	Y
GCA 001618865	NCBI	Y	Luteitalea pratensis	1855912	Bacteria	Acidobacteria	Y
GCA 001664505	NCBI	Y	Acidobacteria bacterium	1660251	Bacteria	Acidobacteria	N
GCA 000010785	NCBI	Y	Hydrogenobacter thermophilus	940	Bacteria	Aquificae	N
GCA 000021545	NCBI	Y	Sulfurihydrogenibium azorense	204536	Bacteria	Aquificae	N
GCA 015775515	NCBI	Y	Atribacter laminatus	2847778	Bacteria	Atribacterota	Y
GCA 000284335	NCBI	Y	Caldisericum exile	511051	Bacteria	Caldiserica-Cryosericota group	Y
GCA 001886815	NCBI	Y	Caldithrix abyssi	880073	Bacteria	Calditrichaeota	Y
GCA 000177635	NCBI	N	Desulfurispirillum indicum	653733	Bacteria	Chrysiogenetes	N
GCA 000020945	NCBI	Y	Coprothermobacter proteolyticus	309798	Bacteria	Coprothermobacter ota	Y
GCA 013178255	NCBI	Y	Coprothermobacteraceae bacterium	2736298	Bacteria	Coprothermobacter ota	N
GCA 000010985	NCBI	Y	Deferribacter desulfuricans	639282	Bacteria	Deferribacteres	Y
GCA 000025725	NCBI	Y	Denitrovibrio acetiphilus	522772	Bacteria	Deferribacteres	Y
GCA 000020965	NCBI	Y	Dictyoglomus thermophilum	309799	Bacteria	Dictyoglomi	Y
GCA 000021645	NCBI	Y	Dictyoglomus turgidum	515635	Bacteria	Dictyoglomi	N
GCA 000020145	NCBI	Y	Elusimicrobium minutum	445932	Bacteria	Elusimicrobia	Y
GCA 001027545	NCBI	Y	Endomicrobium proavitum	1408281	Bacteria	Elusimicrobia	Y
GCA 002355835	NCBI	Y	Candidatus Endomicrobium	1408204	Bacteria	Elusimicrobia	N
GCA 000020625	NCBI	Y	Prosthecochloris aestuarii	1102	Bacteria	FCB group	Y
GCA 000020645	NCBI	Y	Pelodictyon phaeoclathratiforme	34090	Bacteria	FCB group	Y
GCA 000246855	NCBI	Y	Niastella koreensis	700598	Bacteria	FCB group	Y
GCA 001536145	NCBI	Y	Candidatus Kryptonium	1633631	Bacteria	FCB group	N

Accession	Data source	Available proteome	Species name	NCBI taxid	Supergroup	Subgroup	In time-resolved species tree
GCA 001715195	NCBI	Y	Rhodohalobacter halophilus	1812810	Bacteria	FCB group	Y
GCA 002257665	NCBI	Y	Rubricoccus marinus	716817	Bacteria	FCB group	Y
GCA 900176595	NCBI	Y	Aquiflexum balticum	758820	Bacteria	FCB group	Y
GCA 000023905	NCBI	Y	Leptotrichia buccalis	523794	Bacteria	Fusobacteria	Y
GCA 003019675	NCBI	Y	Fusobacterium ulcerans	861	Bacteria	Fusobacteria	Y
GCA 000341545	NCBI	Y	Nitrospina gracilis	1266370	Bacteria	Nitrospinae-Tectomicrobia group	Y
GCA 000020985	NCBI	Y	Thermodesulfovibrio yellowstonii	289376	Bacteria	Nitrospirae	Y
GCA 000284315	NCBI	Y	Leptospirillum ferrooxidans	180	Bacteria	Nitrospirae	Y
GCA 001273775	NCBI	Y	Nitrospira moscoviensis	42253	Bacteria	Nitrospirae	Y
GCA 000012685	NCBI	Y	Myxococcus xanthus	34	Bacteria	Proteobacteria	Y
GCA 000014865	NCBI	Y	Magnetococcus marinus	156889	Bacteria	Proteobacteria	Y
GCA 000165485	NCBI	Y	Stigmatella aurantiaca	41	Bacteria	Proteobacteria	Y
GCA 000196175	NCBI	Y	Bdellovibrio bacteriovorus	959	Bacteria	Proteobacteria	Y
GCA 000236665	NCBI	Y	Azospira oryzae	146939	Bacteria	Proteobacteria	Y
GCA 000242915	NCBI	N	Sulfurimonas gotlandica	929558	Bacteria	Proteobacteria	Y
GCA 000419525	NCBI	Y	Leucothrix mucor	998674	Bacteria	Proteobacteria	Y
GCA 000429065	NCBI	Y	Silanimonas lenta	265429	Bacteria	Proteobacteria	N
GCA 000430805	NCBI	Y	Chitinilyticum aquatile	362520	Bacteria	Proteobacteria	Y
GCA 000515255	NCBI	Y	Rhodovibrio salinarum	1089552	Bacteria	Proteobacteria	N
GCA 000987835	NCBI	Y	Sulfurovum lithotrophicum	206403	Bacteria	Proteobacteria	Y
GCA 001748245	NCBI	Y	Oligoflexus tunisiensis	708132	Bacteria	Proteobacteria	Y
GCA 001783715	NCBI	Y	Lambdaproteobacteria bacterium	1817773	Bacteria	Proteobacteria	Y
GCA 001931535	NCBI	Y	Minicystis rosea	888845	Bacteria	Proteobacteria	Y
GCA 002007485	NCBI	Y	Bartonella apihabitans	2750929	Bacteria	Proteobacteria	Y
GCA 002043005	NCBI	Y	Martelella mediterranea	293089	Bacteria	Proteobacteria	Y
GCA 002117145	NCBI	Y	Nucleicultrix amoebiphila	1414854	Bacteria	Proteobacteria	N
GCA 002117405	NCBI	Y	Methylocystis bryophila	655015	Bacteria	Proteobacteria	Y
GCA 002119765	NCBI	Y	Pseudorhodoplanes sinuspersici	1235591	Bacteria	Proteobacteria	Y
GCA 002215215	NCBI	Y	Granulosicoccus antarcticus	1192854	Bacteria	Proteobacteria	N
GCA 002795805	NCBI	Y	Mariprofundus aestuarium	1921086	Bacteria	Proteobacteria	Y
GCA 003711085	NCBI	Y	Arcobacter peruensis	2320140	Bacteria	Proteobacteria	Y
GCA 003722335	NCBI	Y	Caulobacter flavus	1679497	Bacteria	Proteobacteria	Y
GCA 003966715	NCBI	Y	Blastochloris tepida	2233851	Bacteria	Proteobacteria	N
GCA 003970735	NCBI	Y	Silicimonas algicola	1826607	Bacteria	Proteobacteria	Y
GCA 004209755	NCBI	Y	Pseudoduganella lutea	321985	Bacteria	Proteobacteria	Y
GCA 005144885	NCBI	Y	Phreatobacter stygius	1940610	Bacteria	Proteobacteria	Y
GCA 005862305	NCBI	Y	Rhizobium indicum	2583231	Bacteria	Proteobacteria	Y
GCA 006385135	NCBI	Y	Paremcibacter congregatus	2043170	Bacteria	Proteobacteria	Y
GCA 009789575	NCBI	Y	Stappia indica	538381	Bacteria	Proteobacteria	Y
GCA 012276695	NCBI	Y	Pelagibacter giovannonii	2563896	Bacteria	Proteobacteria	N
GCA 014189455	NCBI	Y	Mesorhizobium huakuii	28104	Bacteria	Proteobacteria	Y
GCA 015240255	NCBI	Y	Kordiimonas pumila	2161677	Bacteria	Proteobacteria	Y
GCA 015482775	NCBI	Y	Kaustia mangrovi	2593653	Bacteria	Proteobacteria	Y
GCA 016807805	NCBI	Y	Xanthobacter dioxanivorans	2528964	Bacteria	Proteobacteria	Y
GCA 017161425	NCBI	Y	Marinicauda algicola	2029849	Bacteria	Proteobacteria	Y
GCA 017347565	NCBI	Y	Methylobacterium indicum	1775910	Bacteria	Proteobacteria	Y
GCA 017493175	NCBI	Y	Bradyrhizobium quebecense	2748629	Bacteria	Proteobacteria	Y
GCA 018417475	NCBI	Y	Novosphingobium decolorationis	2698673	Bacteria	Proteobacteria	Y

Accession	Data source	Available proteome	Species name	NCBI taxid	Supergroup	Subgroup	In time-resolved species tree
GCA 018436245	NCBI	Y	Pseudochrobactrum algeriensis	2834768	Bacteria	Proteobacteria	Y
GCA 024662015	NCBI	Y	Nisaea acidiphila	1862145	Bacteria	Proteobacteria	N
GCA 025136295	NCBI	Y	Aestuariispira ectoiniformans	2775080	Bacteria	Proteobacteria	N
GCA 026409565	NCBI	Y	Sneathiella aquimaris	2599305	Bacteria	Proteobacteria	Y
GCA 947250595	NCBI	Y	Wolbachia endosymbiont	2953990	Bacteria	Proteobacteria	Y
GCF 000024765	NCBI	Y	Halothiobacillus neapolitanus	927	Bacteria	Proteobacteria	Y
GCF 000378965	NCBI	Y	Thioalkalivibrio thiocyanodenitrificans	243063	Bacteria	Proteobacteria	Y
GCF 000633935	NCBI	Y	Ectothiorhodospira haloalkaliphila	421628	Bacteria	Proteobacteria	Y
GCF 001677435	NCBI	Y	Woeseia oceani	1548547	Bacteria	Proteobacteria	N
GCF 002078315	NCBI	Y	Rickettsia bellii	33990	Bacteria	Proteobacteria	Y
GCF 003403095	NCBI	Y	Thalassospira indica	1891279	Bacteria	Proteobacteria	Y
GCF 004135935	NCBI	Y	Methylovirgula ligni	569860	Bacteria	Proteobacteria	Y
GCF 004421105	NCBI	Y	Nitrosococcus wardiae	1814290	Bacteria	Proteobacteria	N
GCF 008728195	NCBI	Y	Roseovarius indicus	540747	Bacteria	Proteobacteria	Y
GCF 008728855	NCBI	Y	Hypericibacter terrae	2602015	Bacteria	Proteobacteria	Y
GCF 013201075	NCBI	Y	Lichenicola cladoniae	1484109	Bacteria	Proteobacteria	Y
GCF 016765655	NCBI	Y	Skermanella mucosa	1789672	Bacteria	Proteobacteria	Y
GCF 019203965	NCBI	Y	Elioraea tepida	2843330	Bacteria	Proteobacteria	Y
GCF 019504385	NCBI	Y	Devosia salina	2860336	Bacteria	Proteobacteria	Y
GCF 900231165	NCBI	Y	Hartmannibacter diazotrophicus	1482074	Bacteria	Proteobacteria	Y
GCF 002109495	NCBI	Y	Magnetofaba australis	1472297	Bacteria	Proteobacteria	Y
GCF 004217665	NCBI	Y	Magnetaquicoccus inordinatus	2496818	Bacteria	Proteobacteria	Y
GCF 012790675	NCBI	Y	Anaplasma platys	949	Bacteria	Proteobacteria	Y
GCA 000019965	NCBI	Y	Opitutus terrae	107709	Bacteria	PVC group	Y
GCA 000025905	NCBI	Y	Coraliomargarita akajimensis	583355	Bacteria	PVC group	Y
GCA 000092785	NCBI	Y	Waddlia chondrophila	716544	Bacteria	PVC group	Y
GCA 000172155	NCBI	Y	Verrucomicrobium spinosum	240016	Bacteria	PVC group	Y
GCA 000196115	NCBI	Y	Rhodopirellula baltica	243090	Bacteria	PVC group	N
GCA 000237205	NCBI	Y	Simkania negevensis	331113	Bacteria	PVC group	Y
GCA 000378105	NCBI	Y	Rubritalea marina	361055	Bacteria	PVC group	Y
GCA 001017655	NCBI	Y	Kiritimatiella glycovorans	1307763	Bacteria	PVC group	Y
GCA 001642915	NCBI	Y	Roseimaritima ulvae	980254	Bacteria	PVC group	N
GCA 001805325	NCBI	Y	Omnitrophica bacterium	1801819	Bacteria	PVC group	N
GCA 002792715	NCBI	Y	Candidatus Omnitrophica	1974733	Bacteria	PVC group	N
GCA 002998355	NCBI	Y	Victivallales bacterium	2094242	Bacteria	PVC group	Y
GCA 009002475	NCBI	Y	Uabimicrobium amorphum	2596890	Bacteria	PVC group	N
GCA 012103575	NCBI	Y	Kiritimatiellae bacterium	2212473	Bacteria	PVC group	N
GCA 000017605	NCBI	Y	Leptospira biflexa	172	Bacteria	Spirochaetes	Y
GCA 000208385	NCBI	Y	Sphaerochaeta coccoides	273376	Bacteria	Spirochaetes	Y
GCA 000266885	NCBI	Y	Turneriella parva	869212	Bacteria	Spirochaetes	Y
GCA 000507245	NCBI	Y	Salinispira pacifica	1307761	Bacteria	Spirochaetes	Y
GCA 000165795	NCBI	Y	Aminomonas paucivorans	584708	Bacteria	Synergistetes	Y
GCA 900177735	NCBI	Y	Dethiosulfovibrio salsuginis	561720	Bacteria	Synergistetes	Y
GCA 000011245	NCBI	Y	Oceanobacillus iheyensis	182710	Bacteria	Terrabacteria group	Y
GCA 000011905	NCBI	Y	Dehalococcoides mccartyi	61435	Bacteria	Terrabacteria group	Y
GCA 000017805	NCBI	Y	Roseiflexus castenholzii	383372	Bacteria	Terrabacteria group	Y
GCA 000018105	NCBI	Y	Acaryochloris marina	329726	Bacteria	Terrabacteria group	Y
GCA 000018865	NCBI	Y	Chloroflexus aurantiacus	1108	Bacteria	Terrabacteria group	Y
GCA 000021685	NCBI	Y	Thermomicrobium roseum	500	Bacteria	Terrabacteria group	Y
GCA 000089865	NCBI	Y	Mycoplasmopsis agalactiae	2110	Bacteria	Terrabacteria group	Y

Accession	Data source	Available proteome	Species name	NCBI taxid	Supergroup	Subgroup	In time-resolved species tree
GCA 000092125	NCBI	Y	Meiothermus silvanus	52022	Bacteria	Terrabacteria group	Y
GCA 000092425	NCBI	Y	Truepera radiovictrix	649638	Bacteria	Terrabacteria group	Y
GCA 000143165	NCBI	Y	Dehalogenimonas lykanthroporepellens	552810	Bacteria	Terrabacteria group	Y
GCA 000154485	NCBI	Y	Erysipelatoclostridium ramosum	1547	Bacteria	Terrabacteria group	Y
GCA 000204075	NCBI	Y	Trichormus variabilis	264691	Bacteria	Terrabacteria group	Y
GCA 000237085	NCBI	Y	Acetivibrio clariflavus	720554	Bacteria	Terrabacteria group	Y
GCA 000270285	NCBI	Y	Eggerthella sp.	502558	Bacteria	Terrabacteria group	Y
GCA 000317285	NCBI	Y	Chlorogloeopsis fritschii	1124	Bacteria	Terrabacteria group	Y
GCA 000427095	NCBI	Y	Chthonomonas calidirosea	1303518	Bacteria	Terrabacteria group	Y
GCA 000430605	NCBI	Y	Anaeroarcus burkinensis	82376	Bacteria	Terrabacteria group	Y
GCA 000702505	NCBI	Y	Thermogemmatispora carboxidivorans	1382306	Bacteria	Terrabacteria group	Y
GCA 000724625	NCBI	Y	Fimbriimonas ginsengisoli	661478	Bacteria	Terrabacteria group	Y
GCA 000949295	NCBI	Y	Acidithrix ferrooxidans	1280514	Bacteria	Terrabacteria group	Y
GCA 000967915	NCBI	Y	Paracholeplasma brassicae	61635	Bacteria	Terrabacteria group	Y
GCA 001007995	NCBI	Y	Deinococcus soli	1309411	Bacteria	Terrabacteria group	Y
GCA 001278705	NCBI	Y	Bacillus gobiensis	1441095	Bacteria	Terrabacteria group	Y
GCA 001306115	NCBI	Y	Ornatilinea apprima	1134406	Bacteria	Terrabacteria group	Y
GCA 001653075	NCBI	Y	Planomonospora sphaerica	161355	Bacteria	Terrabacteria group	Y
GCA 001945525	NCBI	Y	Ileibacterium valens	1862668	Bacteria	Terrabacteria group	Y
GCA 002214185	NCBI	Y	Streptomyces capitiformicae	2014920	Bacteria	Terrabacteria group	Y
GCA 003013295	NCBI	Y	Williamsoniiplasma luminosum	214888	Bacteria	Terrabacteria group	N
GCA 004208415	NCBI	Y	Ktedonosporobacter rubrisoli	2509675	Bacteria	Terrabacteria group	Y
GCA 007066085	NCBI	Y	Paenibacillus thiaminolyticus	49283	Bacteria	Terrabacteria group	Y
GCA 008705175	NCBI	Y	Clostridium diolis	223919	Bacteria	Terrabacteria group	Y
GCA 900104725	NCBI	Y	Actinoplanes derwentensis	113562	Bacteria	Terrabacteria group	Y
GCA 900106765	NCBI	Y	Tepidimicrobium xylanilyticum	1123352	Bacteria	Terrabacteria group	Y
GCA 900110485	NCBI	Y	Propionispora vibrioides	112903	Bacteria	Terrabacteria group	Y
GCA 900119385	NCBI	Y	Thermophilibacter mediterraneus	1871031	Bacteria	Terrabacteria group	Y
GCA 900128965	NCBI	Y	Ferrithrix thermotolerans	209649	Bacteria	Terrabacteria group	Y
GCA 900142005	NCBI	Y	Dethiosulfatibacter aminovorans	332095	Bacteria	Terrabacteria group	Y
GCA 900184705	NCBI	Y	Brevefilum fermentans	1986204	Bacteria	Terrabacteria group	Y
GCA 900187885	NCBI	Y	Thermoflexus hugenholtzii	1495650	Bacteria	Terrabacteria group	Y
GCA 900188145	NCBI	Y	Anaerovirgula multivorans	312168	Bacteria	Terrabacteria group	Y
GCF 000012525	NCBI	Y	Synechococcus elongatus	32046	Bacteria	Terrabacteria group	Y
GCF 000015665	NCBI	Y	Prochlorococcus marinus	1219	Bacteria	Terrabacteria group	Y
GCF 000484535	NCBI	Y	Gloeobacter kilaueensis	1416614	Bacteria	Terrabacteria group	Y
GCA 000421585	NCBI	Y	Thermodesulfatator atlanticus	501497	Bacteria	Thermodesulfobacteria	Y
GCA 000746255	NCBI	Y	Thermodesulfobacterium hydrogeniphilum	161156	Bacteria	Thermodesulfobacteria	Y
GCA 000023325	NCBI	Y	Kosmotoga olearia	521045	Bacteria	Thermotogae	Y
GCA 000767275	NCBI	N	Fervidobacterium islandicum	2423	Bacteria	Thermotogae	Y
GCA 002895525	NCBI	Y	Petrotoga olearia	156203	Bacteria	Thermotogae	Y
GCA 004117075	NCBI	Y	Fervidobacterium changbaicum	310769	Bacteria	Thermotogae	N
EP00006	EukProt	Y	Acanthamoeba castellanii	5755	Eukaryota	Amoebozoa	Y
EP00013	EukProt	Y	Stygamoeba regulata	1042828	Eukaryota	Amoebozoa	N

Accession	Data source	Available proteome	Species name	NCBI taxid	Supergroup	Subgroup	In time-resolved species tree
EP00019	EukProt	Y	Armaparvus languidus	1280930	Eukaryota	Amoebozoa	N
EP00020	EukProt	Y	Sapocribrum chincoteaguense	2605440	Eukaryota	Amoebozoa	N
EP00023	EukProt	Y	Dictyostelium discoideum	44689	Eukaryota	Amoebozoa	Y
EP00026	EukProt	Y	Physarum polycephalum	5791	Eukaryota	Amoebozoa	N
EP00029	EukProt	Y	Vermamoeba vermiformis	5778	Eukaryota	Amoebozoa	N
EP01087	EukProt	Y	Luapelamoeba hula	555407	Eukaryota	Amoebozoa	N
EP01089	EukProt	Y	Gocevia fonbrunei	1078849	Eukaryota	Amoebozoa	N
EP01090	EukProt	Y	Pellita catalonica	1485083	Eukaryota	Amoebozoa	N
EP01091	EukProt	Y	Cochliopodium minus	313997	Eukaryota	Amoebozoa	N
EP01094	EukProt	Y	Clydonella sp	218657	Eukaryota	Amoebozoa	N
EP01103	EukProt	Y	Thecamoeba quadrilineata	343530	Eukaryota	Amoebozoa	N
EP01104	EukProt	Y	Vermistella antarctica	354202	Eukaryota	Amoebozoa	N
EP01105	EukProt	Y	Mastigella eilhardi	1580584	Eukaryota	Amoebozoa	N
EP01107	EukProt	Y	Rhizomastix libera	1211407	Eukaryota	Amoebozoa	N
EP01109	EukProt	Y	Acytostelium subglobosum	361139	Eukaryota	Amoebozoa	N
EP01113	EukProt	Y	Clastostelium recurvatum	668366	Eukaryota	Amoebozoa	N
EP01117	EukProt	Y	Protostelium nocturnum	668368	Eukaryota	Amoebozoa	N
EP01119	EukProt	Y	Soliformovum irregulare	435424	Eukaryota	Amoebozoa	N
EP01122	EukProt	Y	Echinamoeba exundans	136450	Eukaryota	Amoebozoa	N
EP01125	EukProt	Y	Pyxidicula operculata	1078852	Eukaryota	Amoebozoa	N
EP01127	EukProt	Y	Copromyxa protea	931554	Eukaryota	Amoebozoa	N
EP01130	EukProt	Y	Rhizamoeba saxonica	200886	Eukaryota	Amoebozoa	N
GCA 000277445	NCBI	Y	Polysphondylium violaceum	133409	Eukaryota	Amoebozoa	Y
EP00031	EukProt	Y	Thecamonas trahens	529818	Eukaryota	Apusozoa	N
EP01139	EukProt	Y	Manchomonas bermudensis	172822	Eukaryota	Apusozoa	N
EP00033	EukProt	Y	Pygsuia biforma	1277256	Eukaryota	Breviatea	N
EP00002	EukProt	Y	Diphylleia rotans	190327	Eukaryota	CRuMs	N
EP00277	EukProt	Y	Geminigera cryophila	46947	Eukaryota	Cryptophyceae	N
EP00279	EukProt	Y	Guillardia theta	55529	Eukaryota	Cryptophyceae	N
EP00282	EukProt	Y	Hemiselmis andersenii	464988	Eukaryota	Cryptophyceae	N
EP00290	EukProt	Y	Baffinella sp	2571260	Eukaryota	Cryptophyceae	N
EP00295	EukProt	Y	Goniomonas pacifica	195067	Eukaryota	Cryptophyceae	N
EP00802	EukProt	Y	Teleaulax amphioxeia	77931	Eukaryota	Cryptophyceae	N
EP00671	EukProt	Y	Bodo saltans	75058	Eukaryota	Discoba	N
EP00674	EukProt	Y	Leishmania major	5664	Eukaryota	Discoba	N
EP00681	EukProt	Y	Trypanosoma brucei	5691	Eukaryota	Discoba	N
EP00686	EukProt	Y	Naegleria gruberi	5762	Eukaryota	Discoba	N
EP00761	EukProt	Y	Pharyngomonas kirbyi	63601	Eukaryota	Discoba	N
EP00762	EukProt	Y	Andalucia godoyi	505711	Eukaryota	Discoba	N
EP01006	EukProt	Y	Ploeotia vitrea	2493237	Eukaryota	Discoba	N
EP01027	EukProt	Y	Heterolobosea sp	5752	Eukaryota	Discoba	N
EP01062	EukProt	Y	Namystynia karyoxenos	2508218	Eukaryota	Discoba	N
EP01065	EukProt	Y	Artemidia motanka	2508217	Eukaryota	Discoba	N
EP01080	EukProt	Y	Neovahlkampfia damariscottae	166956	Eukaryota	Discoba	N
EP00276	EukProt	Y	Gloeochaete wittrockiana	38269	Eukaryota	Glaucocystophyceae	N
EP00741	EukProt	Y	Cyanophora paradoxa	2762	Eukaryota	Glaucocystophyceae	Y
EP00298	EukProt	Y	Acanthocystis sp	299834	Eukaryota	Haptista	N
EP00301	EukProt	Y	Raphidiophrys heterophryoidea	1703429	Eukaryota	Haptista	N
EP00302	EukProt	Y	Diacronema sp	70451	Eukaryota	Haptista	N
EP00306	EukProt	Y	Pavlova sp	2831	Eukaryota	Haptista	N
EP00314	EukProt	Y	Emiliania huxleyi	2903	Eukaryota	Haptista	N

Accession	Data source	Available proteome	Species name	NCBI taxid	Supergroup	Subgroup	In time-resolved species tree
EP00327	EukProt	Y	Prymnesium parvum	97485	Eukaryota	Haptista	N
EP00900	EukProt	Y	Chrysochromulina parva	127563	Eukaryota	Haptista	N
EP00696	EukProt	Y	Hemimastix kukwesjijk	2027451	Eukaryota	Hemimastigophora	N
EP00697	EukProt	Y	Spironema sp	2027454	Eukaryota	Hemimastigophora	N
EP00698	EukProt	Y	Gefionella okellyi	2853422	Eukaryota	Malawimonadida	N
EP00701	EukProt	Y	Giardia lamblia	5741	Eukaryota	Metamonada	N
EP00702	EukProt	Y	Spironucleus salmonicida	348837	Eukaryota	Metamonada	N
EP00703	EukProt	Y	Trepomonas sp	44997	Eukaryota	Metamonada	N
EP00705	EukProt	Y	Trichomonas vaginalis	5722	Eukaryota	Metamonada	N
EP00706	EukProt	Y	Tritrichomonas foetus	1144522	Eukaryota	Metamonada	N
EP00708	EukProt	Y	Paratrimastix pyriformis	342808	Eukaryota	Metamonada	N
EP00767	EukProt	Y	Chilomastix cuspidata	1476591	Eukaryota	Metamonada	N
EP00770	EukProt	Y	Monocercomonoides exilis	2049356	Eukaryota	Metamonada	N
EP00039	EukProt	Y	Didymoeca costata	459534	Eukaryota	Opisthokonta	Y
EP00040	EukProt	Y	Diaphanoeca grandis	28014	Eukaryota	Opisthokonta	N
EP00046	EukProt	Y	Monosiga brevicollis	81824	Eukaryota	Opisthokonta	N
EP00051	EukProt	Y	Salpingoeca urceolata	473813	Eukaryota	Opisthokonta	Y
EP00052	EukProt	Y	Salpingoeca macrocollata	1924736	Eukaryota	Opisthokonta	N
EP00057	EukProt	Y	Strongylocentrotus purpuratus	7668	Eukaryota	Opisthokonta	Y
EP00058	EukProt	Y	Branchiostoma floridae	7739	Eukaryota	Opisthokonta	N
EP00059	EukProt	Y	Ciona intestinalis	7719	Eukaryota	Opisthokonta	N
EP00060	EukProt	Y	Petromyzon marinus	7757	Eukaryota	Opisthokonta	N
EP00062	EukProt	Y	Callorhinchus milii	7868	Eukaryota	Opisthokonta	N
EP00063	EukProt	Y	Salmo salar	8030	Eukaryota	Opisthokonta	Y
EP00064	EukProt	Y	Thunnus orientalis	8238	Eukaryota	Opisthokonta	Y
EP00067	EukProt	Y	Danio rerio	7955	Eukaryota	Opisthokonta	Y
EP00068	EukProt	Y	Latimeria chalumnae	7897	Eukaryota	Opisthokonta	N
EP00072	EukProt	Y	Mus musculus	10090	Eukaryota	Opisthokonta	Y
EP00075	EukProt	Y	Anas platyrhynchos	8839	Eukaryota	Opisthokonta	Y
EP00076	EukProt	Y	Gallus gallus	9031	Eukaryota	Opisthokonta	Y
EP00077	EukProt	Y	Pelodiscus sinensis	13735	Eukaryota	Opisthokonta	Y
EP00078	EukProt	Y	Anolis carolinensis	28377	Eukaryota	Opisthokonta	N
EP00079	EukProt	Y	Xenopus tropicalis	8364	Eukaryota	Opisthokonta	Y
EP00083	EukProt	Y	Stegodyphus mimosarum	407821	Eukaryota	Opisthokonta	Y
EP00090	EukProt	Y	Calanus glacialis	113644	Eukaryota	Opisthokonta	Y
EP00092	EukProt	Y	Neocalanus flemingeri	119369	Eukaryota	Opisthokonta	Y
EP00098	EukProt	Y	Tribolium castaneum	7070	Eukaryota	Opisthokonta	Y
EP00099	EukProt	Y	Drosophila melanogaster	7227	Eukaryota	Opisthokonta	Y
EP00103	EukProt	Y	Capitella teleta	283909	Eukaryota	Opisthokonta	Y
EP00104	EukProt	Y	Lingula anatina	7574	Eukaryota	Opisthokonta	N
EP00105	EukProt	Y	Crassostrea gigas	29159	Eukaryota	Opisthokonta	Y
EP00106	EukProt	Y	Octopus bimaculoides	37653	Eukaryota	Opisthokonta	Y
EP00107	EukProt	Y	Lottia gigantea	225164	Eukaryota	Opisthokonta	Y
EP00108	EukProt	Y	Taenia solium	6204	Eukaryota	Opisthokonta	N
EP00109	EukProt	Y	Schistosoma mansoni	6183	Eukaryota	Opisthokonta	N
EP00110	EukProt	Y	Nematostella vectensis	45351	Eukaryota	Opisthokonta	N
EP00111	EukProt	Y	Clytia hemisphaerica	252671	Eukaryota	Opisthokonta	Y
EP00113	EukProt	Y	Trichoplax adhaerens	10228	Eukaryota	Opisthokonta	Y
EP00114	EukProt	Y	Trichoplax sp	10227	Eukaryota	Opisthokonta	N
EP00115	EukProt	Y	Mnemiopsis leidyi	27923	Eukaryota	Opisthokonta	N
EP00117	EukProt	Y	Sycon ciliatum	27933	Eukaryota	Opisthokonta	N
EP00119	EukProt	Y	Amphimedon queenslandica	400682	Eukaryota	Opisthokonta	Y
EP00120	EukProt	Y	Capsaspora owczarzaki	192875	Eukaryota	Opisthokonta	N
EP00123	EukProt	Y	Amoebidium parasiticum	4881	Eukaryota	Opisthokonta	N
EP00127	EukProt	Y	Corallochytrium limacisporum	98351	Eukaryota	Opisthokonta	N

Accession	Data source	Available proteome	Species name	NCBI taxid	Supergroup	Subgroup	In time-resolved species tree
EP00129	EukProt	Y	Allomyces macrogynus	28583	Eukaryota	Opisthokonta	N
EP00130	EukProt	Y	Batrachochytrium dendrobatidis	109871	Eukaryota	Opisthokonta	Y
EP00131	EukProt	Y	Spizellomyces punctatus	109760	Eukaryota	Opisthokonta	N
EP00134	EukProt	Y	Neurospora crassa	5141	Eukaryota	Opisthokonta	Y
EP00143	EukProt	Y	Candida glabrata	5478	Eukaryota	Opisthokonta	N
EP00144	EukProt	Y	Saccharomyces cerevisiae	4932	Eukaryota	Opisthokonta	Y
EP00145	EukProt	Y	Schizosaccharomyces pombe	4896	Eukaryota	Opisthokonta	N
EP00146	EukProt	Y	Coprinopsis cinerea	5346	Eukaryota	Opisthokonta	N
EP00150	EukProt	Y	Ustilago maydis	5270	Eukaryota	Opisthokonta	Y
EP00158	EukProt	Y	Paraphelidium tribonemae	2548444	Eukaryota	Opisthokonta	N
EP00159	EukProt	Y	Fonticula alba	691883	Eukaryota	Opisthokonta	N
EP00794	EukProt	Y	Sanderia malayensis	128131	Eukaryota	Opisthokonta	Y
EP00795	EukProt	Y	Rhopilema esculentum	499914	Eukaryota	Opisthokonta	Y
EP00857	EukProt	Y	Cystobasidium minutum	29899	Eukaryota	Opisthokonta	Y
EP00865	EukProt	Y	Coemansia reversa	61392	Eukaryota	Opisthokonta	N
EP01134	EukProt	Y	Creolimax fragrantissima	470921	Eukaryota	Opisthokonta	Y
EP01135	EukProt	Y	Chromosphaera perkinsii	127916	Eukaryota	Opisthokonta	Y
EP01147	EukProt	Y	Barroeca monosierra	2830476	Eukaryota	Opisthokonta	N
GCA 022478865	NCBI	Y	Lamellibrachia satsuma	104711	Eukaryota	Opisthokonta	Y
GCF 000300575	NCBI	Y	Agaricus bisporus	5341	Eukaryota	Opisthokonta	N
GCF 000326865	NCBI	Y	Helobdella robusta	6412	Eukaryota	Opisthokonta	Y
GCF 000517525	NCBI	Y	Limulus polyphemus	6850	Eukaryota	Opisthokonta	Y
GCF 018143015	NCBI	Y	Lytechinus variegatus	7654	Eukaryota	Opisthokonta	Y
GCF 022113875	NCBI	Y	Hydra vulgaris	6087	Eukaryota	Opisthokonta	Y
REFERENCE	EukProt	Y	Homo sapiens	9606	Eukaryota	Opisthokonta	Y
EP00735	EukProt	Y	Rhodelphis limneticus	2583502	Eukaryota	Rhodelphea	N
EP00736	EukProt	Y	Rhodelphis marinus	2584957	Eukaryota	Rhodelphea	N
EP00165	EukProt	Y	Cyanidioschyzon merolae	45157	Eukaryota	Rhodophyta	Y
EP00167	EukProt	Y	Galdieria sulphuraria	130081	Eukaryota	Rhodophyta	N
EP00168	EukProt	Y	Porphyra purpurea	2787	Eukaryota	Rhodophyta	N
EP00169	EukProt	Y	Porphyra umbilicalis	2786	Eukaryota	Rhodophyta	N
EP00176	EukProt	Y	Chondrus crispus	2769	Eukaryota	Rhodophyta	Y
EP00179	EukProt	Y	Madagascaria erythrocladioides	753684	Eukaryota	Rhodophyta	N
EP00181	EukProt	Y	Compsopogon caeruleus	31354	Eukaryota	Rhodophyta	N
EP00184	EukProt	Y	Porphyridium aerugineum	2792	Eukaryota	Rhodophyta	N
EP00185	EukProt	Y	Porphyridium purpureum	35688	Eukaryota	Rhodophyta	N
EP00187	EukProt	Y	Rhodella violacea	2801	Eukaryota	Rhodophyta	Y
EP00189	EukProt	Y	Rhodosorus marinus	101924	Eukaryota	Rhodophyta	N
EP00190	EukProt	Y	Stylonematophyceae sp	139979	Eukaryota	Rhodophyta	N
EP00871	EukProt	Y	Galdieria phlegrea	1389228	Eukaryota	Rhodophyta	N
EP00872	EukProt	Y	Gracilariopsis chorda	448386	Eukaryota	Rhodophyta	Y
EP00333	EukProt	Y	Tetrahymena thermophila	5911	Eukaryota	Sar	Y
EP00334	EukProt	Y	Paramecium tetraurelia	5888	Eukaryota	Sar	N
EP00339	EukProt	Y	Tiarina fusus	693140	Eukaryota	Sar	N
EP00348	EukProt	Y	Stylonychia lemnae	5949	Eukaryota	Sar	N
EP00364	EukProt	Y	Plasmodium chabaudi	5825	Eukaryota	Sar	N
EP00370	EukProt	Y	Babesia bigemina	5866	Eukaryota	Sar	N
EP00372	EukProt	Y	Theileria equi	5872	Eukaryota	Sar	Y
EP00377	EukProt	Y	Hammondia hammondi	99158	Eukaryota	Sar	Y
EP00379	EukProt	Y	Toxoplasma gondii	5811	Eukaryota	Sar	N
EP00390	EukProt	Y	Vitrella brassicaformis	1169539	Eukaryota	Sar	N
EP00397	EukProt	Y	Hematodinium sp	135491	Eukaryota	Sar	N
EP00428	EukProt	Y	Durinskia baltica	400754	Eukaryota	Sar	N
EP00454	EukProt	Y	Perkinsus marinus	31276	Eukaryota	Sar	Y
EP00455	EukProt	Y	Lapot gusevi	2072850	Eukaryota	Sar	N

Accession	Data source	Available proteome	Species name	NCBI taxid	Supergroup	Subgroup	In time-resolved species tree
EP00457	EukProt	Y	Paulinella chromatophora	39717	Eukaryota	Sar	N
EP00466	EukProt	Y	Bigelowiella natans	227086	Eukaryota	Sar	N
EP00467	EukProt	Y	Chlorarachnion reptans	29199	Eukaryota	Sar	N
EP00473	EukProt	Y	Plasmodiophora brassicae	37360	Eukaryota	Sar	N
EP00475	EukProt	Y	Leptophrys vorax	1108494	Eukaryota	Sar	N
EP00480	EukProt	Y	Reticulomyxa filosa	46433	Eukaryota	Sar	N
EP00485	EukProt	Y	Elphidium margaritaceum	933848	Eukaryota	Sar	N
EP00512	EukProt	Y	Aurantiochytrium limacinum	87102	Eukaryota	Sar	N
EP00513	EukProt	Y	Hondaea fermentalgiana	2315210	Eukaryota	Sar	N
EP00530	EukProt	Y	Phaeodactylum tricornutum	2850	Eukaryota	Sar	N
EP00609	EukProt	Y	Ectocarpus siliculosus	2880	Eukaryota	Sar	Y
EP00625	EukProt	Y	Aureococcus anophagefferens	44056	Eukaryota	Sar	Y
EP00645	EukProt	Y	Phytophthora infestans	4787	Eukaryota	Sar	Y
EP00654	EukProt	Y	Saprolegnia diclina	112098	Eukaryota	Sar	N
EP00655	EukProt	Y	Saprolegnia parasitica	101203	Eukaryota	Sar	Y
EP00742	EukProt	Y	Colponemidia sp	1492816	Eukaryota	Sar	N
EP00743	EukProt	Y	Colponemidia sp	1492816	Eukaryota	Sar	N
EP00749	EukProt	Y	Cafeteria roenbergensis	2604071	Eukaryota	Sar	N
EP00750	EukProt	Y	Incisomonas marina	1167876	Eukaryota	Sar	Y
EP00797	EukProt	Y	Seminavis robusta	568900	Eukaryota	Sar	N
EP00808	EukProt	Y	Paulinella micropora	1928728	Eukaryota	Sar	N
EP00810	EukProt	Y	Oxytricha trifallax	1172189	Eukaryota	Sar	Y
EP00813	EukProt	Y	Pythium oligandrum	41045	Eukaryota	Sar	Y
EP00820	EukProt	Y	Chromera velia	505693	Eukaryota	Sar	N
EP00822	EukProt	Y	Plasmodium yoelii	5861	Eukaryota	Sar	N
EP00901	EukProt	Y	Cardiosporidium cionae	476202	Eukaryota	Sar	N
EP00914	EukProt	Y	Ancora sagittata	1892433	Eukaryota	Sar	N
EP00921	EukProt	Y	Rhytidocystis pertsovi	2719953	Eukaryota	Sar	N
EP00922	EukProt	Y	Rhytidocystis sp	358187	Eukaryota	Sar	N
EP00924	EukProt	Y	Labyrinthula sp	35133	Eukaryota	Sar	N
EP00927	EukProt	Y	Polykrikos kofoidii	271679	Eukaryota	Sar	N
EP00931	EukProt	Y	Biecheleriopsis adriatica	1407008	Eukaryota	Sar	N
EP01082	EukProt	Y	Thalassiosira pseudonana	35128	Eukaryota	Sar	Y
EP01083	EukProt	Y	Nonionella stella	191124	Eukaryota	Sar	N
EP01084	EukProt	Y	Bolivina argentea	1132577	Eukaryota	Sar	N
EP01138	EukProt	Y	Halocafeteria seosinensis	357350	Eukaryota	Sar	Y
EP00192	EukProt	Y	Tetraselmis astigmatica	1074897	Eukaryota	Viridiplantae	N
EP00198	EukProt	Y	Chlamydomonas reinhardtii	3055	Eukaryota	Viridiplantae	Y
EP00202	EukProt	Y	Volvox carteri	3067	Eukaryota	Viridiplantae	Y
EP00206	EukProt	Y	Coccomyxa subellipsoidea	248742	Eukaryota	Viridiplantae	Y
EP00210	EukProt	Y	Caulerpa lentillifera	148947	Eukaryota	Viridiplantae	N
EP00211	EukProt	Y	Chloroparvula japonica	1411623	Eukaryota	Viridiplantae	N
EP00217	EukProt	Y	Crustomastix stigmatica	195967	Eukaryota	Viridiplantae	N
EP00218	EukProt	Y	Dolichomastix tenuilepis	195968	Eukaryota	Viridiplantae	N
EP00229	EukProt	Y	Micromonas commoda	296587	Eukaryota	Viridiplantae	Y
EP00236	EukProt	Y	Picocystis salinarum	88271	Eukaryota	Viridiplantae	N
EP00240	EukProt	Y	Pyramimonas obovata	1411642	Eukaryota	Viridiplantae	Y
EP00241	EukProt	Y	Pyramimonas parkeae	36894	Eukaryota	Viridiplantae	N
EP00246	EukProt	Y	Nothoceros aenigmaticus	13813	Eukaryota	Viridiplantae	Y
EP00247	EukProt	Y	Physcomitrella patens	3218	Eukaryota	Viridiplantae	Y
EP00249	EukProt	Y	Psilotum nudum	3240	Eukaryota	Viridiplantae	Y
EP00250	EukProt	Y	Pteridium aquilinum	32101	Eukaryota	Viridiplantae	Y
EP00252	EukProt	Y	Welwitschia mirabilis	3377	Eukaryota	Viridiplantae	N
EP00255	EukProt	Y	Erythranthe guttata	4155	Eukaryota	Viridiplantae	N
EP00256	EukProt	Y	Glycine max	3847	Eukaryota	Viridiplantae	Y
EP00260	EukProt	Y	Arabidopsis thaliana	3702	Eukaryota	Viridiplantae	Y

Accession	Data source	Available proteome	Species name	NCBI taxid	Supergroup	Subgroup	In time-resolved species tree
EP00265	EukProt	Y	Oryza sativa	4530	Eukaryota	Viridiplantae	Y
EP00267	EukProt	Y	Zea mays	4577	Eukaryota	Viridiplantae	Y
EP00269	EukProt	Y	Selaginella moellendorffii	88036	Eukaryota	Viridiplantae	N
EP00271	EukProt	Y	Cylindrocystis brebissonii	102167	Eukaryota	Viridiplantae	N
EP00738	EukProt	Y	Chloropicon primus	1764295	Eukaryota	Viridiplantae	N
EP00793	EukProt	Y	Prasinoderma coloniale	156133	Eukaryota	Viridiplantae	N
EP00799	EukProt	Y	Chlamydomonas eustigma	1157962	Eukaryota	Viridiplantae	N
EP00850	EukProt	Y	Spirogloea muscicola	2576442	Eukaryota	Viridiplantae	N
EP00852	EukProt	Y	Chlamydomonas schloesseri	2026947	Eukaryota	Viridiplantae	N
EP00883	EukProt	Y	Tetradesmus obliquus	3088	Eukaryota	Viridiplantae	N
EP00886	EukProt	Y	Chlorella sorokiniana	3076	Eukaryota	Viridiplantae	N
EP00888	EukProt	Y	Micractinium conductrix	554055	Eukaryota	Viridiplantae	N
EP00890	EukProt	Y	Picochlorum soloecismus	2053932	Eukaryota	Viridiplantae	N
EP00893	EukProt	Y	Bathycoccus prasinos	41875	Eukaryota	Viridiplantae	N
EP00895	EukProt	Y	Klebsormidium nitens	105231	Eukaryota	Viridiplantae	Y
EP00896	EukProt	Y	Chara braunii	69332	Eukaryota	Viridiplantae	N
EP01024	EukProt	Y	Parvocaulis polyphysoides	219609	Eukaryota	Viridiplantae	N
EP01025	EukProt	Y	Chloroclados australasicus	35855	Eukaryota	Viridiplantae	N

Supplementary Table 3. Genes of the species tree concatenate

This table summaries the gene families used to produce species tree concatenates. For each gene family, the presence of key nodes in the single gene tree is indicated, as well as the trimmed alignment length (in the combined species tree concatenate). Additional columns identify the directory for its identification in the supplementary materials, as well as the presence of any human orthologs in the gene family.

LUCA	LACA	LBCA	nFECA	mFECA	LPCA	Approximate concatenate contribution (trimmed AA positions)	Identification folder	Human orthologs
**N**	Y	N	Y	N	N	325	AAA_25_C1_ORFs	RAD51
N	N	Y	N	Y	N	194	AAA_33_C6_ORFs	GUK1
N	N	N	N	Y	N	403	AFG1_ATPase_ORFs	AFG1L
N	N	Y	N	Y	N	318	CbiA_P5-26_ORFs	GET3
N	N	Y	N	Y	N	333	COX15-CtaA_ORFs	COX15
N	N	Y	N	Y	Y	330	CTP_transf_1_ORFs	CDS1
N	N	Y	N	Y	N	874	E1_dh_C1_ORFs	OGDH
Y	Y	Y	Y	Y	Y	407	GatB_N_ORFs	GATB
N	N	Y	N	Y	Y	568	GIDA_ORFs	MTO1
N	Y	N	Y	N	N	434	GTP_EFTU_D2_C7_ORFs	IF2G
N	N	Y	N	Y	Y	190	Methyltransf_4_ORFs	TRMB
N	N	Y	N	Y	Y	388	MnmE_helical_ORFs	GTPBP3
Y	Y	Y	Y	Y	Y	592	NFACT_N_ORFs	NEMF
N	Y	N	Y	N	N	57	Nop10p_ORFs	NOP10
N	N	Y	N	Y	Y	305	PCRF_C1_ORFs	MTRF1L
N	N	Y	N	Y	N	775	Phosphorylase_ORFs	PYGL
N	N	Y	N	Y	N	146	Polyketide_cyc_P1-4_ORFs	COQ10A
N	Y	N	Y	N	N	534	Radical_SAM_P8-21_ORFs	ELP3
Y	Y	Y	Y	Y	Y	150	Ribosom_S12_S23_ORFs	RS23
Y	Y	Y	Y	Y	Y	130	Ribosomal_L11_ORFs	MRPL11, RL12
N	N	Y	N	Y	Y	108	Ribosomal_L12_ORFs	MRPL12
Y	Y	Y	Y	Y	Y	119	Ribosomal_L14_ORFs	MRPL14, RL23
Y	Y	Y	Y	Y	Y	151	Ribosomal_L16_ORFs	MRPL16, RL10L
N	N	Y	N	Y	Y	106	Ribosomal_L19_ORFs	MRPL19
Y	Y	Y	Y	Y	Y	230	Ribosomal_L2_ORFs	MRPL2, RL8
N	N	Y	N	Y	Y	118	Ribosomal_L20_ORFs	MRPL20
N	Y	N	Y	N	N	149	Ribosomal_L21e_ORFs	RL21

LUCA	LACA	LBCA	nFECA	mFECA	LPCA	Approximate concatenate contribution (trimmed AA positions)	Identification folder	Human orthologs
N	Y	N	N	Y	Y	90	Ribosomal_L21p_ORFs	MRPL21
Y	Y	Y	Y	Y	Y	117	Ribosomal_L22_ORFs	MRPL22, RL17
N	Y	N	Y	N	N	118	Ribosomal_L26_ORFs	RL26
N	N	Y	N	Y	Y	101	Ribosomal_L28_ORFs	MRPL28
N	Y	N	Y	N	N	382	Ribosomal_L3_ORFs	RL3
N	Y	N	Y	N	N	120	Ribosomal_L32e_ORFs	RL32
N	Y	N	Y	N	N	89	Ribosomal_L37ae_ORFs	RL37A
N	N	N	Y	N	N	63	Ribosomal_L38e_ORFs	RL38
Y	Y	Y	Y	Y	Y	155	Ribosomal_L4_ORFs	MRPL4, RL4
N	Y	N	Y	N	N	87	Ribosomal_L44_ORFs	RL36A
Y	Y	Y	Y	Y	Y	103	Ribosomal_S11_ORFs	MRPS11, RS14
N	N	Y	N	Y	Y	110	Ribosomal_S16_ORFs	MRPS16
Y	Y	Y	Y	Y	Y	105	Ribosomal_S17_ORFs	MRPS17, RS11
Y	Y	Y	Y	Y	Y	124	Ribosomal_S19_ORFs	RS15
N	Y	N	Y	N	N	125	Ribosomal_S24e_ORFs	RS24
N	Y	N	Y	N	N	67	Ribosomal_S28e_ORFs	RS28
Y	Y	Y	Y	Y	Y	198	Ribosomal_S3_C_ORFs	RS3
Y	Y	Y	Y	Y	Y	176	Ribosomal_S5_ORFs	MRPS5, RS2
Y	Y	Y	Y	Y	Y	172	Ribosomal_S7_ORFs	MRPS7, RS5
Y	Y	Y	Y	Y	Y	127	Ribosomal_S8_ORFs	RS15A
Y	Y	Y	Y	Y	Y	100	Ribosomal_S9_ORFs	MRPS9, RS16
N	Y	N	Y	N	N	67	RNA_pol_N_ORFs	RPAB5
N	N	N	Y	N	N	261	RNase_P_p30_ORFs	RPP30
N	N	Y	N	Y	N	203	SCO1-SenC_ORFs	SCO1/2
Y	Y	Y	Y	Y	N	60	SecE_ORFs	SC61G
N	N	N	N	Y	N	251	SQS_PSY_C3_ORFs	NDUFAF6
Y	Y	Y	Y	Y	Y	392	SRP_SPB_ORFs	SRP54
Y	Y	Y	Y	Y	N	241	TIM_ORFs	TPIS
N	Y	N	Y	N	N	423	TRM_P1-2_ORFs	TRMT1
Y	Y	Y	Y	Y	N	383	tRNA-synt_2d_ORFs	SYFA
Y	Y	Y	Y	Y	N	357	TruB_C_2_ORFs	DKC1
Y	Y	Y	Y	Y	N	244	TsaD_ORFs	OSGEP/L1
N	N	Y	N	Y	Y	333	YchF-GTPase_C_ORFs	OLA1
Y	Y	Y	Y	Y	N	695	zf-C4_Topoisom_ORFs	TOP3A
Y	Y	Y	Y	Y	N	688	tRNA_synthFbeta_ORFs	SYFB

Supplementary Table 4. Species tree node age sensitivity tests.

This table summarises sensitivity tests exploring the effect of species tree topology on MCMCTree time resolution. These results can be found in the supplementary material, and each test condition matches a numbered folder (1-9). Time values are in Ga, with the average age and the 95% CI interval bracketed below, asterisked cells within a column are definition equivalent, where the node has an associated MCMCTree calibration it is bracketed beneath the node label, the set used for our subsequent gene tree analyses is highlighted in red. Species tree topology was found to produce similar ages for FECAs/LECA but more variability with deeper nodes, particularly LBCA. These results are more fully discussed in Supplementary Notes 3.

**MCMCTree folder**	1	2	3	4	5
**Bacterial root**	Unconstrained (synergistales / atribacteriales)	Constrained (gracilicute / terrabacteria)	Constrained (gracilicute / terrabacteria)	Constrained (gracilicute / terrabacteria)	Constrained (gracilicute / terrabacteria)
**Eukaryote root**	Opimoda / Diphoda	Opimoda / Diphoda	Metamonad	Opimoda / Diphoda	Metamonad
**Total Oxyphotobacteria calibration**	Not set	Not set	Not set	Set	Set
LUCA<br>B(33.470,45.200,1e-100,1e-100)	4.48<br>(4.41-4.54)	4.49<br>(4.42-4.51)	4.50<br>(4.43-4.52)	4.50<br>(4.43-4.52)	4.50<br>(4.43-4.52)
LACA	3.24<br>(3.13-3.34)	3.52<br>(3.36-3.67)	3.52<br>(3.39-3.67)	3.60<br>(3.45-3.76)	3.53<br>(3.39-3.67)
LBCA<br>B(32.25,45.200,1e-100,1e-100)	3.24<br>(32.3-3.26)	3.23*<br>(3.23-3.24)	3.34*<br>(3.31-3.39)	3.23*<br>(3.23-3.24)	3.35*<br>(3.32-3.40)
Gracilicute /Terrabacteria split	2.49<br>(2.39-2.56)	3.23*<br>(3.23-3.24)	3.34*<br>(3.31-3.39)	3.23*<br>(3.23-3.24)	3.35*<br>(3.32-3.40)
Crown Oxyphotobacteria<br>B(20.136,34.480,0.01,0.01)	1.63<br>(1.62-1.66)	1.64<br>(1.53-1.76)	1.65<br>(1.53-1.78)	1.96<br>(1.84-2.04)	2.00<br>(1.92-2.08)
Total Oxyphotobacteria<br>B(32.25,45.200,1e-100,1e-100)	2.08<br>(2.13-2.02)	2.69<br>(2.54-2.84)	3.23<br>(3.23-3.24)	2.61<br>(2.44-2.81)	3.23<br>(3.23-3.24)
Nuclear FECA	2.63<br>(2.52-2.72)	2.85<br>(2.70-2.97)	2.85<br>(2.74-2.98)	2.92<br>(2.79-3.05)	2.86<br>(2.74-2.99)
Mitochondrial FECA	1.96<br>(1.88-2.03)	2.19<br>(2.08-2.19)	2.22<br>(2.14-2.30)	2.24<br>(2.13-2.37)	2.38<br>(2.28-2.47)
LECA<br>B(10.856,18.796,0.01,0.01)	1.57<br>(1.53-1.61)	1.69<br>(1.63-1.75)	1.88<br>(1.85-1.92)	1.74<br>(1.67-1.80)	2.23<br>(2.14-2.32)
Total-group plastids	1.49<br>(1.40-1.59)	1.51<br>(1.42-1.60)	1.49<br>(1.41-1.60)	1.64<br>(1.54-1.77)	1.60<br>(1.49-1.74)
Crown plastids	1.38<br>(1.30-1.46)	1.43<br>(1.35-1.51)	1.41<br>(1.33-1.49)	1.53<br>(1.44-1.63)	1.46<br>(1.37-1.58)

Supplementary Table 5. Applying test constraints to key species tree nodes.

This table summarises the results of sensitivity tests exploring the effect of changing the ages of key nodes within the species tree, and the propagated effect to other key nodes under time resolution with MCMCTree. Node ages are presented in Ga, with the 95% CI interval bracketed below. Where a key node is constrained by a calibration, the MCMCTree format of calibration is bracketed beneath). In all tests LECA had similar values, as well as younger nodes within eukaryotes, changing the values of deeper nodes can influence the ages of m/nFECA, although where the test allows we find the branch length from m/nFECAs to LECA to be unequal. Further discussion of these results is given in Supplementary Notes 3.

**MCMCTree folder**	4	6	7	8	9
**Test**	Control (calibration set used to resolving gene trees)	LACA=LBCA	nFECA=mFECA	LBCA=>4Ga	nFECA sister to Hodarchaeales
**Bacterial root**	Constrained (gracilicute / terrabacteria)	Constrained (gracilicute / terrabacteria)	Constrained (gracilicute / terrabacteria)	Constrained (gracilicute / terrabacteria)	Constrained (gracilicute / terrabacteria)
**Eukaryote root**	Opimoda / Diphoda	Opimoda / Diphoda	Opimoda / Diphoda	Opimoda / Diphoda	Opimoda / Diphoda
**Total Oxyphotobacteria calibration**	Set	Not set	Not set	Not set	Not set
LUCA<br>B(33.470,45.200,1e-100,1e-100)	4.50<br>(4.43-4.52)	4.48<br>(4.40-4.52)	4.49<br>(4.41-4.52)	4.50<br>(4.46-4.52)	4.50<br>(4.43-4.52)
LACA	3.60<br>(3.45-3.76)	3.23*<br>(3.23-3.25)	3.38<br>(3.23-3.53)	3.42<br>(3.26-3.61)	3.68<br>(3.53-3.86)
LBCA<br>B(32.25,45.200,1e-100,1e-100)	3.23<br>(3.23-3.24)	3.23*<br>(3.23-3.25)	3.23<br>(3.23-3.25)	4.03*<br>(4.03-4.05)	3.23<br>(3.23-3.24)
Crown oxyphotobacteria<br>B(20.136,34.480,0.01,0.01)	1.96<br>(1.84-2.04)	1.94<br>(1.81-2.03)	1.94<br>(1.81-2.03)	1.92<br>(1.78-2.03)	1.97<br>(1.85-2.04)
Total Oxyphotobacteria<br>B(32.25,45.200,1e-100,1e-100)	2.61<br>(2.44-2.81)	2.56<br>(2.39-2.78)	2.52<br>(2.38-2.52)	2.46<br>(2.31-2.63)	2.63<br>(2.46-2.83)
Nuclear FECA	2.92<br>(2.79-3.05)	2.70<br>(2.60-2.78)	2.53*<br>(2.45-2.62)	2.80<br>(2.65-2.98)	2.73<br>(2.60-2.88)
Mitochondrial FECA	2.24<br>(2.13-2.24)	2.16<br>(2.06-2.27)	2.53*<br>(2.45-2.62)	2.16<br>(2.02-2.35)	2.24<br>(2.13-2.34)
LECA<br>B(10.856,18.796,0.01,0.01)	1.74<br>(1.67-1.80)	1.67<br>(1.62-1.73)	1.72<br>(1.66-1.77)	1.69<br>(1.61-1.78)	1.73<br>(1.68-1.80)
Total-group plastids	1.64<br>(1.54-1.77)	1.59<br>(1.49-1.71)	1.62<br>(1.52-1.74)	1.60<br>(1.47-1.74)	1.65<br>(1.53-1.76)
Crown plastids	1.53<br>(1.44-1.63)	1.48<br>(1.39-1.58)	1.51<br>(1.42-1.60)	1.49<br>(1.38-1.61)	1.53<br>(1.44-1.64)

Supplementary Table 6. Fossil calibrations.

This table summarises calibrations used in and to evaluate the species tree, literature justification for these calibrations is given in the supplementary material. All used MCMCTREE calibrations are in the form of a uniform density distribution (prefix ‘B’) as understood by MCMCTREE, and contain four values defining the minimum and maximum ages (in x 100My) and their associated calibration bounds. In our analysis we simplified these boundaries to one of two states, a soft boundary of 0.01 or a hard boundary of 1e-100. Where these calibrations are found more than once in the tree they are assigned a unique cross brace number (prefixed with ‘#’). Three additional calibrations (indicated by *) were checked against the finished tree, though were not placed as calibrations within the species tree.

Fossil Calibration	Min Age (My)	Max Age (My)	Min Soft / Hard	Max Soft / Hard	MCMCTREE uniform calibration /	Tree instances	MCMCTREE node number
LUCA	3347	4520	hard	hard	B(33.470,45.200,1e-100,1e-100)	x1	456
Crown Bacteria	3225	4520	hard	hard	B(32.25,45.200,1e-100,1e-100)	x1	629
Total Oxyphotobacteria	3225	4520	hard	hard	B(32.25,45.200,1e-100,1e-100)	x1	633
Crown Oxyphotobacteria	2014	3448	soft	soft	B(20.136,34.480,0.01,0.01)	x1	634
Total Eukaryota	1619	4520	soft	hard	B(16.191,45.200,0.01,1e-100)	x1	463
Crown Chlamydiia	1086	1880	soft	soft	B(10.856,18.796,0.01,0.01)	x1*	895
Crown Eukaryota	1086	1880	soft	soft	[#1 B{10.856,18.796,0.01,0.01}]	x2	464/715
Crown Archaeplastida	1030	1880	soft	hard	[#2 B{10.300,18.796,0.01,1e-100}]	x2	521/772
Rhodophyta	1030	1880	hard	hard	[#19 B{10.300,18.796,1e-100,1e-100}]	x1	652
Viridiplantae/Chloroplastida (Chlorophyta - Streptophyta)	940	1880	hard	hard	[#3 B{9.404,18.796,1e-100,1e-100}]	x3	523/774
Crown Opisthokonta	879	1880	hard	hard	[#4 B{8.790,18.796,1e-100,1e-100}]	X2*	468/719
Total-group Eumycota (Spizellomyces – Saccharomyces)	879	1880	hard	soft	[#5 B{8.790,18.796,1e-100,0.01}]	x2	503/754
Metazoa	574	609	hard	soft	[#6 B{5.740,6.090,1e-100,0.01}]	x2	471/722
Bilateria/Nephrozoa	532	591	hard	soft	[#7 B{5.320,5.908,1e-100,0.01}]	x2*	473/724
Crown Protostomia (Lottia-Limulus)	532	591	hard	soft	[#8 B{5.320,5.908,1e-100,0.01}]	x2	485/736
Mollusca-Brachiopod+Annelida	532	591	hard	soft	[#9 B{5.320,5.908,1e-100,0.01}]	x2	491/742
Mollusca	532	549	soft	soft	[#10 B{5.32,5.49,0.01,0.01}]	x2	494/745
Deuterostomia (Strongylocentrotus-Mouse+Zebrafish)	517	591	hard	soft	[#11 B{5.173,5.908,1e-100,0.01}]	x2	474/725
Mandibulata (Insect-Chelicerate)	514	543	hard	soft	[#12 B{5.140,5.430,1e-100,0.01}]	x2	486/737
Echinoderms	509	549	soft	soft	[#13 B{5.09,5.49,0.01,0.01}]	x2	484/735

Fossil Calibration	Min Age (My)	Max Age (My)	Min Soft / Hard	Max Soft / Hard	MCMCTREE uniform calibration /	Tree instances	MCMCTREE node number
Annelida	477	636	soft	soft	[#14 B{4.77,6.36,0.01,0.01}]	x2	492/743
Embryophyta<br>(Bryophyta – Tracheophyta)	431	516	hard	soft	[#15 B{4.305,5.155,1e-100,0.01}]	x3	525/776
Osteichthyes<br>(Mouse-Zebrafish)	421	445	hard	soft	[#16 B{4.207,4.449,1e-100,0.01}]	x2	475/726
Dikarya	189	453	soft	soft	[#17 B{1.891,4.527,0.01,0.01}]	x2	504/755
Angiosperms	126	247	soft	soft	[#18 B{1.259,2.473,0.01,0.01}]	x3	527/778

Supplementary Table 7. Our investigated gene families and their duplication dates

This table presents the gene families we were able to time resolve in our investigation. The initial two columns specify the database source of the HMM utilized in the pipeline search. If the HMM was constructed, the reference protein used for its creation is provided in the subsequent column, with two exceptions explained below. The third column indicates whether all search results were used ("whole"), or if specific subtrees, clusters, or sets of clusters ("Paths," see Supplementary Notes 1) from DO pipeline analysis. The fourth column presents the tree reconciliation method employed ("treerecs," Supplementary Notes 1) and column five the node index number for key nodes in the DA analysis (mappable to MCMCTree reports in the Supplementary Data). Dates for these nodes are expressed in Ga in columns 6-9, the mean age is conditionally formatted on duplication and prokaryote divergence nodes on a color scale ranging from nFECA (red), through mitochondrial endosymbiosis (white), to LECA (blue). The origin of each gene family is indicated: "Alpha" denotes Alphaproteobacterial descent, and "Asgard" signifies descent from Asgard archaea. Gene names and NCBI gene IDs are provided for any human orthologs in the descendent LECA nodes, along with the occurrence of this gene in any manuscript figures. An expanded version of this table, including additional columns such as subcellular localization patterns and corresponding analysis folder names, is available in the Supplementary Data.

The exceptions to our default search strategy:

1. Was generated from a PSIBLAST iterated search of an asgard archaeal SNARE protein [34]
2. These results were pooled from two separate HMMs, PFAM PF00125 and PF00538

Source	HMM or Reference protein	Data type	treerecs	Node	Node type	mean age	CI low	CI high		source	protein name	NCBI gene id	Figure occurrence
PFAM	PF00198	Cluster	2	187	mFECA divergence	2.24	2.24	2.24		Alpha.	-	-	
PFAM	PF00198	Cluster	2	188	Duplication	1.97	1.81	2.14		Alpha.	-	-	
PFAM	PF00198	Cluster	2	189	LECA	1.73	1.67	1.80		Alpha.	PDHX (E3BP)	8050	4
PFAM	PF00198	Cluster	2	223	LECA	1.72	1.67	1.79		Alpha.	DLAT (E2)	1737	4
PFAM	PF05761	Whole	2	110	BACT divergence	2.25	1.99	2.56		Bacterial	-	-	

PFAM	PF05761	Whole	2	111	Duplication	2.02	1.83	2.28		Bacterial	-	-	
PFAM	PF05761	Whole	2	112	LECA	1.75	1.68	1.80		Bacterial	NT5C2	22978	
PFAM	PF05761	Whole	2	154	LECA	1.74	1.68	1.80		Bacterial	NT5DC3	51559<br>64943	
PFAM	PF02096	Whole	1	134	BACT divergence	2.35	2.08	2.67		Bacterial	-	-	
PFAM	PF02096	Whole	1	135	Duplication	1.91	1.78	2.13		Bacterial	-	-	
PFAM	PF02096	Whole	1	182	LECA	1.73	1.67	1.80		Bacterial	COX18	285521	4
PFAM	PF02096	Whole	1	136	LECA	1.75	1.68	1.80		Bacterial	OXA1L	5018	4
Custom HMM	A8K401	Whole	1	218	BACT divergence	2.16	1.97	2.38		Bacterial	-	-	
Custom HMM	A8K401	Whole	1	219	Duplication	1.92	1.80	2.08		Bacterial	-	-	
Custom HMM	A8K401	Whole	1	306	LECA	1.75	1.63	1.89		Bacterial	PHB1	5245	
Custom HMM	A8K401	Whole	1	220	LECA	1.83	1.68	2.00		Bacterial	PHB2	11331	
PFAM	PF13671	Cluster	2	114	nFECA divergence	2.92	2.92	2.92		Asgard.	-	-	
PFAM	PF13671	Cluster	2	115	Duplication	2.56	2.07	2.90		Asgard.	-	-	
PFAM	PF13671	Cluster	2	177	LECA	1.77	1.71	1.83		Asgard.	PSTK	118672	SF6
PFAM	PF13671	Cluster	2	116	LECA	1.78	1.71	1.83		Asgard.	-	-	
PFAM	PF07728	Cluster	2	489	nFECA divergence	2.92	2.92	2.92		Asgard.	-	-	
PFAM	PF07728	Cluster	2	490	Duplication	2.82	2.73	2.88		Asgard.	-	-	

PFAM	PF07728	Cluster	2	804	Duplication	2.74	2.58	2.85		Asgard.	-	-	
PFAM	PF07728	Cluster	2	805	LECA	1.79	1.73	1.83		Asgard.	MCM2	4171	SF2
PFAM	PF07728	Cluster	2	870	Duplication	2.48	2.28	2.66		Asgard.	-	-	
PFAM	PF07728	Cluster	2	914	LECA	1.78	1.71	1.83		Asgard.	MCM9	254394	SF2
PFAM	PF07728	Cluster	2	871	LECA	1.78	1.71	1.83		Asgard.	MCM8	84515	SF2
PFAM	PF07728	Cluster	2	491	Duplication	2.71	2.58	2.82		Asgard.	-	-	
PFAM	PF07728	Cluster	2	679	Duplication	2.52	2.30	2.71		Asgard.	-	-	
PFAM	PF07728	Cluster	2	745	LECA	1.79	1.72	1.83		Asgard.	MCM5	4174	SF2
PFAM	PF07728	Cluster	2	680	LECA	1.81	1.75	1.83		Asgard.	MCM6	4175	SF2
PFAM	PF07728	Cluster	2	492	Duplication	2.59	2.43	2.74		Asgard.	-	-	
PFAM	PF07728	Cluster	2	493	LECA	1.79	1.72	1.83		Asgard.	MCM4	4173	SF2
PFAM	PF07728	Cluster	2	554	Duplication	2.42	2.21	2.61		Asgard.	-	-	
PFAM	PF07728	Cluster	2	555	LECA	1.79	1.72	1.83		Asgard.	MCM3	4172	SF2
PFAM	PF07728	Cluster	2	615	LECA	1.81	1.75	1.83		Asgard.	MCM7	4176	SF2
PFAM	PF07728	Cluster	2	170	nFECA divergence	2.92	2.92	2.92		Asgard.	-	-	
PFAM	PF07728	Cluster	2	171	Duplication	2.62	2.37	2.81		Asgard.	-	-	
PFAM	PF07728	Cluster	2	293	partial LECA	1.77	1.71	1.82		Asgard.	-	-	
PFAM	PF07728	Cluster	2	172	Duplication	2.38	2.13	2.63		Asgard.	-	-	
PFAM	PF07728	Cluster	2	173	LECA	1.79	1.72	1.83		Asgard.	ATAD1	84896	4
PFAM	PF07728	Cluster	2	223	Duplication	1.92	1.80	2.14		Asgard.	-	-	
PFAM	PF07728	Cluster	2	264	LECA	1.77	1.71	1.82		Asgard.	FIGN	55137	4

PFAM	PF07728	Cluster	2	224	LECA	1.78	1.71	1.83		Asgard.	SPAST	6683	4
PFAM	PF12774	Cluster	1	303	nFECA divergence	2.92	2.92	2.92		Asgard.	-	-	
PFAM	PF12774	Cluster	1	304	Duplication	2.72	2.50	2.88		Asgard.	-	-	
PFAM	PF12774	Cluster	1	452	LECA	1.78	1.71	1.82		Asgard.	RFC2	5982	SF2
PFAM	PF12774	Cluster	1	305	Duplication	2.54	2.28	2.76		Asgard.	-	-	
PFAM	PF12774	Cluster	1	306	LECA	1.80	1.74	1.83		Asgard.	RFC4	5984	SF2
PFAM	PF12774	Cluster	1	376	LECA	1.80	1.73	1.83		Asgard.	RFC5	5985	SF2
PFAM	PF06472	Whole	2	153	mFECA divergence	2.24	2.24	2.24		Alpha.	-	-	
PFAM	PF06472	Whole	2	154	Duplication	2.07	1.97	2.15		Alpha.	-	-	
PFAM	PF06472	Whole	2	259	partial LECA	1.81	1.52	2.01		Alpha.	ABCD4	5826	
PFAM	PF06472	Whole	2	155	Duplication	1.90	1.82	2.00		Alpha.	-	-	
PFAM	PF06472	Whole	2	238	LECA	1.75	1.68	1.80		Alpha.	-	-	
PFAM	PF06472	Whole	2	156	Duplication	1.82	1.75	1.91		Alpha.	-	-	
PFAM	PF06472	Whole	2	199	LECA	1.72	1.67	1.78		Alpha.	ABCD3	5825	
PFAM	PF06472	Whole	2	157	LECA	1.70	1.67	1.76		Alpha.	ABCD1/2	215 225	
PFAM	PF00005	Cluster subtree	2	227	mFECA divergence	2.24	2.24	2.24		Alpha.	-	-	
PFAM	PF00005	Cluster subtree	2	228	Duplication	2.05	1.94	2.15		Alpha.	-	-	
PFAM	PF00005	Cluster subtree	2	229	LECA	1.69	1.67	1.74		Alpha.	ABCB1/5/11	8647 340273 5243 5244	4

PFAM	PF00005	Cluster subtree	2	293	Duplication	1.95	1.84	2.06		Alpha.	-	-	
PFAM	PF00005	Cluster subtree	2	294	LECA	1.75	1.68	1.80		Alpha.	ABCB2/9	6890 23457	4
PFAM	PF00005	Cluster subtree	2	328	LECA	1.78	1.72	1.80		Alpha.	ABCB8/10	23456 11194	4
PFAM	PF00005	Cluster subtree	2	154	mFECA divergence	2.30	2.30	2.30		Alpha.	-	-	
PFAM	PF00005	Cluster subtree	2	155	Duplication	2.20	2.05	2.28		Alpha.	-	-	
PFAM	PF00005	Cluster subtree	2	156	LECA	1.78	1.71	1.83		Alpha.	ABCB6	10058	4
PFAM	PF00005	Cluster subtree	2	195	LECA	1.76	1.71	1.82		Alpha.	ABCB7	22	4
PFAM	PF00005	Cluster	2	257	mFECA divergence	2.24	2.24	2.24		Alpha.	-	-	
PFAM	PF00005	Cluster	2	258	Duplication	2.04	1.96	2.12		Alpha.	-	-	
PFAM	PF00005	Cluster	2	394	LECA	1.78	1.72	1.80		Alpha.	ABCF3	55324	
PFAM	PF00005	Cluster	2	259	Duplication	1.95	1.87	2.03		Alpha.	-	-	
PFAM	PF00005	Cluster	2	260	LECA	1.71	1.67	1.78		Alpha.	ABCF1	23	
PFAM	PF00005	Cluster	2	314	LECA	1.72	1.67	1.79		Alpha.	ABCF2	10061	
PFAM	PF00155	Path	1	183	BACT divergence	2.20	1.97	2.52		Bacterial	-	-	
PFAM	PF00155	Path	1	184	Duplication	1.90	1.79	2.06		Bacterial	-	-	
PFAM	PF00155	Path	1	267	LECA	1.75	1.68	1.80		Bacterial	GOT2	2806	4
PFAM	PF00155	Path	1	185	LECA	1.74	1.68	1.80		Bacterial	GOT1L1	137362	4

PFAM	PF03029	Whole	2	295	nFECA divergence	2.92	2.92	2.92		Asgard.	-	-	
PFAM	PF03029	Whole	2	301	Duplication	2.64	2.42	2.81		Asgard.	-	-	
PFAM	PF03029	Whole	2	451	LECA	1.74	1.67	1.80		Asgard.	GPN1	11321	SF3
PFAM	PF03029	Whole	2	302	Duplication	2.20	1.99	2.43		Asgard.	-	-	
PFAM	PF03029	Whole	2	380	LECA	1.74	1.68	1.80		Asgard.	GPN2	54707	SF3
PFAM	PF03029	Whole	2	303	LECA	1.77	1.69	1.80		Asgard.	GPN3	51184	SF3
PFAM	PF00137	Whole	2	221	nFECA divergence	2.92	2.92	2.92		Asgard.	-	-	
PFAM	PF00137	Whole	2	222	Duplication	2.35	2.01	2.68		Asgard.	-	-	
PFAM	PF00137	Whole	2	223	LECA	1.75	1.68	1.80		Asgard.	ATP6V0B	533	
PFAM	PF00137	Whole	2	303	LECA	1.76	1.70	1.80		Asgard.	ATP6V0C	527	
Custom HMM	B2CQT6	Whole	2	178	BACT divergence	2.34	2.08	2.63		Bacterial	-	-	
Custom HMM	B2CQT6	Whole	2	179	Duplication	1.96	1.82	2.16		Bacterial	-	-	
Custom HMM	B2CQT6	Whole	2	180	LECA	1.72	1.67	1.79		Bacterial	SLC15A3/4/5	121260 51296 729025	4
Custom HMM	B2CQT6	Whole	2	254	LECA	1.72	1.67	1.79		Bacterial	SLC15A1/2	6565 6564	4
Custom HMM	B7Z7E6	Whole	2	122	ARCH divergence	2.53	2.23	2.91		Archaeal	-	-	
Custom HMM	B7Z7E6	Whole	2	123	Duplication	1.94	1.83	2.09		Archaeal	-	-	
Custom HMM	B7Z7E6	Whole	2	198	LECA	1.75	1.68	1.80		Archaeal	CARS2	79587	4

Custom HMM	B7Z7E6	Whole	2	124	LECA	1.77	1.70	1.80		Archaeal	CARS1	833	4
PFAM	PF01027	Subtree	1	150	BACT divergence	2.59	2.23	2.91		Bacterial	-	-	
PFAM	PF01027	Subtree	1	151	Duplication	1.95	1.79	2.20		Bacterial	-	-	
PFAM	PF01027	Subtree	1	228	partial LECA	1.73	1.67	1.80		Bacterial	TMBIM4	51643	
PFAM	PF01027	Subtree	1	152	LECA	1.74	1.67	1.80		Bacterial	TMBIM1,GRINA,FAIM2	2907 64114 23017	
PFAM	PF01027	Subtree	2	153	mFECA divergence	2.24	2.24	2.24		Alpha.	-	-	
PFAM	PF01027	Subtree	2	154	Duplication	2.04	1.92	2.14		Alpha.	-	-	
PFAM	PF01027	Subtree	2	214	LECA	1.73	1.67	1.80		Alpha.	GHITM	27069	
PFAM	PF01027	Subtree	2	155	LECA	1.73	1.67	1.80		Alpha.	TMBIM6	7009	
PFAM	PF09079	Whole	1	179	ARCH divergence	2.54	2.22	2.92		Archaeal	-	-	
PFAM	PF09079	Whole	1	180	Duplication	1.99	1.76	2.35		Archaeal	-	-	
PFAM	PF09079	Whole	1	181	partial LECA	1.59	1.24	1.97		Archaeal	ORC1	4998	SF2
PFAM	PF09079	Whole	1	214	LECA	1.75	1.68	1.80		Archaeal	CDC6	990	SF2
PFAM	PF01633	Whole	1	135	mFECA divergence	2.24	2.24	2.24		Alpha.	-	-	
PFAM	PF01633	Whole	1	136	Duplication	1.91	1.78	2.11		Alpha.	-	-	
PFAM	PF01633	Whole	1	210	LECA	1.73	1.67	1.80		Alpha.	CHKA/B	1119 1120	

PFAM	PF01633	Whole	1	137	LECA	1.73	1.67	1.80		Alpha.	ETNK1/2	55500<br>55224	
PFAM	PF17876	Whole	1	167	BACT divergence	2.64	2.41	2.90		Bacterial	-	-	
PFAM	PF17876	Whole	1	168	Duplication	1.89	1.79	2.02		Bacterial	-	-	
PFAM	PF17876	Whole	1	224	partial LECA	1.02	0.86	1.16		Bacterial	DIS3L	115752	SF3
PFAM	PF17876	Whole	1	169	LECA	1.72	1.67	1.79		Bacterial	DIS3	22894	SF3
PFAM	PF01866	Whole	2	183	nFECA divergence	2.92	2.92	2.92		Asgard.	-	-	
PFAM	PF01866	Whole	2	184	Duplication	2.45	2.19	2.68		Asgard.	-	-	
PFAM	PF01866	Whole	2	269	LECA	1.75	1.68	1.80		Asgard.	DPH2	1802	SF6
PFAM	PF01866	Whole	2	185	LECA	1.73	1.67	1.80		Asgard.	DPH1	1801	SF6
PFAM	PF03764	Whole	2	271	nFECA divergence	2.92	2.92	2.92		Asgard.	-	-	
PFAM	PF03764	Whole	2	272	Duplication	2.44	2.17	2.69		Asgard.	-	-	
PFAM	PF03764	Whole	2	273	LECA	1.79	1.75	1.81		Asgard.	EFTUD2	9343	SF6
PFAM	PF03764	Whole	2	367	LECA	1.78	1.74	1.80		Asgard.	EEF2	1938	SF6
PFAM	PF01176	Whole	2	209	nFECA divergence	2.92	2.92	2.92		Asgard.	-	-	
PFAM	PF01176	Whole	2	210	Duplication	2.54	2.08	2.87		Asgard.	-	-	
PFAM	PF01176	Whole	2	211	LECA	1.76	1.69	1.80		Asgard.	EIF1AD	84285	SF6
PFAM	PF01176	Whole	2	273	LECA	1.75	1.68	1.80		Asgard.	EIF1AX	1964	SF6
PFAM	PF01873	Whole	2	250	nFECA divergence	2.92	2.92	2.92		Asgard.	-	-	

PFAM	PF01873	Whole	2	251	Duplication	2.55	2.24	2.81		Asgard.	-	-	
PFAM	PF01873	Whole	2	252	LECA	1.78	1.72	1.80		Asgard.	EIF2S2	8894	SF6
PFAM	PF01873	Whole	2	350	LECA	1.77	1.73	1.80		Asgard.	EIF5	1983	SF6
PFAM	PF02732	Whole	2	235	nFECA divergence	2.92	2.92	2.92		Asgard.	-	-	
PFAM	PF02732	Whole	2	236	Duplication	2.46	2.18	2.73		Asgard.	-	-	
PFAM	PF02732	Whole	2	237	LECA	1.73	1.67	1.80		Asgard.	ERCC4	2072	
PFAM	PF02732	Whole	2	314	Duplication	2.15	1.92	2.45		Asgard.	-	-	
PFAM	PF02732	Whole	2	315	LECA	1.73	1.67	1.80		Asgard.	EME1/2	146956 197342	
PFAM	PF02732	Whole	2	355	LECA	1.75	1.68	1.80		Asgard.	MUS81	80198	
PFAM	PF04410	Whole	2	188	nFECA divergence	2.92	2.92	2.92		Asgard.	-	-	
PFAM	PF04410	Whole	2	189	Duplication	2.35	2.01	2.69		Asgard.	-	-	
PFAM	PF04410	Whole	2	190	LECA	1.74	1.68	1.80		Asgard.	NAF1	92345	SF3
PFAM	PF04410	Whole	2	252	LECA	1.74	1.67	1.80		Asgard.	GAR1	54433	SF3
PFAM	PF00626	Whole	2	226	nFECA divergence	2.92	2.92	2.92		Asgard.	-	-	
PFAM	PF00626	Whole	2	227	Duplication	2.57	2.33	2.78		Asgard.	-	-	
PFAM	PF00626	Whole	2	341	Duplication	1.98	1.81	2.23		Asgard.	-	-	
PFAM	PF00626	Whole	2	387	LECA	1.76	1.69	1.80		Asgard.	SEC24A	10802	3
PFAM	PF00626	Whole	2	342	LECA	1.73	1.67	1.80		Asgard.	SEC24C/D	9632 9871	3
PFAM	PF00626	Whole	2	228	LECA	1.76	1.70	1.80		Asgard.	SEC23A/B	10483 10484	3

PFAM	PF03143	Whole	2	275	nFECA divergence	2.92	2.92	2.92		Asgard.		-	
PFAM	PF03143	Whole	2	276	Duplication	2.73	2.51	2.87		Asgard.		-	
PFAM	PF03143	Whole	2	411	LECA	1.74	1.68	1.80		Asgard.	EEF1A1/2	1917 1915	SF6
PFAM	PF03143	Whole	2	277	Duplication	2.61	2.37	2.79		Asgard.		-	
PFAM	PF03143	Whole	2	389	LECA	1.76	1.68	1.80		Asgard.		-	
PFAM	PF03143	Whole	2	278	Duplication	2.27	2.03	2.52		Asgard.		-	
PFAM	PF03143	Whole	2	279	LECA	1.77	1.71	1.80		Asgard.	HBS1L	10767	SF6
PFAM	PF03143	Whole	2	316	LECA	1.78	1.73	1.81		Asgard.	GSPT1/2	23708 2935	SF6
PFAM	PF04670	Whole	2	146	nFECA divergence	2.92	2.92	2.92		Asgard.	-	-	
PFAM	PF04670	Whole	2	147	Duplication	2.78	2.46	2.91		Asgard.	-	-	
PFAM	PF04670	Whole	2	220	LECA	1.76	1.69	1.80		Asgard.	RRAGC/D	64121 58528	
PFAM	PF04670	Whole	2	148	LECA	1.74	1.68	1.80		Asgard.	RRAGA/B	10670 10325	
Custom HMM	1*	Whole	2	501	nFECA divergence	2.92	2.92	2.92		Asgard.	-	-	
Custom HMM	1*	Whole	2	502	Duplication	2.85	2.71	2.91		Asgard.	-	-	
Custom HMM	1*	Whole	2	503	Duplication	2.36	1.95	2.75		Asgard.	-	-	
Custom HMM	1*	Whole	2	565	LECA	2.05	1.49	2.57		Asgard.	-	-	
Custom HMM	1*	Whole	2	504	LECA	1.74	1.67	1.80		Asgard.	VTI1A/B	143187 10490	3

Custom HMM	1*	Whole	2	585	Duplication	2.76	2.56	2.89		Asgard.	-	-	
Custom HMM	1*	Whole	2	586	Duplication	2.54	2.18	2.80		Asgard.	-	-	
Custom HMM	1*	Whole	2	676	partial LECA	2.32	1.84	2.70		Asgard.	-	-	
Custom HMM	1*	Whole	2	587	Duplication	2.20	1.85	2.62		Asgard.	-	-	
Custom HMM	1*	Whole	2	588	LECA	1.75	1.68	1.80		Asgard.	STX8	9482	3
Custom HMM	1*	Whole	2	625	LECA	1.76	1.69	1.80		Asgard.	STX6/10	8677 10228	3
Custom HMM	1*	Whole	2	692	Duplication	2.64	2.38	2.83		Asgard.	-	-	
Custom HMM	1*	Whole	2	693	LECA	1.75	1.68	1.80		Asgard.	STX5	6811	3
Custom HMM	1*	Whole	2	758	Duplication	2.51	2.22	2.76		Asgard.	-	-	
Custom HMM	1*	Whole	2	759	LECA	1.77	1.70	1.80		Asgard.	STX16	8675	3
Custom HMM	1*	Whole	2	825	Duplication	2.34	2.04	2.63		Asgard.	-	-	
Custom HMM	1*	Whole	2	914	LECA	1.73	1.67	1.80		Asgard.	STX7/12	8417 23673	3
Custom HMM	1*	Whole	2	826	LECA	1.74	1.68	1.80		Asgard.	STX1B/2/4	112755 6810 2054 6804	3
PFAM	PF13871	Whole	1	76	mFECA divergence	2.24	2.24	2.24		Alpha.	-	-	
PFAM	PF13871	Whole	1	77	Duplication	1.93	1.81	2.07		Alpha.	-	-	
PFAM	PF13871	Whole	1	120	LECA	1.73	1.67	1.80		Alpha.	-	-	
PFAM	PF13871	Whole	1	78	LECA	1.73	1.67	1.80		Alpha.	SBNO1/2	55206 22904	SF3

PFAM	2*	Whole	1	267	ARCH divergence	2.79	2.43	3.14		Archaeal		-	
PFAM	2*	Whole	1	268	Duplication	2.57	2.22	2.95		Archaeal		-	
PFAM	2*	Whole	1	269	LECA	1.78	1.71	1.83		Archaeal	H3C13	653604	SF3
PFAM	2*	Whole	1	313	Duplication	2.41	2.08	2.81		Archaeal		-	
PFAM	2*	Whole	1	314	LECA	1.8	1.73	1.83		Archaeal	H2BC1/13	255626 8340	SF3
PFAM	2*	Whole	1	376	Duplication	2.1	1.83	2.49		Archaeal		-	
PFAM	2*	Whole	1	377	LECA	1.78	1.71	1.83		Archaeal	H2AZ1	3015	SF3
PFAM	2*	Whole	1	430	LECA	1.77	1.71	1.83		Archaeal	H2AX/C1/C19/C21	3014 221613 317772 723790	SF3
PFAM	PF00570	Whole	0	200	BACT divergence	2.78	2.43	3.09		Bacterial	-	-	
PFAM	PF00570	Whole	0	258	Duplication	2.18	1.93	2.55		Bacterial	-	-	
PFAM	PF00570	Whole	0	259	LECA	1.74	1.68	1.80		Bacterial	BLM	641	SF4
PFAM	PF00570	Whole	0	299	LECA	1.76	1.68	1.80		Bacterial	WRN	7486	SF4
PFAM	PF00183	Whole	1	283	BACT divergence	2.55	2.33	2.76		Bacterial	-	-	
PFAM	PF00183	Whole	1	284	Duplication	2.24	2.06	2.45		Bacterial	-	-	
PFAM	PF00183	Whole	1	395	LECA	1.79	1.74	1.81		Bacterial	HSP90AA1/B1	3320 3326	
PFAM	PF00183	Whole	1	285	Duplication	2.03	1.89	2.22		Bacterial	-	-	
PFAM	PF00183	Whole	1	266	partial LECA	1.79	1.74	1.81		Bacterial	-	-	

PFAM	PF00183	Whole	1	315	LECA	1.81	1.77	1.83		Bacterial	HSP90B1	7184	
PFAM	PF20470	Whole	2	115	nFECA divergence	2.92	2.92	2.92		Asgard.	-	-	
PFAM	PF20470	Whole	2	116	Duplication	1.99	1.83	2.22		Asgard.	-	-	
PFAM	PF20470	Whole	2	165	LECA	1.73	1.67	1.80		Asgard.	HELQ	113510	SF4
PFAM	PF20470	Whole	2	117	LECA	1.71	1.67	1.78		Asgard.	POLQ	10721	SF4
PFAM	PF01008	Whole	2	206	nFECA divergence	2.92	2.92	2.92		Asgard.	-	-	
PFAM	PF01008	Whole	2	207	Duplication	2.78	2.59	2.90		Asgard.	-	-	
PFAM	PF01008	Whole	2	312	LECA	1.74	1.68	1.80		Asgard.	EIF2B1	1967	SF6
PFAM	PF01008	Whole	2	208	Duplication	2.49	2.24	2.71		Asgard.	-	-	
PFAM	PF01008	Whole	2	209	LECA	1.75	1.68	1.80		Asgard.	EIF2B4	8890	SF6
PFAM	PF01008	Whole	2	243	LECA	1.75	1.68	1.80		Asgard.	EIF2B2	8892	SF6
PFAM	PF15985	Whole	2	174	ARCH divergence	2.84	2.51	3.13		Archaeal	-	-	
PFAM	PF15985	Whole	2	175	Duplication	2.44	2.14	2.78		Archaeal	-	-	
PFAM	PF15985	Whole	2	240	LECA	1.75	1.68	1.80		Archaeal	EXOSC2	23404	SF3
PFAM	PF15985	Whole	2	176	LECA	1.78	1.72	1.80		Archaeal	EXOSC3	51010	SF3
PFAM	PF17903	Whole	2	183	nFECA divergence	2.92	2.92	2.92		Asgard.	-	-	
PFAM	PF17903	Whole	2	184	Duplication	2.53	2.32	2.71		Asgard.	-	-	
PFAM	PF17903	Whole	2	185	LECA	1.76	1.69	1.80		Asgard.	PNO1	56902	
PFAM	PF17903	Whole	2	255	LECA	1.73	1.67	1.79		Asgard.	KRR1	11103	

PFAM	PF02735	Whole	2	218	mFECA divergence	2.24	2.24	2.24		Alpha.	-	-	
PFAM	PF02735	Whole	2	219	Duplication	2.00	1.90	2.10		Alpha.	-	-	
PFAM	PF02735	Whole	2	302	LECA	1.71	1.67	1.77		Alpha.	XRCC6	2547	SF4
PFAM	PF02735	Whole	2	220	LECA	1.72	1.67	1.79		Alpha.	XRCC5	7520	SF4
PFAM	PF02738	Cluster	2	122	BACT divergence	2.45	2.16	2.72		Bacterial	-	-	
PFAM	PF02738	Cluster	2	123	Duplication	2.08	1.89	2.36		Bacterial	-	-	
PFAM	PF02738	Cluster	2	124	LECA	1.73	1.67	1.80		Bacterial	-	-	
PFAM	PF02738	Cluster	2	141	LECA	1.71	1.67	1.79		Bacterial	XDH,AOX1	7498 316	
PFAM	PF05063	Whole	1	121	mFECA divergence	2.24	2.24	2.24		Alpha.	-	-	
PFAM	PF05063	Whole	1	122	Duplication	2.19	2.11	2.24		Alpha.	-	-	
PFAM	PF05063	Whole	1	210	LECA	1.74	1.67	1.80		Alpha.	METTL4	64863	SF3
PFAM	PF05063	Whole	1	123	Duplication	2.06	1.96	2.15		Alpha.	-	-	
PFAM	PF05063	Whole	1	196	LECA	1.74	1.68	1.80		Alpha.	-	-	
PFAM	PF05063	Whole	1	124	Duplication	1.96	1.85	2.07		Alpha.	-	-	
PFAM	PF05063	Whole	1	125	LECA	1.71	1.67	1.79		Alpha.	METTL14	57721	SF3
PFAM	PF05063	Whole	1	156	LECA	1.73	1.67	1.80		Alpha.	METTL3	56339	SF3
PFAM	PF05192	Whole	2	292	mFECA divergence	2.24	2.24	2.24		Alpha.	-	-	
PFAM	PF05192	Whole	2	293	Duplication	2.14	2.08	2.18		Alpha.	-	-	

PFAM	PF05192	Whole	2	429	Duplication	2.00	1.91	2.08		Alpha.	-	-	
PFAM	PF05192	Whole	2	430	LECA	1.70	1.67	1.77		Alpha.	MSH3	4437	SF4
PFAM	PF05192	Whole	2	468	LECA	1.75	1.68	1.80		Alpha.	MSH6	2956	SF4
PFAM	PF05192	Whole	2	294	Duplication	2.05	1.98	2.11		Alpha.	-	-	
PFAM	PF05192	Whole	2	401	LECA	1.73	1.67	1.80		Alpha.	MSH5	4439	SF4
PFAM	PF05192	Whole	2	295	Duplication	1.95	1.87	2.03		Alpha.	-	-	
PFAM	PF05192	Whole	2	369	LECA	1.71	1.67	1.78		Alpha.	MSH4	4438	SF4
PFAM	PF05192	Whole	2	296	LECA	1.77	1.71	1.80		Alpha.	MSH2	4436	SF4
PFAM	PTHR23091_SF268	Cluster	2	186	nFECA divergence	2.92	2.92	2.92		Asgard.	-	-	
PFAM	PTHR23091_SF268	Cluster	2	187	Duplication	2.43	2.08	2.75		Asgard.	-	-	
PFAM	PTHR23091_SF268	Cluster	2	262	LECA	1.73	1.67	1.80		Asgard.	NAA20	51126	SF6
PFAM	PTHR23091_SF268	Cluster	2	188	LECA	1.75	1.68	1.80		Asgard.	NAA10/11	84779<br>8260	SF6
PFAM	PF01798	Whole	2	298	nFECA divergence	2.92	2.92	2.92		Asgard.	-	-	
PFAM	PF01798	Whole	2	299	Duplication	2.53	2.32	2.71		Asgard.	-	-	
PFAM	PF01798	Whole	2	492	LECA	1.78	1.71	1.80		Asgard.	PRPF31	26121	SF3
PFAM	PF01798	Whole	2	300	Duplication	2.03	1.86	2.26		Asgard.	-	-	
PFAM	PF01798	Whole	2	398	LECA	1.78	1.73	1.80		Asgard.	NOP56	10528	SF3
PFAM	PF01798	Whole	2	301	LECA	1.72	1.67	1.79		Asgard.	NOP58	51602	SF3
Custom HMM	NP_001013182	Whole	2	198	nFECA divergence	2.92	2.92	2.92		Asgard.	-	-	

Custom HMM	NP_001013182	Whole	2	199	Duplication	2.56	2.29	2.77		Asgard.	-	-	
Custom HMM	NP_001013182	Whole	2	311	LECA	1.76	1.68	1.80		Asgard.	TAT	6898	
Custom HMM	NP_001013182	Whole	2	200	LECA	1.73	1.67	1.80		Asgard.	GPT/2	2875 84706	
Custom HMM	NP_001152796	Whole	1	156	BACT divergence	2.80	2.35	3.23		Bacterial	-	-	
Custom HMM	NP_001152796	Whole	1	157	Duplication	2.53	2.13	3.04		Bacterial	-	-	
Custom HMM	NP_001152796	Whole	1	158	partial LECA	1.71	1.67	1.79		Bacterial	GAA	2548	
Custom HMM	NP_001152796	Whole	1	220	LECA	1.69	1.67	1.75		Bacterial	GANAB/GANC	23193 2595	
Custom HMM	NP_598555	Whole	2	151	nFECA divergence	2.92	2.92	2.92		Asgard.	-	-	
Custom HMM	NP_598555	Whole	2	152	Duplication	2.80	2.55	2.91		Asgard.	-	-	
Custom HMM	NP_598555	Whole	2	153	LECA	1.70	1.67	1.75		Asgard.	EPRS1	2058	
Custom HMM	NP_598555	Whole	2	218	LECA	1.75	1.69	1.80		Asgard.	QARS1	5859	
PFAM	PF00483	Whole	2	147	ARCH divergence	2.60	2.32	2.89		Archaeal		-	
PFAM	PF00483	Whole	2	148	Duplication	2.31	2.06	2.60		Archaeal		-	
PFAM	PF00483	Whole	2	149	LECA	1.75	1.68	1.80		Archaeal	GMPPA	29926	4
PFAM	PF00483	Whole	2	195	LECA	1.76	1.68	1.80		Archaeal	GMPPB	29925	4
Custom HMM	O95786	Whole	2	215	nFECA divergence	2.92	2.92	2.92		Asgard.	-	-	

Custom HMM	O95786	Whole	2	216	Duplication	2.52	2.14	2.83		Asgard.	-	-	
Custom HMM	O95786	Whole	2	337	LECA	1.73	1.67	1.80		Asgard.	FANCM	57697	SF4
Custom HMM	O95786	Whole	2	217	Duplication	2.02	1.81	2.42		Asgard.	-	-	
Custom HMM	O95786	Whole	2	218	LECA	1.74	1.67	1.80		Asgard.	IFIH1,RIGI,DHX58	64135<br>23586<br>79132	4, SF4
Custom HMM	O95786	Whole	2	259	LECA	1.75	1.68	1.80		Asgard.	DICER1	23405	4, SF4
Custom HMM	P13196	Whole	2	168	mFECA divergence	2.24	2.24	2.24		Alpha.	-	-	
Custom HMM	P13196	Whole	2	169	Duplication	2.08	1.99	2.16		Alpha.	-	-	
Custom HMM	P13196	Whole	2	265	LECA	1.76	1.68	1.80		Alpha.	SPTLC1	10558	4
Custom HMM	P13196	Whole	2	170	LECA	1.72	1.67	1.79		Alpha.	SPTLC2/3	55304<br>9517	4
Custom HMM	P16278	Whole	2	86	BACT divergence	1.93	1.77	2.18		Bacterial	-	-	
Custom HMM	P16278	Whole	2	87	Duplication	1.87	1.74	2.10		Bacterial	-	-	
Custom HMM	P16278	Whole	2	135	partial LECA	1.10	0.77	1.67		Bacterial	GLB1L2/3	89944<br>112937	
Custom HMM	P16278	Whole	2	88	LECA	1.71	1.67	1.79		Bacterial	GLB1/1L	79411<br>2720	
Custom HMM	P18405	Whole	2	132	nFECA divergence	2.92	2.92	2.92		Asgard.	-	-	
Custom HMM	P18405	Whole	2	133	Duplication	2.61	2.25	2.86		Asgard.	-	-	
Custom HMM	P18405	Whole	2	209	LECA	1.73	1.67	1.80		Asgard.	SRD5A1	6715	4

Custom HMM	P18405	Whole	2	134	Duplication	2.18	1.90	2.54		Asgard.	-	-	
Custom HMM	P18405	Whole	2	180	LECA	1.71	1.67	1.79		Asgard.	TECRL	253017	4
Custom HMM	P18405	Whole	2	135	LECA	1.76	1.68	1.80		Asgard.	SRD5A3	79644	4
Custom HMM	P27708	Whole	1	123	BACT divergence	2.25	2.03	2.57		Bacterial	-	-	
Custom HMM	P27708	Whole	1	124	Duplication	1.93	1.83	2.07		Bacterial	-	-	
Custom HMM	P27708	Whole	1	125	LECA	1.75	1.68	1.80		Bacterial	CPS1	1373	4
Custom HMM	P27708	Whole	1	163	LECA	1.77	1.71	1.80		Bacterial	CAD	790	4
Custom HMM	P30711	Whole	2	105	mFECA divergence	2.24	2.24	2.24		Alpha.	-	-	
Custom HMM	P30711	Whole	2	106	Duplication	2.00	1.85	2.15		Alpha.	-	-	
Custom HMM	P30711	Whole	2	165	LECA	1.74	1.68	1.80		Alpha.	EEF1G	1937	
Custom HMM	P30711	Whole	2	107	LECA	1.70	1.67	1.77		Alpha.	GSTT2/4	653689 25774	
Custom HMM	P35523	Whole	1	242	BACT divergence	2.86	2.56	3.09		Bacterial	-	-	
Custom HMM	P35523	Whole	1	243	Duplication	2.36	2.05	2.69		Bacterial	-	-	
Custom HMM	P35523	Whole	1	359	LECA	1.77	1.70	1.80		Bacterial	CLCN6/7	1185 1186	4
Custom HMM	P35523	Whole	1	244	Duplication	2.16	1.90	2.48		Bacterial	-	-	

Custom HMM	P35523	Whole	1	245	LECA	1.75	1.68	1.80		Bacterial	CLCN1/2,CLCNKA/B	1181 1180 1188 1187	4
Custom HMM	P35523	Whole	1	299	LECA	1.75	1.68	1.80		Bacterial	CLCN3/4/5	1182 1184 1183	4
Custom HMM	P46977	Whole	1	139	ARCH divergence	2.89	2.60	3.12		Archaeal	-	-	
Custom HMM	P46977	Whole	1	140	Duplication	2.48	2.18	2.80		Archaeal	-	-	
Custom HMM	P46977	Whole	1	244	partial LECA	1.75	1.68	1.80		Archaeal	-	-	
Custom HMM	P46977	Whole	1	141	Duplication	2.03	1.82	2.33		Archaeal	-	-	
Custom HMM	P46977	Whole	1	213	partial LECA	1.66	1.35	1.99		Archaeal	STT3B	201595	4
Custom HMM	P46977	Whole	1	142	LECA	1.76	1.69	1.80		Archaeal	STT3A	3703	4
Custom HMM	P53582	Whole	2	228	nFECA divergence	2.92	2.92	2.92		Asgard.	-	-	
Custom HMM	P53582	Whole	2	229	Duplication	2.61	2.37	2.80		Asgard.	-	-	
Custom HMM	P53582	Whole	2	326	LECA	1.77	1.71	1.80		Asgard.	PA2G4	5036	SF6
Custom HMM	P53582	Whole	2	230	LECA	1.75	1.68	1.80		Asgard.	METAP2	10988	SF6
PFAM	PF01569	Whole	2	119	nFECA divergence	2.56	2.21	3.02		Asgard.	-	-	
PFAM	PF01569	Whole	2	120	Duplication	2.26	1.98	2.67		Asgard.	-	-	
PFAM	PF01569	Whole	2	170	LECA	1.75	1.68	1.80		Asgard.	DOLPP1	57171	4

PFAM	PF01569	Whole	2	121	LECA	1.73	1.67	1.80		Asgard.	SGPP1/2	130367 81537	4
PFAM	PF01433	Path	2	128	BACT divergence	2.19	1.97	2.47		Bacterial	-	-	
PFAM	PF01433	Path	2	129	Duplication	1.96	1.82	2.18		Bacterial	-	-	
PFAM	PF01433	Path	2	130	LECA	1.71	1.67	1.78		Bacterial	RNPEP	6051	
PFAM	PF01433	Path	2	155	LECA	1.76	1.68	1.80		Bacterial	LTA4H	4048	
PFAM	PF00675	Path	2	244	mFECA divergence	2.24	2.24	2.24		Alpha.	-	-	
PFAM	PF00675	Path	2	245	Duplication	2.06	1.94	2.16		Alpha.	-	-	
PFAM	PF00675	Path	2	329	LECA	1.73	1.67	1.80		Alpha.	PMPCA, UQCRC2	23203 7385	4
PFAM	PF00675	Path	2	246	LECA	1.72	1.67	1.79		Alpha.	PMPCB, UQCRC!	7384 9512	4
PFAM	PF00557	Whole	2	144	mFECA divergence	2.24	2.24	2.24		Alpha.	-	-	
PFAM	PF00557	Whole	2	145	Duplication	1.88	1.74	2.08		Alpha.	-	-	
PFAM	PF00557	Whole	2	223	partial LECA	1.32	1.02	1.70		Alpha.	-	-	
PFAM	PF00557	Whole	2	146	LECA	1.72	1.67	1.80		Alpha.	XPNPEP1/2	7511 7512	
PFAM	PF00025	Cluster	1	477	nFECA divergence	2.92	2.92	2.92		Asgard.	-	-	
PFAM	PF00025	Cluster	1	478	Duplication	2.82	2.59	2.91		Asgard.	-	-	
PFAM	PF00025	Cluster	1	802	Duplication	1.70	1.36	2.09		Asgard.	-	-	
PFAM	PF00025	Cluster	1	863	LECA	1.32	1.26	1.35		Asgard.	ARL3	403	

PFAM	PF00025	Cluster	1	803	LECA	1.32	1.27	1.35		Asgard.	ARL2	402	
PFAM	PF00025	Cluster	1	479	Duplication	2.71	2.42	2.89		Asgard.	-	-	
PFAM	PF00025	Cluster	1	656	Duplication	2.52	2.12	2.82		Asgard.	-	-	
PFAM	PF00025	Cluster	1	657	LECA	1.76	1.68	1.80		Asgard.	ARL6	84100	
PFAM	PF00025	Cluster	1	689	Duplication	2.26	1.87	2.68		Asgard.	-	-	
PFAM	PF00025	Cluster	1	751	LECA	1.75	1.68	1.80		Asgard.	ARL5A/B/C	26225 390790 221079	
PFAM	PF00025	Cluster	1	690	LECA	1.76	1.68	1.80		Asgard.	ARL1	400	
PFAM	PF00025	Cluster	1	480	Duplication	2.48	2.09	2.80		Asgard.	-	-	
PFAM	PF00025	Cluster	1	481	partial LECA	1.74	1.68	1.80		Asgard.	-	-	
PFAM	PF00025	Cluster	1	493	Duplication	2.32	1.95	2.70		Asgard.	-	-	
PFAM	PF00025	Cluster	1	615	partial LECA	0.67	0.62	0.75		Asgard.	ARL4A/C/D,ARL11/14	80117 115761 10123 379 10124	
PFAM	PF00025	Cluster	1	494	Duplication	2.15	1.84	2.55		Asgard.	-	-	
PFAM	PF00025	Cluster	1	584	LECA	1.75	1.68	1.80		Asgard.	ARF6	382	
PFAM	PF00025	Cluster	1	495	Duplication	1.91	1.73	2.26		Asgard.	-	-	
PFAM	PF00025	Cluster	1	555	partial LECA	1.37	1.11	1.56		Asgard.	ARF3	377	
PFAM	PF00025	Cluster	1	496	LECA	1.76	1.68	1.80		Asgard.	ARF4/5	381 378	
PFAM	PF00091	Whole	2	410	nFECA divergence	2.92	2.92	2.92		Asgard.		-	

PFAM	PF00091	Whole	2	411	Duplication	2.67	2.41	2.84		Asgard.		-	
PFAM	PF00091	Whole	2	412	LECA	1.77	1.70	1.80		Asgard.	TUBD1	51174	2
PFAM	PF00091	Whole	2	445	Duplication	2.52	2.25	2.75		Asgard.		-	
PFAM	PF00091	Whole	2	770	LECA	1.75	1.68	1.80		Asgard.	TUBE1	51175	2
PFAM	PF00091	Whole	2	446	Duplication	2.35	2.07	2.61		Asgard.		-	
PFAM	PF00091	Whole	2	632	LECA	1.76	1.68	1.80		Asgard.	TUBA/1A8/L3	79861<br>51807<br>Q71U36	2
PFAM	PF00091	Whole	2	447	Duplication	2.18	1.92	2.47		Asgard.		-	
PFAM	PF00091	Whole	2	448	LECA	1.77	1.70	1.80		Asgard.	TUBG1	7283	2
PFAM	PF00091	Whole	2	522	LECA	1.76	1.68	1.80		Asgard.	TUBB2B/3/6	347733<br>10381<br>84617	2
PFAM	PF00432	Whole	2	198	nFECA divergence	2.92	2.92	2.92		Asgard.	-	-	
PFAM	PF00432	Whole	2	199	Duplication	2.62	2.36	2.82		Asgard.	-	-	
PFAM	PF00432	Whole	2	307	LECA	1.72	1.67	1.79		Asgard.	RABGGTB	5876	4
PFAM	PF00432	Whole	2	200	Duplication	2.40	2.12	2.67		Asgard.	-	-	
PFAM	PF00432	Whole	2	201	LECA	1.75	1.68	1.80		Asgard.	PGGT1B	5229	4
PFAM	PF00432	Whole	2	251	LECA	1.77	1.69	1.80		Asgard.	FNTB	2342	4
PFAM	PF00687	Whole	2	241	nFECA divergence	2.92	2.92	2.92		Asgard.		-	
PFAM	PF00687	Whole	2	242	Duplication	2.51	2.09	2.83		Asgard.		-	
PFAM	PF00687	Whole	2	338	LECA	1.76	1.69	1.80		Asgard.	RSL1D1	26156	SF6
PFAM	PF00687	Whole	2	243	LECA	1.77	1.70	1.80		Asgard.	RPL10A	4736	SF6

PFAM	PF00850	Whole	2	172	nFECA divergence	2.92	2.92	2.92		Asgard.		-	
PFAM	PF00850	Whole	2	173	Duplication	1.96	1.81	2.19		Asgard.		-	
PFAM	PF00850	Whole	2	174	LECA	1.75	1.68	1.80		Asgard.	HDAC1/2	-	SF3
PFAM	PF00850	Whole	2	248	LECA	1.76	1.69	1.80		Asgard.	HDAC3	-	SF3
PFAM	PF00899	Whole	2	476	nFECA divergence	2.92	2.92	2.92		Asgard.		-	
PFAM	PF00899	Whole	2	477	Duplication	2.76	2.64	2.85		Asgard.		-	
PFAM	PF00899	Whole	2	478	LECA	1.73	1.67	1.80		Asgard.	UBA5	79876	SF5
PFAM	PF00899	Whole	2	542	Duplication	2.62	2.48	2.74		Asgard.		-	
PFAM	PF00899	Whole	2	543	LECA	1.76	1.68	1.80		Asgard.	UBA3	9039	SF5
PFAM	PF00899	Whole	2	619	Duplication	2.53	2.39	2.66		Asgard.		-	
PFAM	PF00899	Whole	2	620	LECA	1.78	1.73	1.80		Asgard.	UBA2	10054	SF5
PFAM	PF00899	Whole	2	689	Duplication	2.27	2.12	2.42		Asgard.		-	
PFAM	PF00899	Whole	2	690	Duplication	2.08	1.93	2.25		Asgard.		-	
PFAM	PF00899	Whole	2	691	LECA	1.77	1.69	1.80		Asgard.	SUMO1	10055	SF5
PFAM	PF00899	Whole	2	735	LECA	1.71	1.67	1.78		Asgard.	NAE1	8883	SF5
PFAM	PF00899	Whole	2	785	Duplication	1.87	1.79	1.98		Asgard.		-	
PFAM	PF00899	Whole	2	786	LECA	1.78	1.73	1.81		Asgard.	UBA6	55236	SF5
PFAM	PF00899	Whole	2	820	Duplication	1.82	1.75	1.91		Asgard.		-	
PFAM	PF00899	Whole	2	821	LECA	1.74	1.68	1.80		Asgard.		-	
PFAM	PF00899	Whole	2	856	LECA	1.70	1.67	1.77		Asgard.	UBA1/7	7317 7318	SF5

PFAM	PF01119	Whole	2	268	mFECA divergence	2.24	2.24	2.24		Alpha.	-	-	
PFAM	PF01119	Whole	2	269	Duplication	2.05	1.96	2.14		Alpha.	-	-	
PFAM	PF01119	Whole	2	369	LECA	1.75	1.68	1.80		Alpha.	MLH1	4292	SF4
PFAM	PF01119	Whole	2	270	Duplication	1.89	1.79	2.00		Alpha.	-	-	
PFAM	PF01119	Whole	2	271	partial LECA	1.51	1.17	1.78		Alpha.	PMS1	5378	SF4
PFAM	PF01119	Whole	2	295	LECA	1.69	1.67	1.75		Alpha.	PMS2	5395	SF4
PFAM	PF01217	Whole	2	455	nFECA divergence	2.92	2.92	2.92		Asgard.		-	
PFAM	PF01217	Whole	2	456	Duplication	2.87	2.76	2.91		Asgard.		-	
PFAM	PF01217	Whole	2	687	Duplication	2.68	2.39	2.87		Asgard.		-	
PFAM	PF01217	Whole	2	688	LECA	1.77	1.70	1.80		Asgard.	COPZ1/2	51226 22818	3
PFAM	PF01217	Whole	2	729	Duplication	2.40	2.11	2.68		Asgard.		-	
PFAM	PF01217	Whole	2	730	LECA	1.76	1.68	1.80		Asgard.	AP3S1/2	1176 10239	3
PFAM	PF01217	Whole	2	776	Duplication	2.26	1.97	2.57		Asgard.		-	
PFAM	PF01217	Whole	2	777	LECA	1.75	1.68	1.80		Asgard.	AP4S1	11154	3
PFAM	PF01217	Whole	2	806	Duplication	1.98	1.78	2.29		Asgard.		-	
PFAM	PF01217	Whole	2	807	LECA	1.75	1.68	1.80		Asgard.	AP2S1	1175	3
PFAM	PF01217	Whole	2	847	LECA	1.76	1.68	1.80		Asgard.	AP1S1/2/3	8905 1174 130340	3
PFAM	PF01217	Whole	2	457	Duplication	2.80	2.61	2.90		Asgard.		-	

PFAM	PF01217	Whole	2	458	LECA	1.78	1.72	1.80		Asgard.	ARCN1 (COPD)	372	3
PFAM	PF01217	Whole	2	506	Duplication	2.58	2.31	2.80		Asgard.		-	
PFAM	PF01217	Whole	2	507	LECA	1.76	1.68	1.80		Asgard.	AP3M1/2	10947<br>26985	3
PFAM	PF01217	Whole	2	551	Duplication	2.39	2.13	2.66		Asgard.		-	
PFAM	PF01217	Whole	2	552	LECA	1.77	1.70	1.80		Asgard.	AP4M1	9179	3
PFAM	PF01217	Whole	2	586	Duplication	2.22	1.97	2.52		Asgard.		-	
PFAM	PF01217	Whole	2	587	LECA	1.75	1.68	1.80		Asgard.	AP1M1/2	8907<br>10053	3
PFAM	PF01217	Whole	2	637	LECA	1.76	1.68	1.80		Asgard.	AP2M1	1173	3
PFAM	PF01398	Subtree	2	176	nFECA divergence	2.92	2.92	2.92		Asgard.		-	
PFAM	PF01398	Subtree	2	177	Duplication	2.46	2.12	2.75		Asgard.		-	
PFAM	PF01398	Subtree	2	265	LECA	1.77	1.71	1.80		Asgard.	COPS5	10987	SF5
PFAM	PF01398	Subtree	2	178	LECA	1.76	1.69	1.80		Asgard.	PSMD14	10213	SF5
PFAM	PF01398	Subtree	2	344	nFECA divergence	2.92	2.92	2.92		Asgard.		-	
PFAM	PF01398	Subtree	2	345	Duplication	2.80	2.56	2.91		Asgard.		-	
PFAM	PF01398	Subtree	2	609	LECA	1.75	1.68	1.80		Asgard.	EIF3H	8667	SF5
PFAM	PF01398	Subtree	2	346	Duplication	2.68	2.39	2.87		Asgard.		-	
PFAM	PF01398	Subtree	2	547	LECA	1.73	1.67	1.80		Asgard.	PRPF8	10594	SF5
PFAM	PF01398	Subtree	2	347	Duplication	2.51	2.20	2.78		Asgard.		-	
PFAM	PF01398	Subtree	2	469	LECA	1.76	1.69	1.80		Asgard.	PSMD7	5713	SF5

PFAM	PF01398	Subtree	2	348	Duplication	2.35	2.03	2.67		Asgard.		-	
PFAM	PF01398	Subtree	2	349	LECA	1.77	1.69	1.80		Asgard.	COPS6	10980	SF5, SF6
PFAM	PF01398	Subtree	2	407	LECA	1.76	1.69	1.80		Asgard.	EIF3F	8665	SF5, SF6
PFAM	PF01399	Whole	2	477	nFECA divergence	2.92	2.92	2.92		Asgard.		-	
PFAM	PF01399	Whole	2	478	Duplication	2.89	2.83	2.92		Asgard.		-	
PFAM	PF01399	Whole	2	844	Duplication	2.54	2.23	2.80		Asgard.		-	
PFAM	PF01399	Whole	2	902	LECA	1.75	1.68	1.80		Asgard.	PSMD13	5719	SF5
PFAM	PF01399	Whole	2	845	Duplication	2.30	1.95	2.65		Asgard.		-	
PFAM	PF01399	Whole	2	878	LECA	1.75	1.68	1.80		Asgard.	COPS7A/B	64708<br>50813	SF5
PFAM	PF01399	Whole	2	846	LECA	1.76	1.69	1.80		Asgard.	EIF3M	10480	SF5
PFAM	PF01399	Whole	2	479	Duplication	2.85	2.75	2.91		Asgard.		-	
PFAM	PF01399	Whole	2	715	Duplication	2.74	2.52	2.88		Asgard.		-	
PFAM	PF01399	Whole	2	802	LECA	1.74	1.68	1.80		Asgard.	PSMD12	5718	SF5
PFAM	PF01399	Whole	2	716	Duplication	2.53	2.23	2.78		Asgard.		-	
PFAM	PF01399	Whole	2	763	LECA	1.77	1.69	1.80		Asgard.	PCID2	55795	SF4, SF5
PFAM	PF01399	Whole	2	717	LECA	1.75	1.68	1.80		Asgard.	PSMD3	5709	SF5
PFAM	PF01399	Whole	2	480	Duplication	2.74	2.57	2.86		Asgard.		-	
PFAM	PF01399	Whole	2	620	Duplication	2.34	2.08	2.60		Asgard.		-	
PFAM	PF01399	Whole	2	681	LECA	1.75	1.68	1.80		Asgard.	GPS1 (COPS1)	2873	SF5
PFAM	PF01399	Whole	2	621	LECA	1.75	1.68	1.80		Asgard.	PSMD6	9861	SF5

PFAM	PF01399	Whole	2	481	Duplication	2.61	2.39	2.80		Asgard.		-	
PFAM	PF01399	Whole	2	566	LECA	1.74	1.68	1.80		Asgard.	COPS2	9318	SF5
PFAM	PF01399	Whole	2	482	Duplication	2.45	2.18	2.70		Asgard.		-	
PFAM	PF01399	Whole	2	540	LECA	1.74	1.68	1.80		Asgard.	COPS3	8533	SF5
PFAM	PF01399	Whole	2	483	LECA	1.75	1.68	1.80		Asgard.	PSMD11	5717	SF5
PFAM	PF01423	Subtree	2	379	nFECA divergence	2.92	2.92	2.92		Asgard.		-	
PFAM	PF01423	Subtree	2	380	Duplication	2.86	2.73	2.91		Asgard.		-	
PFAM	PF01423	Subtree	2	658	Duplication	2.53	2.01	2.87		Asgard.		-	
PFAM	PF01423	Subtree	2	659	LECA	1.76	1.68	1.80		Asgard.	SNRPD2	6633	2, SF3
PFAM	PF01423	Subtree	2	691	LECA	1.76	1.68	1.80		Asgard.	LSM3	27258	2, SF3
PFAM	PF01423	Subtree	2	381	Duplication	2.79	2.60	2.90		Asgard.		-	
PFAM	PF01423	Subtree	2	586	Duplication	2.48	1.97	2.83		Asgard.		-	
PFAM	PF01423	Subtree	2	625	LECA	1.75	1.68	1.80		Asgard.	SNRPE	6635	2, SF3
PFAM	PF01423	Subtree	2	587	LECA	1.75	1.68	1.80		Asgard.	LSM5	23658	2, SF3
PFAM	PF01423	Subtree	2	382	Duplication	2.71	2.46	2.87		Asgard.		-	
PFAM	PF01423	Subtree	2	536	Duplication	2.49	2.05	2.79		Asgard.		-	
PFAM	PF01423	Subtree	2	571	partial LECA	2.20	1.68	2.63		Asgard.	NAA38	84316	2, SF3
PFAM	PF01423	Subtree	2	537	LECA	1.75	1.68	1.80		Asgard.	SNRPB	6628	2, SF3
PFAM	PF01423	Subtree	2	383	Duplication	2.57	2.24	2.81		Asgard.		-	
PFAM	PF01423	Subtree	2	465	Duplication	2.34	1.92	2.70		Asgard.		-	

PFAM	PF01423	Subtree	2	504	partial LECA	1.76	1.68	1.80		Asgard.	LSM8	51691	2, SF3
PFAM	PF01423	Subtree	2	466	LECA	1.76	1.68	1.80		Asgard.	LSM1	27257	2, SF3
PFAM	PF01423	Subtree	2	384	Duplication	2.28	1.87	2.67		Asgard.		-	
PFAM	PF01423	Subtree	2	385	LECA	1.75	1.68	1.80		Asgard.	SNRPG/SNRPGP15	6637<br>100130932	2, SF3
PFAM	PF01423	Subtree	2	419	LECA	1.74	1.68	1.80		Asgard.	LSM7	51690	2, SF3
PFAM	PF01423	Subtree	2	282	nFECA divergence	2.92	2.92	2.92		Asgard.		-	
PFAM	PF01423	Subtree	2	283	Duplication	2.82	2.61	2.91		Asgard.		-	
PFAM	PF01423	Subtree	2	462	Duplication	2.47	1.98	2.84		Asgard.		-	
PFAM	PF01423	Subtree	2	496	LECA	1.75	1.68	1.80		Asgard.	LSM6	11157	2, SF3
PFAM	PF01423	Subtree	2	463	LECA	1.75	1.68	1.80		Asgard.	SNRPF	6636	2, SF3
PFAM	PF01423	Subtree	2	284	Duplication	2.64	2.33	2.86		Asgard.		-	
PFAM	PF01423	Subtree	2	385	Duplication	2.26	1.85	2.70		Asgard.		-	
PFAM	PF01423	Subtree	2	386	LECA	1.74	1.68	1.80		Asgard.	LSM4	25804	2, SF3
PFAM	PF01423	Subtree	2	415	LECA	1.75	1.68	1.80		Asgard.	SNRPD3	6634	2, SF3
PFAM	PF01423	Subtree	2	285	Duplication	2.42	2.06	2.74		Asgard.		-	
PFAM	PF01423	Subtree	2	349	LECA	1.75	1.68	1.80		Asgard.	SNRPD1	6632	2, SF3
PFAM	PF01423	Subtree	2	286	Duplication	2.19	1.87	2.56		Asgard.		-	
PFAM	PF01423	Subtree	2	287	LECA	1.76	1.68	1.80		Asgard.	LSM10	84967	2, SF3
PFAM	PF01423	Subtree	2	307	LECA	1.75	1.68	1.80		Asgard.	LSM2	57819	2, SF3
PFAM	PF01553	Whole	1	175	BACT divergence	2.65	2.41	2.86		Bacterial		-	

PFAM	PF01553	Whole	1	176	Duplication	2.29	2.06	2.54		Bacterial		-	
PFAM	PF01553	Whole	1	177	LECA	1.74	1.67	1.80		Bacterial	GPAT3/4	84803<br>137964	4
PFAM	PF01553	Whole	1	241	LECA	1.72	1.67	1.79		Bacterial	LPCAT1/2/4	79888<br>254531<br>54947	4
PFAM	PF01751	Path	2	145	nFECA divergence	2.92	2.92	2.92		Asgard.	-	-	
PFAM	PF01751	Path	2	146	Duplication	2.42	2.18	2.65		Asgard.	-	-	
PFAM	PF01751	Path	2	147	LECA	1.72	1.67	1.79		Asgard.	TOP3B	8940	4
PFAM	PF01751	Path	2	199	LECA	1.74	1.68	1.80		Asgard.	TOP3A	7156	4
PFAM	PF02359	Whole	2	155	nFECA divergence	2.92	2.92	2.92		Asgard.		-	
PFAM	PF02359	Whole	2	156	Duplication	2.51	2.20	2.76		Asgard.		-	
PFAM	PF02359	Whole	2	247	LECA	1.76	1.69	1.80		Asgard.	VCP	7415	3
PFAM	PF02359	Whole	2	157	LECA	1.77	1.70	1.80		Asgard.	NSF	4905	3
PFAM	PF04042	Whole	2	237	nFECA divergence	2.92	2.92	2.92		Asgard.	-	-	
PFAM	PF04042	Whole	2	238	Duplication	2.56	2.36	2.73		Asgard.	-	-	
PFAM	PF04042	Whole	2	352	LECA	1.77	1.70	1.80		Asgard.	POLD2	5425	SF2
PFAM	PF04042	Whole	2	239	Duplication	2.28	2.08	2.50		Asgard.	-	-	
PFAM	PF04042	Whole	2	304	LECA	1.74	1.68	1.80		Asgard.	POLA2	23649	SF2
PFAM	PF04042	Whole	2	240	LECA	1.75	1.68	1.80		Asgard.	POLE2	5427	SF2
PFAM	PF06733	Whole	2	218	nFECA divergence	2.92	2.92	2.92		Asgard.		-	

PFAM	PF06733	Whole	2	219	Duplication	2.57	2.39	2.72		Asgard.		-	
PFAM	PF06733	Whole	2	329	LECA	1.78	1.71	1.80		Asgard.	ERCC2	2068	SF2, SF4
PFAM	PF06733	Whole	2	220	Duplication	2.33	2.15	2.51		Asgard.		-	
PFAM	PF06733	Whole	2	221	LECA	1.74	1.67	1.80		Asgard.	DDX11/DDX11L8/DDX12P	1663<br>440081<br>100302090	SF4
PFAM	PF06733	Whole	2	268	Duplication	2.11	1.95	2.30		Asgard.		-	
PFAM	PF06733	Whole	2	269	LECA	1.74	1.68	1.80		Asgard.	BRIP1	83990	SF4
PFAM	PF06733	Whole	2	290	LECA	1.75	1.68	1.80		Asgard.	RTEL1	51750	SF4
PFAM	PF07297	Whole	2	72	nFECA divergence	2.92	2.92	2.92		Asgard.		-	
PFAM	PF07297	Whole	2	73	Duplication	2.51	2.06	2.88		Asgard.		-	
PFAM	PF07297	Whole	2	74	LECA	1.74	1.67	1.80		Asgard.	DPM2	8818	4
PFAM	PF07297	Whole	2	104	LECA	1.74	1.68	1.80		Asgard.	PIGP	51227	4
PFAM	PF13481	Subtree	2	142	nFECA divergence	2.92	2.92	2.92		Asgard.	-	-	
PFAM	PF13481	Subtree	2	143	Duplication	2.25	1.98	2.55		Asgard.	-	-	
PFAM	PF13481	Subtree	2	144	LECA	1.74	1.68	1.80		Asgard.	DMC1	11144	SF4
PFAM	PF13481	Subtree	2	173	LECA	1.75	1.68	1.80		Asgard.	RAD51	5888	SF4
PFAM	PF13481	Subtree	2	103	nFECA divergence	2.92	2.92	2.92		Asgard.	-	-	
PFAM	PF13481	Subtree	2	104	Duplication	2.26	2.03	2.55		Asgard.	-	-	
PFAM	PF13481	Subtree	2	160	LECA	1.75	1.68	1.80		Asgard.	RAD51C	5889	SF4

PFAM	PF13481	Subtree	2	105	Duplication	2.10	1.91	2.38		Asgard.	-	-	
PFAM	PF13481	Subtree	2	106	LECA	1.73	1.67	1.80		Asgard.	RAD51D	5892	SF4
PFAM	PF13481	Subtree	2	126	LECA	1.77	1.70	1.80		Asgard.	XRCC3	7517	SF4
PFAM	PF14464	Whole	2	229	nFECA divergence	2.92	2.92	2.92		Asgard.	-	-	
PFAM	PF14464	Whole	2	230	Duplication	2.55	2.22	2.81		Asgard.	-	-	
PFAM	PF14464	Whole	2	376	LECA	1.75	1.68	1.80		Asgard.	COPS5	10987	SF5
PFAM	PF14464	Whole	2	231	Duplication	2.35	2.00	2.69		Asgard.	-	-	
PFAM	PF14464	Whole	2	232	LECA	1.77	1.71	1.80		Asgard.	BRCC3	79184	SF5
PFAM	PF14464	Whole	2	278	LECA	1.76	1.68	1.80		Asgard.	PSMD14	10213	SF5
PFAM	PF10584	Whole	2	503	nFECA divergence	2.92	2.92	2.92		Asgard.	-	-	
PFAM	PF10584	Whole	2	504	Duplication	2.86	2.71	2.91		Asgard.	-	-	
PFAM	PF10584	Whole	2	893	LECA	1.75	1.68	1.80		Asgard.	PSMA5	5686	SF5
PFAM	PF10584	Whole	2	505	Duplication	2.78	2.58	2.90		Asgard.	-	-	
PFAM	PF10584	Whole	2	827	LECA	1.76	1.69	1.80		Asgard.	PSMA1	5682	SF5
PFAM	PF10584	Whole	2	506	Duplication	2.70	2.47	2.86		Asgard.	-	-	
PFAM	PF10584	Whole	2	701	Duplication	2.49	2.15	2.77		Asgard.	-	-	
PFAM	PF10584	Whole	2	767	LECA	1.77	1.69	1.80		Asgard.	PSMA4	5685	SF5
PFAM	PF10584	Whole	2	702	LECA	1.76	1.68	1.80		Asgard.	PSMA7/8	143471<br>5688	SF5
PFAM	PF10584	Whole	2	507	Duplication	2.58	2.29	2.80		Asgard.	-	-	
PFAM	PF10584	Whole	2	639	LECA	1.77	1.69	1.80		Asgard.	PSMA6	5687	SF5

PFAM	PF10584	Whole	2	508	Duplication	2.39	2.05	2.70		Asgard.	-	-	
PFAM	PF10584	Whole	2	509	LECA	1.76	1.68	1.80		Asgard.	PSMA2	5683	SF5
PFAM	PF10584	Whole	2	571	LECA	1.77	1.70	1.80		Asgard.	PSMA3	5684	SF5
PFAM	PF00227	Whole	1	487	nFECA divergence	2.92	2.92	2.92		Asgard.	-	-	
PFAM	PF00227	Whole	1	488	Duplication	2.87	2.74	2.91		Asgard.	-	-	
PFAM	PF00227	Whole	1	489	Duplication	2.65	2.31	2.87		Asgard.	-	-	
PFAM	PF00227	Whole	1	614	LECA	1.76	1.68	1.80		Asgard.	PSMB5/8/11	5693 122706 5696	SF5
PFAM	PF00227	Whole	1	490	Duplication	2.33	1.97	2.71		Asgard.	-	-	
PFAM	PF00227	Whole	1	491	LECA	1.77	1.70	1.80		Asgard.	PSMB6	5694	SF5
PFAM	PF00227	Whole	1	545	LECA	1.76	1.69	1.80		Asgard.	PSMB7/10	5695 5699	SF5
PFAM	PF00227	Whole	1	670	Duplication	2.75	2.50	2.89		Asgard.	-	-	
PFAM	PF00227	Whole	1	671	Duplication	2.48	2.10	2.79		Asgard.	-	-	
PFAM	PF00227	Whole	1	672	LECA	1.77	1.70	1.80		Asgard.	PSMB4	5692	SF5
PFAM	PF00227	Whole	1	723	LECA	1.78	1.73	1.81		Asgard.	PSMB2	5690	SF5
PFAM	PF00227	Whole	1	793	Duplication	2.47	2.11	2.78		Asgard.	-	-	
PFAM	PF00227	Whole	1	863	LECA	1.76	1.68	1.80		Asgard.	PSMB1	5689	SF5
PFAM	PF00227	Whole	1	794	LECA	1.77	1.70	1.80		Asgard.	PSMB3	5691	SF5
Panther DB	PTHR11129	Whole	2	161	nFECA divergence	2.92	2.92	2.92		Asgard.		-	
Panther DB	PTHR11129	Whole	2	162	Duplication	2.43	2.21	2.64		Asgard.		-	

Panther DB	PTHR11129	Whole	2	266	LECA	1.73	1.67	1.80		Asgard.	RABGGTA	5875	4
Panther DB	PTHR11129	Whole	2	163	Duplication	2.30	2.08	2.54		Asgard.		-	
Panther DB	PTHR11129	Whole	2	233	LECA	1.74	1.68	1.80		Asgard.	PTAR1	375743	4
Panther DB	PTHR11129	Whole	2	164	LECA	1.72	1.67	1.79		Asgard.	FNTA	2339	4
Panther DB	PTHR11937	Whole	2	366	nFECA divergence	2.92	2.92	2.92		Asgard.		-	
Panther DB	PTHR11937	Whole	2	367	Duplication	2.74	2.47	2.90		Asgard.		-	
Panther DB	PTHR11937	Whole	2	642	LECA	1.76	1.69	1.80		Asgard.	ACTR3/B	10096 57180	2
Panther DB	PTHR11937	Whole	2	368	Duplication	2.59	2.30	2.82		Asgard.		-	
Panther DB	PTHR11937	Whole	2	369	Duplication	2.41	2.06	2.72		Asgard.		-	
Panther DB	PTHR11937	Whole	2	420	LECA	1.77	1.69	1.80		Asgard.	ACTL6A/B	86 51412	2
Panther DB	PTHR11937	Whole	2	370	LECA	1.76	1.69	1.80		Asgard.	ACTR1A/B	10120 10121	2
Panther DB	PTHR11937	Whole	2	467	Duplication	2.32	1.98	2.67		Asgard.		-	
Panther DB	PTHR11937	Whole	2	576	LECA	1.77	1.70	1.80		Asgard.	ACTR2	10097	2
Panther DB	PTHR11937	Whole	2	468	LECA	1.76	1.69	1.80		Asgard.	Actins (ACTBL2/C1/G1, POTEKP	70 71 440915 345651	2
Panther DB	PTHR19375_SF144	Subtree	2	190	mFECA divergence	2.6	2.4	2.77		Bacterial		-	
Panther DB	PTHR19375_SF144	Subtree	2	191	Duplication	2.26	2.1	2.43		Bacterial		-	

Panther DB	PTHR19375_SF144	Subtree	2	192	LECA	1.79	1.72	1.83		Bacterial	HSPA14	51182	
Panther DB	PTHR19375_SF144	Subtree	2	221	Duplication	2.08	1.95	2.24		Bacterial		-	
Panther DB	PTHR19375_SF144	Subtree	2	222	LECA	1.76	1.71	1.82		Bacterial	HYOU1	10525	
Panther DB	PTHR19375_SF144	Subtree	2	281	LECA	1.73	1.7	1.78		Bacterial	HSPH1,HSPA4/L	10808	
Panther DB	PTHR23073	Path	2	490	nFECA divergence	2.92	2.92	2.92		Asgard.	-	-	
Panther DB	PTHR23073	Path	2	491	Duplication	2.78	2.61	2.90		Asgard.	-	-	
Panther DB	PTHR23073	Path	2	864	LECA	1.76	1.69	1.80		Asgard.	PSMC3	5702	SF5
Panther DB	PTHR23073	Path	2	492	Duplication	2.73	2.55	2.87		Asgard.	-	-	
Panther DB	PTHR23073	Path	2	721	Duplication	2.37	2.09	2.64		Asgard.	-	-	
Panther DB	PTHR23073	Path	2	722	LECA	1.76	1.68	1.80		Asgard.	PSMC1	5700	SF5
Panther DB	PTHR23073	Path	2	792	LECA	1.77	1.70	1.80		Asgard.	PSMC4	5704	SF5
Panther DB	PTHR23073	Path	2	493	Duplication	2.57	2.31	2.78		Asgard.	-	-	
Panther DB	PTHR23073	Path	2	632	LECA	1.78	1.72	1.80		Asgard.	PSMC6	5706	SF5
Panther DB	PTHR23073	Path	2	494	Duplication	2.42	2.13	2.68		Asgard.	-	-	
Panther DB	PTHR23073	Path	2	567	LECA	1.76	1.68	1.80		Asgard.	PSMC2	5701	SF5
Panther DB	PTHR23073	Path	2	495	LECA	1.77	1.70	1.80		Asgard.	PSMC5	5705	SF5
Panther DB	PTHR24071	Whole	2	422	nFECA divergence	2.92	2.92	2.92		Asgard.	-	-	

Panther DB	PTHR24071	Whole	2	423	Duplication	2.85	2.68	2.91		Asgard.	-	-	
Panther DB	PTHR24071	Whole	2	424	Duplication	2.63	2.26	2.87		Asgard.	-	-	
Panther DB	PTHR24071	Whole	2	570	LECA	0.74	0.64	0.90		Asgard.	RAB19/30/33B/33A	401409 27314 83452 9363	3
Panther DB	PTHR24071	Whole	2	425	Duplication	2.47	2.09	2.79		Asgard.	-	-	
Panther DB	PTHR24071	Whole	2	426	LECA	1.76	1.69	1.80		Asgard.	RAB25	57111	3
Panther DB	PTHR24071	Whole	2	463	Duplication	2.25	1.91	2.63		Asgard.	-	-	
Panther DB	PTHR24071	Whole	2	464	LECA	1.75	1.68	1.80		Asgard.	RAB5C	5878	3
Panther DB	PTHR24071	Whole	2	513	Duplication	1.92	1.76	2.25		Asgard.	-	-	
Panther DB	PTHR24071	Whole	2	514	LECA	1.76	1.68	1.80		Asgard.	RAB37	326624	3
Panther DB	PTHR24071	Whole	2	536	LECA	1.75	1.68	1.80		Asgard.	RAB3C	115827	3
Panther DB	PTHR24071	Whole	2	611	Duplication	2.62	2.32	2.84		Asgard.	-	-	
Panther DB	PTHR24071	Whole	2	730	Duplication	2.43	2.07	2.74		Asgard.	-	-	
Panther DB	PTHR24071	Whole	2	806	LECA	1.74	1.68	1.80		Asgard.	RAB6B	51560	3
Panther DB	PTHR24071	Whole	2	731	Duplication	2.22	1.86	2.60		Asgard.	-	-	
Panther DB	PTHR24071	Whole	2	781	LECA	1.75	1.68	1.80		Asgard.	RAB21	23011	3
Panther DB	PTHR24071	Whole	2	732	LECA	1.75	1.68	1.80		Asgard.	RAB5A/B/C	5869 5878 5868	3

Panther DB	PTHR24071	Whole	2	612	Duplication	2.40	2.01	2.74		Asgard.	-	-	
Panther DB	PTHR24071	Whole	2	613	LECA	1.77	1.71	1.80		Asgard.	RAB23/29/32/38	51715 8934 23682 10981	3
Panther DB	PTHR24071	Whole	2	659	LECA	1.85	1.73	2.11		Asgard.	RAB9A/9B/7A	51209 9367 7879	3
Panther DB	PTHR43272	Whole	1	182	BACT divergence	2.35	2.15	2.57		Bacterial		-	
Panther DB	PTHR43272	Whole	1	183	Duplication	1.98	1.87	2.12		Bacterial		-	
Panther DB	PTHR43272	Whole	1	184	LECA	1.74	1.67	1.80		Bacterial	ACSL3/4	2182 2181	4
Panther DB	PTHR43272	Whole	1	241	Duplication	1.82	1.74	1.92		Bacterial		-	
Panther DB	PTHR43272	Whole	1	242	LECA	1.73	1.67	1.80		Bacterial		-	
Panther DB	PTHR43272	Whole	1	259	LECA	1.71	1.67	1.79		Bacterial	ACSL1/5/6	2180 23305 51703	4
Panther DB	PTHR45916	Whole	2	95	nFECA divergence	2.92	2.92	2.92		Asgard.		-	
Panther DB	PTHR45916	Whole	2	96	Duplication	2.65	2.23	2.87		Asgard.		-	
Panther DB	PTHR45916	Whole	2	97	LECA	1.72	1.67	1.79		Asgard.	SMC6	79677	SF3
Panther DB	PTHR45916	Whole	2	141	LECA	1.70	1.67	1.77		Asgard.	SMC5	23137	SF3
PFAM	PF01472	Whole	2	204	nFECA divergence	2.92	2.92	2.92		Asgard.	-	-	

PFAM	PF01472	Whole	2	210	Duplication	2.51	2.19	2.77		Asgard.	-	-	
PFAM	PF01472	Whole	2	309	LECA	1.74	1.68	1.80		Asgard.	-	-	
PFAM	PF01472	Whole	2	211	LECA	1.76	1.69	1.80		Asgard.	MCTS1	28985	
Custom HMM	Q07864	Whole	2	253	nFECA divergence	2.92	2.92	2.92		Asgard.		-	
Custom HMM	Q07864	Whole	2	254	Duplication	2.65	2.48	2.79		Asgard.		-	
Custom HMM	Q07864	Whole	2	386	LECA	1.72	1.67	1.79		Asgard.	POLE	5426	SF2
Custom HMM	Q07864	Whole	2	254	Duplication	2.65	2.48	2.79		Asgard.		-	
Custom HMM	Q07864	Whole	2	256	LECA	1.78	1.71	1.80		Asgard.	POLA1	5422	SF2
Custom HMM	Q07864	Whole	2	304	LECA	1.78	1.74	1.81		Asgard.	POLD1	5424	SF2
Custom HMM	Q96DA6	Whole	2	147	mFECA divergence	2.24	2.24	2.24		Alpha.	-	-	
Custom HMM	Q96DA6	Whole	2	148	Duplication	2.15	1.99	2.24		Alpha.	-	-	
Custom HMM	Q96DA6	Whole	2	149	partial LECA	1.74	1.68	1.80		Alpha.	DNAJC15,TIM14	29103 131118	4
Custom HMM	Q96DA6	Whole	2	215	LECA	1.73	1.67	1.79		Alpha.	PAM16	51025	4
Custom HMM	Q96HY7	Whole	2	211	mFECA divergence	2.24	2.24	2.24		Alpha.	-	-	
Custom HMM	Q96HY7	Whole	2	212	Duplication	2.11	1.99	2.19		Alpha.	-	-	
Custom HMM	Q96HY7	Whole	2	304	LECA	1.73	1.67	1.80		Alpha.	DHTKD1	55526	4
Custom HMM	Q96HY7	Whole	2	213	LECA	1.75	1.68	1.80		Alpha.	OGDH OGDHL	4967 55753	4

PFAM	PF00428	Whole	2	218	nFECA divergence	2.92	2.92	2.92		Asgard.	-	-	
PFAM	PF00428	Whole	2	219	Duplication	2.36	1.84	2.86		Asgard.	-	-	
PFAM	PF00428	Whole	2	306	LECA	1.77	1.69	1.80		Asgard.	RPLP1	6176	SF6
PFAM	PF00428	Whole	2	220	LECA	1.75	1.68	1.80		Asgard.	RPLP2	6181	SF6
PFAM	PF00466	Whole	2	245	nFECA divergence	2.92	2.92	2.92		Asgard.	-	-	
PFAM	PF00466	Whole	2	246	Duplication	2.56	2.22	2.83		Asgard.	-	-	
PFAM	PF00466	Whole	2	339	LECA	1.78	1.74	1.81		Asgard.	MRTO4	51154	SF6
PFAM	PF00466	Whole	2	247	LECA	1.78	1.72	1.80		Asgard.	RPLP0	6175	SF6
PFAM	PF01246	Whole	2	205	nFECA divergence	2.92	2.92	2.92		Asgard.	-	-	
PFAM	PF01246	Whole	2	206	Duplication	2.42	2.08	2.75		Asgard.	-	-	
PFAM	PF01246	Whole	2	207	LECA	1.76	1.68	1.80		Asgard.	RSL24D1	51187	SF6
PFAM	PF01246	Whole	2	289	LECA	1.76	1.69	1.80		Asgard.	RPL24	6152	SF6
PFAM	PF01248	Whole	2	282	nFECA divergence	2.92	2.92	2.92		Asgard.	-	-	
PFAM	PF01248	Whole	2	283	Duplication	2.76	2.48	2.91		Asgard.	-	-	
PFAM	PF01248	Whole	2	441	LECA	1.76	1.69	1.80		Asgard.	RPS12	6206	SF3
PFAM	PF01248	Whole	2	284	Duplication	2.62	2.29	2.86		Asgard.		-	
PFAM	PF01248	Whole	2	285	LECA	1.77	1.70	1.80		Asgard.	NHP2	55651	SF3
PFAM	PF01248	Whole	2	347	LECA	2.27	1.89	2.66		Asgard.	SNU13	4809	SF3

PFAM	PF00163	Whole	2	198	nFECA divergence	2.92	2.92	2.92		Asgard.	-	-	
PFAM	PF00163	Whole	2	199	Duplication	2.71	2.40	2.90		Asgard.	-	-	
PFAM	PF00163	Whole	2	288	LECA	1.78	1.72	1.80		Asgard.	IMP3	55272	SF6
PFAM	PF00163	Whole	2	200	LECA	1.76	1.68	1.80		Asgard.	RPS9	6203	SF6
PFAM	PF07521	Whole	2	221	nFECA divergence	2.92	2.92	2.92		Asgard.	-	-	
PFAM	PF07521	Whole	2	222	Duplication	2.54	2.28	2.75		Asgard.	-	-	
PFAM	PF07521	Whole	2	354	LECA	1.73	1.67	1.80		Asgard.	CPSF2	53981	SF2
PFAM	PF07521	Whole	2	223	Duplication	2.22	1.96	2.51		Asgard.	-	-	
PFAM	PF07521	Whole	2	301	LECA	1.74	1.67	1.80		Asgard.	N	54973	SF2
PFAM	PF07521	Whole	2	224	LECA	1.79	1.75	1.81		Asgard.	CPSF3	51692	SF2
PFAM	PF09414	Whole	2	148	nFECA divergence	2.92	2.92	2.92		Asgard.	-	-	
PFAM	PF09414	Whole	2	149	Duplication	2.63	2.43	2.78		Asgard.	-	-	
PFAM	PF09414	Whole	2	210	LECA	1.74	1.71	1.81		Asgard.	LIG1	3978	SF4
PFAM	PF09414	Whole	2	150	Duplication	2.3	2.1	2.51		Asgard.	-	-	
PFAM	PF09414	Whole	2	184	LECA	1.76	1.71	1.82		Asgard.	LIG3	3980	SF4
PFAM	PF09414	Whole	2	151	LECA	1.61	1.46	1.72		Asgard.	LIG4	3981	SF4
PFAM	PF01000	Whole	2	184	nFECA divergence	2.92	2.92	2.92		Asgard.	-	-	
PFAM	PF01000	Whole	2	185	Duplication	2.44	2.15	2.71		Asgard.	-	-	
PFAM	PF01000	Whole	2	186	LECA	1.74	1.68	1.80		Asgard.	POLR1C	9533	2, SF2

PFAM	PF01000	Whole	2	255	LECA	1.77	1.70	1.80		Asgard.	POLR2C	5432	2, SF2
PFAM	PF01193	Subtree	2	181	nFECA divergence	2.92	2.92	2.92		Asgard.		-	
PFAM	PF01193	Subtree	2	182	Duplication	2.27	1.88	2.73		Asgard.		-	
PFAM	PF01193	Subtree	2	253	LECA	1.76	1.69	1.80		Asgard.	POLR2J/J2	246721 5439	2, SF2
PFAM	PF01193	Subtree	2	183	LECA	1.75	1.68	1.80		Asgard.	POLR1D	51082	2, SF2
PFAM	PF04998	Whole	2	235	nFECA divergence	2.92	2.92	2.92		Asgard.	-	-	
PFAM	PF04998	Whole	2	236	Duplication	2.54	2.32	2.73		Asgard.	-	-	
PFAM	PF04998	Whole	2	237	LECA	1.76	1.69	1.80		Asgard.	POLR3A	11128	2, SF2
PFAM	PF04998	Whole	2	303	Duplication	2.35	2.12	2.57		Asgard.	-	-	
PFAM	PF04998	Whole	2	378	LECA	1.75	1.68	1.80		Asgard.	POLR1A	25885	2, SF2
PFAM	PF04998	Whole	2	304	LECA	1.73	1.67	1.80		Asgard.	POLR2A	5430	2, SF2
PFAM	PF03874	Whole	2	111	nFECA divergence	2.92	2.92	2.92		Asgard.	-	-	
PFAM	PF03874	Whole	2	112	Duplication	2.60	2.26	2.85		Asgard.	-	-	
PFAM	PF03874	Whole	2	113	LECA	1.75	1.68	1.80		Asgard.	CRCP	27297	2, SF2
PFAM	PF03874	Whole	2	138	LECA	1.74	1.68	1.80		Asgard.	POLR2D	5433	2, SF2
PFAM	PF01138	Subtree	2	187	nFECA divergence	2.92	2.92	2.92		Asgard.	-	-	
PFAM	PF01138	Subtree	2	188	Duplication	2.65	2.31	2.88		Asgard.	-	-	
PFAM	PF01138	Subtree	2	286	LECA	1.73	1.67	1.80		Asgard.	EXOSC9	5393	SF3

PFAM	PF01138	Subtree	2	189	Duplication	2.36	2.01	2.72		Asgard.		-	
PFAM	PF01138	Subtree	2	243	LECA	1.72	1.67	1.79		Asgard.	EXOSC7	23016	SF3
PFAM	PF01138	Subtree	2	190	LECA	1.75	1.68	1.80		Asgard.	EXOSC8	11340	SF3
PFAM	PF01138	Subtree	2	177	nFECA divergence	2.92	2.92	2.92		Asgard.	-	-	
PFAM	PF01138	Subtree	2	178	Duplication	2.65	2.34	2.86		Asgard.	-	-	
PFAM	PF01138	Subtree	2	179	Duplication	2.33	2.02	2.65		Asgard.	-	-	
PFAM	PF01138	Subtree	2	180	LECA	1.75	1.68	1.80		Asgard.	EXOSC5	56915	SF3
PFAM	PF01138	Subtree	2	197	LECA	1.75	1.68	1.80		Asgard.	EXOSC6	118460	SF3
PFAM	PF01138	Subtree	2	234	LECA	1.76	1.68	1.80		Asgard.	EXOSC4	54512	SF3
PFAM	PF01137	Whole	2	146	nFECA divergence	2.92	2.92	2.92		Asgard.	-	-	
PFAM	PF01137	Whole	2	147	Duplication	2.58	2.24	2.82		Asgard.	-	-	
PFAM	PF01137	Whole	2	148	LECA	1.74	1.68	1.80		Asgard.	RTCA	8634	
PFAM	PF01137	Whole	2	180	LECA	1.72	1.67	1.79		Asgard.	RCL1	10171	
PFAM	PF03876	Whole	2	253	nFECA divergence	2.92	2.92	2.92		Asgard.	-	-	
PFAM	PF03876	Whole	2	254	Duplication	2.63	2.27	2.88		Asgard.	-	-	
PFAM	PF03876	Whole	2	255	LECA	1.77	1.70	1.80		Asgard.	POLR2G	5436	2, SF2
PFAM	PF03876	Whole	2	336	Duplication	2.26	1.91	2.66		Asgard.	-	-	
PFAM	PF03876	Whole	2	414	LECA	1.74	1.68	1.80		Asgard.	POLR1F	221830	2, SF2
PFAM	PF03876	Whole	2	337	LECA	1.76	1.68	1.80		Asgard.	POLR3H	171568	2, SF2

PFAM	PF05916	Whole	2	143	nFECA divergence	2.92	2.92	2.92		Asgard.	-	-	
PFAM	PF05916	Whole	2	151	Duplication	2.72	2.38	2.91		Asgard.	-	-	
PFAM	PF05916	Whole	2	152	LECA	1.73	1.67	1.80		Asgard.	GINS3	64785	SF2
PFAM	PF05916	Whole	2	209	LECA	1.75	1.68	1.80		Asgard.	GINS2	51659	SF2
PFAM	PF06470	Whole	2	245	nFECA divergence	2.92	2.92	2.92		Asgard.	-	-	
PFAM	PF06470	Whole	2	246	Duplication	2.72	2.55	2.84		Asgard.	-	-	
PFAM	PF06470	Whole	2	247	Duplication	2.42	2.18	2.64		Asgard.	-	-	
PFAM	PF06470	Whole	2	248	LECA	1.74	1.67	1.80		Asgard.	SMC3	9126	SF3
PFAM	PF06470	Whole	2	297	LECA	1.75	1.68	1.80		Asgard.	SMC2	10592	SF3
PFAM	PF06470	Whole	2	349	Duplication	2.38	2.12	2.61		Asgard.	-	-	
PFAM	PF06470	Whole	2	350	LECA	1.72	1.67	1.79		Asgard.	SMC4	10051	SF3
PFAM	PF06470	Whole	2	402	LECA	1.72	1.67	1.79		Asgard.	SMC1A/B	8243<br>27127	SF3
PFAM	PF03357	Subtree	2	314	nFECA divergence	2.92	2.92	2.92		Asgard.	-	-	
PFAM	PF03357	Subtree	2	315	Duplication	2.67	2.37	2.89		Asgard.	-	-	
PFAM	PF03357	Subtree	2	504	Duplication	2.34	1.97	2.72		Asgard.	-	-	
PFAM	PF03357	Subtree	2	575	LECA	1.76	1.69	1.80		Asgard.	CHMP7	91782	SF3
PFAM	PF03357	Subtree	2	505	LECA	1.77	1.69	1.80		Asgard.	CHMP6	79643	SF3
PFAM	PF03357	Subtree	2	316	Duplication	2.43	2.06	2.77		Asgard.	-	-	
PFAM	PF03357	Subtree	2	414	LECA	1.77	1.70	1.80		Asgard.	CHMP5	51510	SF3

PFAM	PF03357	Subtree	2	317	LECA	1.77	1.69	1.80		Asgard.	CHMP4A/B/BP1/C	29082<br>92421<br>100307126<br>128866	SF3
PFAM	PF03357	Subtree	2	337	nFECA divergence	2.92	2.92	2.92		Asgard.	-	-	
PFAM	PF03357	Subtree	2	338	Duplication	2.76	2.50	2.90		Asgard.	-	-	
PFAM	PF03357	Subtree	2	551	LECA	1.74	1.68	1.80		Asgard.	CHMP1A/B	5119<br>57132	SF3
PFAM	PF03357	Subtree	2	339	Duplication	2.45	2.15	2.73		Asgard.	-	-	
PFAM	PF03357	Subtree	2	468	LECA	1.77	1.69	1.80		Asgard.	CHMP2A	27243	SF3
PFAM	PF03357	Subtree	2	340	Duplication	2.27	1.98	2.59		Asgard.	-	-	
PFAM	PF03357	Subtree	2	341	LECA	1.76	1.69	1.80		Asgard.	CHMP2B	25978	SF3
PFAM	PF03357	Subtree	2	404	LECA	1.75	1.68	1.80		Asgard.	CHMP3	51652	SF3
PFAM	PF01253	Whole	2	356	nFECA divergence	2.92	2.92	2.92		Asgard.	-	-	
PFAM	PF01253	Whole	2	357	Duplication	2.65	2.29	2.89		Asgard.	-	-	
PFAM	PF01253	Whole	2	358	LECA	1.75	1.68	1.80		Asgard.	EIF1/1B	10289<br>10209	SF6
PFAM	PF01253	Whole	2	460	Duplication	2.33	1.99	2.70		Asgard.	-	-	
PFAM	PF01253	Whole	2	562	LECA	1.77	1.70	1.80		Asgard.	EIF2D	1939	SF6
PFAM	PF01253	Whole	2	461	LECA	1.78	1.73	1.81		Asgard.	DENR	8562	SF6
PFAM	PF02969	Whole	2	174	nFECA divergence	2.92	2.92	2.92		Asgard.	-	-	
PFAM	PF02969	Whole	2	175	Duplication	2.49	2.05	2.85		Asgard.	-	-	

PFAM	PF02969	Whole	2	273	LECA	1.76	1.71	1.82		Asgard.	H4	-	
PFAM	PF02969	Whole	2	176	LECA	1.78	1.71	1.83		Asgard.	TAF6/L	6878 10629	SF2
PFAM	PF00382	Whole	2	195	nFECA divergence	2.92	2.92	2.92		Asgard.	-	-	
PFAM	PF00382	Whole	2	196	Duplication	2.60	2.16	2.90		Asgard.	-	-	
PFAM	PF00382	Whole	2	297	LECA	1.77	1.71	1.80		Asgard.	GTF2B	2959	2, SF2
PFAM	PF00382	Whole	2	197	LECA	1.77	1.70	1.80		Asgard.	BRF1	2972	2, SF2
PFAM	PF01096	Whole	2	380	nFECA divergence	2.92	2.92	2.92		Asgard.	-	-	
PFAM	PF01096	Whole	2	381	Duplication	2.74	2.46	2.90		Asgard.	-	-	
PFAM	PF01096	Whole	2	636	LECA	1.75	1.68	1.80		Asgard.	POLR3K	51728	2, SF2
PFAM	PF01096	Whole	2	382	Duplication	2.60	2.27	2.85		Asgard.	-	-	
PFAM	PF01096	Whole	2	560	LECA	1.77	1.70	1.80		Asgard.	POLR1H	30834	2, SF2
PFAM	PF01096	Whole	2	383	Duplication	2.33	1.96	2.70		Asgard.	-	-	
PFAM	PF01096	Whole	2	488	LECA	1.75	1.68	1.80		Asgard.	POLR2I	5438	2, SF2
PFAM	PF01096	Whole	2	384	LECA	1.75	1.68	1.80		Asgard.	TCEA1/2/3	6917 6920 6919	2, SF2
PFAM	PF02824	Path	2	212	nFECA divergence	2.92	2.92	2.92		Asgard.	-	-	
PFAM	PF02824	Path	2	213	Duplication	2.36	2.10	2.61		Asgard.	-	-	
PFAM	PF02824	Path	2	214	LECA	1.76	1.70	1.80		Asgard.	DRG2	1819	
PFAM	PF02824	Path	2	298	LECA	1.76	1.69	1.80		Asgard.	DRG1	4733	

PFAM	PF04280	Whole	1	143	mFECA divergence	2.24	2.24	2.24		Alpha.	-	-	
PFAM	PF04280	Whole	1	170	Duplication	2.12	2.00	2.22		Alpha.	-	-	
PFAM	PF04280	Whole	1	171	LECA	1.75	1.68	1.80		Alpha.	MRPL45	84311	4
PFAM	PF04280	Whole	1	207	LECA	1.75	1.68	1.80		Alpha.	TIMM44	10469	4
PFAM	PF17856	Whole	2	194	nFECA divergence	3.09	2.79	3.32		Archaeal	-	-	
PFAM	PF17856	Whole	2	195	Duplication	2.63	2.29	2.96		Archaeal	-	-	
PFAM	PF17856	Whole	2	196	LECA	1.77	1.71	1.80		Archaeal	RUVBL1	8607	
PFAM	PF17856	Whole	2	283	LECA	1.78	1.72	1.80		Archaeal	RUVBL2	10856	
PFAM	PF01997	Whole	2	113	nFECA divergence	2.92	2.92	2.92		Asgard.	-	-	
PFAM	PF01997	Whole	2	114	Duplication	2.30	2.02	2.59		Asgard.	-	-	
PFAM	PF01997	Whole	2	163	LECA	1.74	1.67	1.80		Asgard.	TSNAX	7257	SF3
PFAM	PF01997	Whole	2	115	LECA	1.72	1.67	1.79		Asgard.	TSN	7247	SF3
PFAM	PF04051	Whole	2	273	ARCH divergence	3.23	2.77	3.62		Asgard.	-	-	
PFAM	PF04051	Whole	2	274	Duplication	2.83	2.54	3.00		Asgard.	-	-	
PFAM	PF04051	Whole	2	275	partial LECA	1.76	1.69	1.80		Asgard.	TRAPPC3/L	100128327 27095	3
PFAM	PF04051	Whole	2	376	Duplication	2.50	2.13	2.83		Asgard.	-	-	
PFAM	PF04051	Whole	2	377	partial LECA	1.76	1.68	1.80		Asgard.	TRAPPC6A/B	122553 79090	3
PFAM	PF04051	Whole	2	454	LECA	1.76	1.69	1.80		Asgard.	TRAPPC5	126003	3

PFAM	PF01974	Whole	1	173	ARCH divergence	3.13	2.82	3.36		Archaeal	-	-	
PFAM	PF01974	Whole	1	174	Duplication	2.33	1.89	2.92		Archaeal	-	-	
PFAM	PF01974	Whole	1	236	LECA	1.76	1.69	1.80		Archaeal	TSEN2	80746	SF3
PFAM	PF01974	Whole	1	175	LECA	1.77	1.71	1.80		Archaeal	TSEN34	79042	SF3
PFAM	PF01142	Whole	1	144	ARCH divergence	2.75	2.35	3.11		Archaeal	-	-	
PFAM	PF01142	Whole	1	145	Duplication	1.90	1.78	2.10		Archaeal	-	-	
PFAM	PF01142	Whole	1	218	LECA	1.72	1.67	1.80		Archaeal	PUS7L	83448	SF3
PFAM	PF01142	Whole	1	146	LECA	1.77	1.69	1.80		Archaeal	PUS7	54517	SF3
PFAM	PF14681	Whole	1	230	BACT divergence	2.44	2.16	2.74		Bacterial	-	-	
PFAM	PF14681	Whole	1	231	Duplication	1.99	1.83	2.22		Bacterial	-	-	
PFAM	PF14681	Whole	1	232	partial LECA	1.75	1.68	1.80		Bacterial	UPRT	139596	
PFAM	PF14681	Whole	1	290	LECA	1.75	1.68	1.80		Bacterial	UCKL1	54963	
PFAM	PF00179	Whole	2	485	nFECA divergence	2.92	2.92	2.92		Asgard.	-	-	
PFAM	PF00179	Whole	2	486	Duplication	2.86	2.73	2.91		Asgard.	-	-	
PFAM	PF00179	Whole	2	912	LECA	1.76	1.68	1.80		Asgard.	UBE2H	7328	SF5
PFAM	PF00179	Whole	2	487	Duplication	2.80	2.63	2.90		Asgard.	-	-	
PFAM	PF00179	Whole	2	744	Duplication	2.63	2.28	2.85		Asgard.	-	-	
PFAM	PF00179	Whole	2	880	LECA	1.76	1.68	1.80		Asgard.	UBE2K	3093	SF5

PFAM	PF00179	Whole	2	745	Duplication	2.45	2.07	2.76		Asgard.	-	-	
PFAM	PF00179	Whole	2	834	LECA	1.76	1.68	1.80		Asgard.	UBE2N/L	7334 389898	SF5
PFAM	PF00179	Whole	2	746	Duplication	2.26	1.91	2.63		Asgard.	-	-	
PFAM	PF00179	Whole	2	818	LECA	1.75	1.68	1.80		Asgard.	UBE2T	29089	SF5
PFAM	PF00179	Whole	2	747	Duplication	2.04	1.78	2.44		Asgard.	-	-	
PFAM	PF00179	Whole	2	792	LECA	1.74	1.68	1.80		Asgard.	UBE2E1/2/3	10477 7325 7324	SF5
PFAM	PF00179	Whole	2	748	LECA	1.75	1.68	1.80		Asgard.	UBE2D1/2/4	51619 7321 7322	SF5
PFAM	PF00179	Whole	2	488	Duplication	2.72	2.48	2.87		Asgard.	-	-	
PFAM	PF00179	Whole	2	489	Duplication	2.43	1.97	2.78		Asgard.	-	-	
PFAM	PF00179	Whole	2	536	LECA	1.75	1.68	1.80		Asgard.	UBE2S	27338	SF5
PFAM	PF00179	Whole	2	490	LECA	1.75	1.68	1.80		Asgard.	UBE2L3/6	7332 9246	SF5
PFAM	PF00179	Whole	2	564	Duplication	2.61	2.32	2.82		Asgard.	-	-	
PFAM	PF00179	Whole	2	716	LECA	1.76	1.68	1.80		Asgard.	UBE2C	11065	SF5
PFAM	PF00179	Whole	2	565	Duplication	2.50	2.19	2.75		Asgard.	-	-	
PFAM	PF00179	Whole	2	681	LECA	1.74	1.68	1.80		Asgard.	UBE2A	7319	SF5
PFAM	PF00179	Whole	2	566	Duplication	2.40	2.07	2.69		Asgard.	-	-	
PFAM	PF00179	Whole	2	631	LECA	1.76	1.69	1.80		Asgard.	UBE2I	7329	SF5
PFAM	PF00179	Whole	2	567	Duplication	2.24	1.88	2.58		Asgard.	-	-	
PFAM	PF00179	Whole	2	609	LECA	2.08	1.63	2.48		Asgard.	UBE2G2	7327	SF5

PFAM	PF00179	Whole	2	568	LECA	1.76	1.69	1.80		Asgard.	UBE2G1	7326	SF5
Custom HMM	XP_006517743	Whole	2	155	mFECA divergence	2.24	2.24	2.24		Alpha.	-	-	
Custom HMM	XP_006517743	Whole	2	156	Duplication	2.20	2.12	2.24		Alpha.	-	-	
Custom HMM	XP_006517743	Whole	2	257	LECA	1.70	1.67	1.77		Alpha.	POLK	51426	SF4
Custom HMM	XP_006517743	Whole	2	157	Duplication	2.11	2.01	2.19		Alpha.	-	-	
Custom HMM	XP_006517743	Whole	2	225	LECA	1.71	1.67	1.79		Alpha.	REV1	51455	SF4
Custom HMM	XP_006517743	Whole	2	158	Duplication	1.89	1.80	1.99		Alpha.	-	-	
Custom HMM	XP_006517743	Whole	2	202	LECA	1.74	1.68	1.80		Alpha.	POLI	11201	SF4
Custom HMM	XP_006517743	Whole	2	159	LECA	1.71	1.67	1.78		Alpha.	POLH	5429	SF4
PFAM	PF00752	Whole	2	313	nFECA divergence	2.92	2.92	2.92		Asgard.		-	
PFAM	PF00752	Whole	2	314	Duplication	2.72	2.46	2.88		Asgard.		-	
PFAM	PF00752	Whole	2	481	LECA	1.76	1.69	1.80		Asgard.	FEN1	2237	SF4
PFAM	PF00752	Whole	2	315	Duplication	2.42	2.15	2.69		Asgard.		-	
PFAM	PF00752	Whole	2	316	LECA	1.78	1.71	1.80		Asgard.	EXO1	9156	SF4
PFAM	PF00752	Whole	2	395	Duplication	2.12	1.90	2.40		Asgard.		-	
PFAM	PF00752	Whole	2	449	LECA	1.76	1.68	1.80		Asgard.	GEN1	348654	SF4
PFAM	PF00752	Whole	2	396	LECA	1.74	1.67	1.80		Asgard.	ERCC5	2073	SF4
PFAM	PF04893	Whole	2	269	nFECA divergence	2.92	2.92	2.92		Asgard.	-	-	

PFAM	PF04893	Whole	2	270	Duplication	2.78	2.55	2.91		Asgard.	-	-	
PFAM	PF04893	Whole	2	441	LECA	1.75	1.68	1.80		Asgard.	YIPF1/2	54432<br>78992	3
PFAM	PF04893	Whole	2	271	Duplication	2.66	2.39	2.86		Asgard.	-	-	
PFAM	PF04893	Whole	2	414	LECA	1.74	1.67	1.80		Asgard.	YIPF4	84272	3
PFAM	PF04893	Whole	2	272	Duplication	2.46	2.16	2.74		Asgard.	-	-	
PFAM	PF04893	Whole	2	348	LECA	1.75	1.68	1.80		Asgard.	YIPF6	286451	3
PFAM	PF04893	Whole	2	273	LECA	1.75	1.68	1.80		Asgard.	YIPF5/7	81555<br>285525	3
PFAM	PF01428	Whole	2	322	nFECA divergence	2.92	2.92	2.92		Asgard.	-	-	
PFAM	PF01428	Whole	2	323	Duplication	2.60	2.24	2.88		Asgard.	-	-	
PFAM	PF01428	Whole	2	324	LECA	1.78	1.71	1.80		Asgard.	ZFAND1/2A/2B	79752<br>90637<br>130617	
PFAM	PF01428	Whole	2	427	Duplication	2.28	1.98	2.62		Asgard.	-	-	
PFAM	PF01428	Whole	2	569	LECA	1.74	1.67	1.80		Asgard.	IGHMBP2	3508	
PFAM	PF01428	Whole	2	428	Duplication	2.10	1.85	2.44		Asgard.	-	-	
PFAM	PF01428	Whole	2	429	LECA	1.06	0.78	1.27		Asgard.	ZFAND4	93550	
PFAM	PF01428	Whole	2	450	Duplication	1.82	1.72	1.99		Asgard.	-	-	
PFAM	PF01428	Whole	2	451	LECA	1.73	1.67	1.80		Asgard.	ZFAND3	60685	
PFAM	PF01428	Whole	2	478	LECA	1.75	1.68	1.80		Asgard.	ZFAND5/6	54469<br>7763	
PFAM	PF06723	Whole	2	186	BACT divergence	2.78	2.47	3.05		Bacterial	-	-	

PFAM	PF06724	Whole	2	187	Duplication	2.01	1.86	2.23		Bacterial	-	-
PFAM	PF06725	Whole	2	251	partial LECA	1.77	1.71	1.82		Bacterial	-	-
PFAM	PF06726	Whole	2	188	partial LECA	1.80	1.73	1.83		Bacterial	-	-

849

Supplementary Bibliography

1. He, D. *et al.* An alternative root for the eukaryote tree of life. *Curr. Biol.* **24**, 465–470 (2014).
2. Derelle, R. *et al.* Bacterial proteins pinpoint a single eukaryotic root. *Proc. Natl. Acad. Sci. U. S. A.* **112**, E693–9 (2015).
3. Eme, L. *et al.* Inference and reconstruction of the heimdallarchaeial ancestry of eukaryotes. *Nature* **618**, 992–999 (2023).
4. Miller, J. M. & Enemark, E. J. Archaeal MCM Proteins as an Analog for the Eukaryotic Mcm2-7 Helicase to Reveal Essential Features of Structure and Function. *Archaea* **2015**, 305497 (2015).
5. Kamada, K. The GINS complex: structure and function. *Subcell. Biochem.* **62**, 135–156 (2012).
6. Bell, S. D. & Botchan, M. R. The minichromosome maintenance replicative helicase. *Cold Spring Harb. Perspect. Biol.* **5**, a012807 (2013).
7. Han, X., Aslanian, A., Fu, K., Tsuji, T. & Zhang, Y. The interaction between checkpoint kinase 1 (Chk1) and the minichromosome maintenance (MCM) complex is required for DNA damage-induced Chk1 phosphorylation. *J. Biol. Chem.* **289**, 24716–24723 (2014).
8. Werner, F. & Grohmann, D. Evolution of multisubunit RNA polymerases in the three domains of life. *Nat Rev Microbiol* **9**, 85–98 (2011).
9. Pereira-Leal, J. B. & Teichmann, S. A. Novel specificities emerge by stepwise duplication of functional modules. *Genome Res.* **15**, 552–559 (2005).
10. Vosseberg, J. & Snel, B. Domestication of self-splicing introns during eukaryogenesis: the rise of the complex spliceosomal machinery. *Biol. Direct* **12**, 30 (2017).
11. Zimmerly, S. & Semper, C. Evolution of group II introns. *Mob. DNA* **6**, 7 (2015).
12. Spang, A. *et al.* Complex archaea that bridge the gap between prokaryotes and eukaryotes. *Nature* **521**, 173–179 (2015).

13. Mura, C., Randolph, P. S., Patterson, J. & Cozen, A. E. Archaeal and eukaryotic homologs of Hfq: A structural and evolutionary perspective on Sm function. *RNA Biol.* **10**, 636–651 (2013).
14. Veretnik, S., Wills, C., Youkharibache, P., Valas, R. E. & Bourne, P. E. Sm/Lsm genes provide a glimpse into the early evolution of the spliceosome. *PLoS Comput. Biol.* **5**, e1000315 (2009).
15. Stevens, K. M. *et al.* Histone variants in archaea and the evolution of combinatorial chromatin complexity. *Proc Natl Acad Sci U S A* **117**, 33384–33395 (2020).
16. Yoshinaga, M. & Inagaki, Y. Ubiquity and origins of structural maintenance of chromosomes (SMC) proteins in eukaryotes. *Genome Biol. Evol.* **13**, evab256 (2021).
17. van Hooff, J. J. E., Raas, M. W. D., Tromer, E. C. & Eme, L. Repeated duplications and losses shaped SMC complex evolution from archaeal ancestors to modern eukaryotes. *Cell Rep* **44**, 115855 (2025).
18. Soppa, J. Prokaryotic structural maintenance of chromosomes (SMC) proteins: distribution, phylogeny, and comparison with MukBs and additional prokaryotic and eukaryotic coiled-coil proteins. *Gene* **278**, 253–264 (2001).
19. Minaker, S. W., Filiatrault, M. C., Ben-Aroya, S., Hieter, P. & Stirling, P. C. Biogenesis of RNA polymerases II and III requires the conserved GPN small GTPases in Saccharomyces cerevisiae. *Genetics* **193**, 853–864 (2013).
20. Cubeñas-Potts, C. & Matunis, M. J. SUMO: a multifaceted modifier of chromatin structure and function. *Dev. Cell* **24**, 1–12 (2013).
21. Criscuolo, A. & Gribaldo, S. BMGE (Block Mapping and Gathering with Entropy): a new software for selection of phylogenetic informative regions from multiple sequence alignments. *BMC Evol. Biol.* **10**, 210 (2010).
22. Camacho, C. *et al.* BLAST+: architecture and applications. *BMC Bioinformatics* **10**, 421 (2009).
23. Fu, L., Niu, B., Zhu, Z., Wu, S. & Li, W. CD-HIT: accelerated for clustering the next-generation sequencing data. *Bioinformatics* **28**, 3150–3152 (2012).

24. Buchfink, B., Reuter, K. & Drost, H.-G. Sensitive protein alignments at tree-of-life scale using DIAMOND. *Nat. Methods* **18**, 366–368 (2021).
25. Eddy, S. R. Accelerated profile HMM searches. *PLoS Comput. Biol.* **7**, e1002195 (2011).
26. Minh, B. Q. *et al.* IQ-TREE 2: New models and efficient methods for phylogenetic inference in the genomic era. *Mol. Biol. Evol.* **37**, 1530–1534 (2020).
27. Katoh, K. & Standley, D. M. MAFFT multiple sequence alignment software version 7: improvements in performance and usability. *Mol. Biol. Evol.* **30**, 772–780 (2013).
28. Gouveia-Oliveira, R., Sackett, P. W. & Pedersen, A. G. MaxAlign: maximizing usable data in an alignment. *BMC Bioinformatics* **8**, 312 (2007).
29. Van Dongen, S. Graph clustering via a discrete uncoupling process. *SIAM J. Matrix Anal. Appl.* **30**, 121–141 (2008).
30. dos Reis, M. & Yang, Z. Approximate likelihood calculation on a phylogeny for Bayesian estimation of divergence times. *Mol. Biol. Evol.* **28**, 2161–2172 (2011).
31. Jeffares, D. C., Tomiczek, B., Sojo, V. & dos Reis, M. A beginners guide to estimating the non-synonymous to synonymous rate ratio of all protein-coding genes in a genome. *Methods Mol. Biol.* **1201**, 65–90 (2015).
32. Simonsen, M. & Pedersen, C. N. S. Rapid computation of distance estimators from nucleotide and amino acid alignments. in *Proceedings of the 2011 ACM Symposium on Applied Computing* 89–93 (ACM, New York, NY, USA, 2011).
33. Comte, N. *et al.* Treerecs: an integrated phylogenetic tool, from sequences to reconciliations. *Bioinformatics* **36**, 4822–4824 (2020).
34. Neveu, E., Khalifeh, D., Salamin, N. & Fasshauer, D. Prototypic SNARE Proteins Are Encoded in the Genomes of Heimdallarchaeota, Potentially Bridging the Gap between the Prokaryotes and Eukaryotes. *Curr. Biol.* **30**, 2468–2480.e5 (2020).
